# Navigating the Ethereal Tightrope: The Nanogenerator Manipulates Neurons for Immune Equilibrium

**DOI:** 10.1002/advs.202512284

**Published:** 2026-02-09

**Authors:** Jia Du, Rui Deng, Ya Wu, Hong‐Xia Ren, Zong‐Hong Lin, Yang‐Bao Miao

**Affiliations:** ^1^ Department of Haematology Sichuan Academy of Medical Sciences & Sichuan Provincial People's Hospital School of Medicine of University of Electronic Science and Technology of China Chengdu China; ^2^ Department of Vascular Surgery The Affiliated Hospital of Southwest Medical University Luzhou China; ^3^ College of Light Industry and Engineering Sichuan Technology & Business College Chengdu China; ^4^ Metabolic Vascular Disease Key Laboratory of Sichuan Province The Affiliated Hospital of Southwest Medical University Luzhou China; ^5^ Department of Biomedical Engineering National Taiwan University Taipei Taiwan

**Keywords:** nanogenerators, neuroimmunomodulation, neurological disease therapy, self‐powered neuromodulation, vagus nerve stimulation

## Abstract

The intricacies of neuroimmunity underscore its pivotal role in the onset and progression of neurological diseases, yet its precise modulation remains a formidable challenge. The emergence of nanogenerators offers a promising avenue to overcome these obstacles, introducing a new paradigm for regulating neural immune responses. This review delves into the complex physiological landscape of neuroimmunity, emphasizing its profound impact on overall health and disease outcomes. It systematically examines the mechanisms by which nanogenerators interact with and modulate neuroimmune processes, while also charting key developmental milestones, synthesis strategies, and classification frameworks of nanogenerators. Particular attention is given to the application of nanogenerators in neuroimmunomodulation, critically analyzing current achievements, persistent challenges, and future directions. Furthermore, the review highlights the potential clinical translation of nanogenerators, notably their capacity to stimulate the vagus nerve and activate the brain's immune system, offering innovative therapeutic strategies for a spectrum of neurological disorders, including epilepsy, PD, AD, and stroke. As we navigate the ethereal tightrope of immune homeostasis, nanogenerators emerge as beacons of hope, heralding a future in which precise, targeted modulation of neuroimmunity may evolve from conceptual possibility to clinical reality.

## Graphical abstract

This review explores how nanogenerators modulate neuroimmune responses, offering innovative strategies for treating neurological disorders. By interfacing with neural pathways, they enable precise control of immune activity, especially via vagus nerve stimulation. Nanogenerators represent a transformative approach toward achieving immune balance in complex neurological conditions.

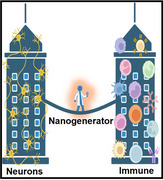

## 1 Introduction

The intricate relationship between the nervous and immune systems, collectively referred to as neuroimmunity, is fundamental to maintaining physiological homeostasis and responding to disease [1]. Emerging neuromodulatory strategies, particularly those employing nanogenerators to interface with neuronal circuits, have attracted increasing attention for their potential to fine‐tune immune regulation without broadly perturbing systemic signaling. Unlike conventional therapeutic approaches that often impose indiscriminate modulation across interconnected biological networks, nanogenerators enable localized, programmable electrical stimulation of neurons, thereby offering a level of spatiotemporal precision that more closely aligns with the body's intrinsic regulatory architecture.

The nervous system orchestrates immune function through a highly integrated network of chemical, electrical, and mechanical cues, dynamically shaping immune cell behavior and tissue‐specific immune landscapes [2]. By modulating neuronal excitability at the cellular or subcellular level, nanogenerators provide a unique means to intervene within this neuroimmune communication axis, with the potential to restore homeostatic balance under pathological conditions. Such bidirectional crosstalk is essential not only for coordinating effective immune surveillance and resolution of inflammation but also for preserving neuronal integrity and functional stability [3, 4].

Disruption of this finely balanced neuroimmune interface contributes to the pathogenesis of a broad spectrum of disorders, including neurodegenerative diseases such as Alzheimer's disease, autoimmune conditions, and cancer [5, 6, 7]. Consequently, a deeper understanding of the physiological principles governing neuroimmune interactions—and of how nanogenerator‐based neuromodulation can precisely regulate neuronal signaling to re‐establish immune equilibrium—is critical for the rational design of next‐generation, targeted therapeutic strategies.

Adding further complexity, the bidirectional influence between the nervous and immune systems means that neurons can directly modulate immune cell activity, while immune‐derived factors such as cytokines and chemokines can, in turn, reshape neuronal signaling [8]. This dynamic and reciprocal relationship is central to both acute immune responses and chronic disease progression, impacting tissue repair, inflammation resolution, and systemic homeostasis [9]. As such, precisely modulating neuroimmune interactions holds immense therapeutic potential, especially in diseases characterized by immune system hyperactivity (e.g., autoimmune diseases) or hypoactivity (e.g., neurodegeneration) [10].

Recent advances in nanotechnology have introduced groundbreaking approaches to manipulating neuroimmune pathways [11]. Among these, nanogenerators have emerged as a transformative innovation. Initially developed for energy harvesting applications, nanogenerators convert mechanical, thermal, or biological energy into electrical signals at the micro‐ to nanoscale [12]. The realization that bioelectricity is a fundamental mode of communication in both neural and immune systems led researchers to envision nanogenerators not merely as passive energy devices but as active modulators of biological function. Specifically, the ability of nanogenerators to deliver localized, tunable electrical stimuli offered a new method for influencing neuroimmune dynamics with unprecedented precision [13].

The conceptual leap to applying nanogenerators in neuroimmunology is rooted in the intrinsic sensitivity of neurons and immune cells to electrical and mechanical cues [14]. For instance, peripheral nerves such as the vagus nerve are known to regulate systemic immune responses through electrical signaling—a mechanism termed the “inflammatory reflex.” Similarly, immune cells exhibit electrosensitivity that can affect cytokine production, migration, and activation [15]. Recognizing these bioelectrical susceptibilities provided a compelling rationale for developing nanogenerator‐based tools capable of interfacing with neuroimmune circuits, modulating immune responses without the need for systemic drugs or invasive devices [16].

By harnessing endogenous mechanical activities—such as breathing, heartbeat, or even tissue deformation—nanogenerators can generate site‐specific electrical outputs that modulate neuronal excitability and immune cell behavior [17, 18]. This capacity to deliver energy precisely where and when it is needed positions nanogenerators as a novel class of bioelectronic medicines, bridging traditional gaps between the nervous and immune systems.

Nanogenerators thus have a profound impact on neuroimmunology by enabling direct, minimally invasive modulation of neuroimmune interactions. Through localized electrical stimulation, these devices can influence key signaling pathways involved in immune activation, cytokine release, and immune tolerance, offering fine‐tuned control over inflammatory processes [19, 20]. This unique capability opens promising avenues for treating conditions such as autoimmune diseases, chronic inflammation, and neurodegenerative disorders, where precise immunomodulation is critically needed.

**FIGURE 1:**
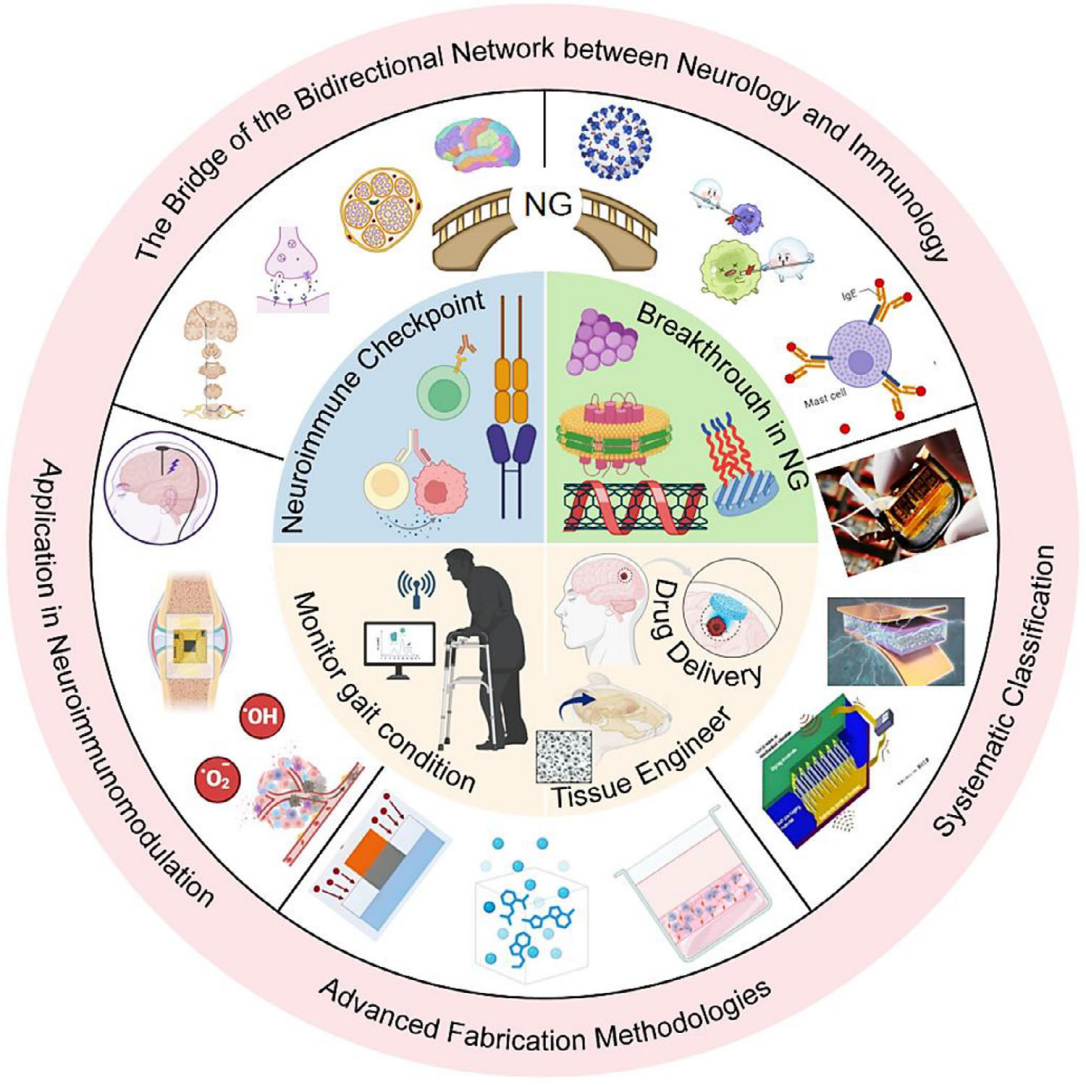
Classification, manufacturing processes of nanogenerators, and their applications in neuroimmune diseases. Created with BioRender 2026. License link: https://BioRender.com/k7tvtey.

This review explores the evolving role of nanogenerators in neuroimmunology, with a particular focus on their potential to regulate immune responses via neural modulation (Figure 1). It begins by elucidating the physiological underpinnings of neuroimmunity, detailing the bidirectional communication that shapes health and disease. The discussion then transitions to the development, synthesis, and systematic classification of nanogenerators, highlighting the technological innovations that have paved the way for their biomedical applications. Furthermore, the review addresses the challenges inherent to applying nanogenerators for neuroimmunomodulation—such as biocompatibility, targeting specificity, and safety—and evaluates their future clinical prospects.

The integration of nanogenerators into neuroimmunology represents a pivotal advancement in precision medicine. As research continues to navigate the intricate interplay between neuronal function and immune balance, these nanoscale devices offer transformative possibilities for achieving localized, adaptive immune regulation. Moving forward, the ability to finely modulate neuroimmune interactions may shift from a conceptual possibility to a clinical practice, establishing a new paradigm for treating complex immune‐mediated diseases. By deepening our understanding of neuroimmune regulation and expanding the technological toolbox available for its modulation, nanogenerators are poised to reshape the future landscape of neuroimmunological therapies.

## 2 Physiological Complexities of Neuroimmune Regulation

Nanogenerators represent a promising frontier in the modulation of neuroimmune interactions, offering novel therapeutic opportunities in diverse pathological contexts. These advanced nanoscale devices are capable of harvesting biomechanical or thermal energy and converting it into electrical signals, thereby enabling precise, non‐invasive stimulation of neural and immune components at the cellular level. The nervous and immune systems, though functionally distinct, are intricately interwoven through dynamic and bidirectional signaling networks mediated by neurotransmitters, neuropeptides, and cytokines [21]. Immune cells express receptors for neuron‐derived mediators, while neurons similarly respond to immune‐derived cues by expressing receptors for cytokines and other immunomodulatory factors [22]. This reciprocal communication forms the basis of neuroimmune crosstalk, underpinning both physiological homeostasis and pathological processes.

**FIGURE 2:**
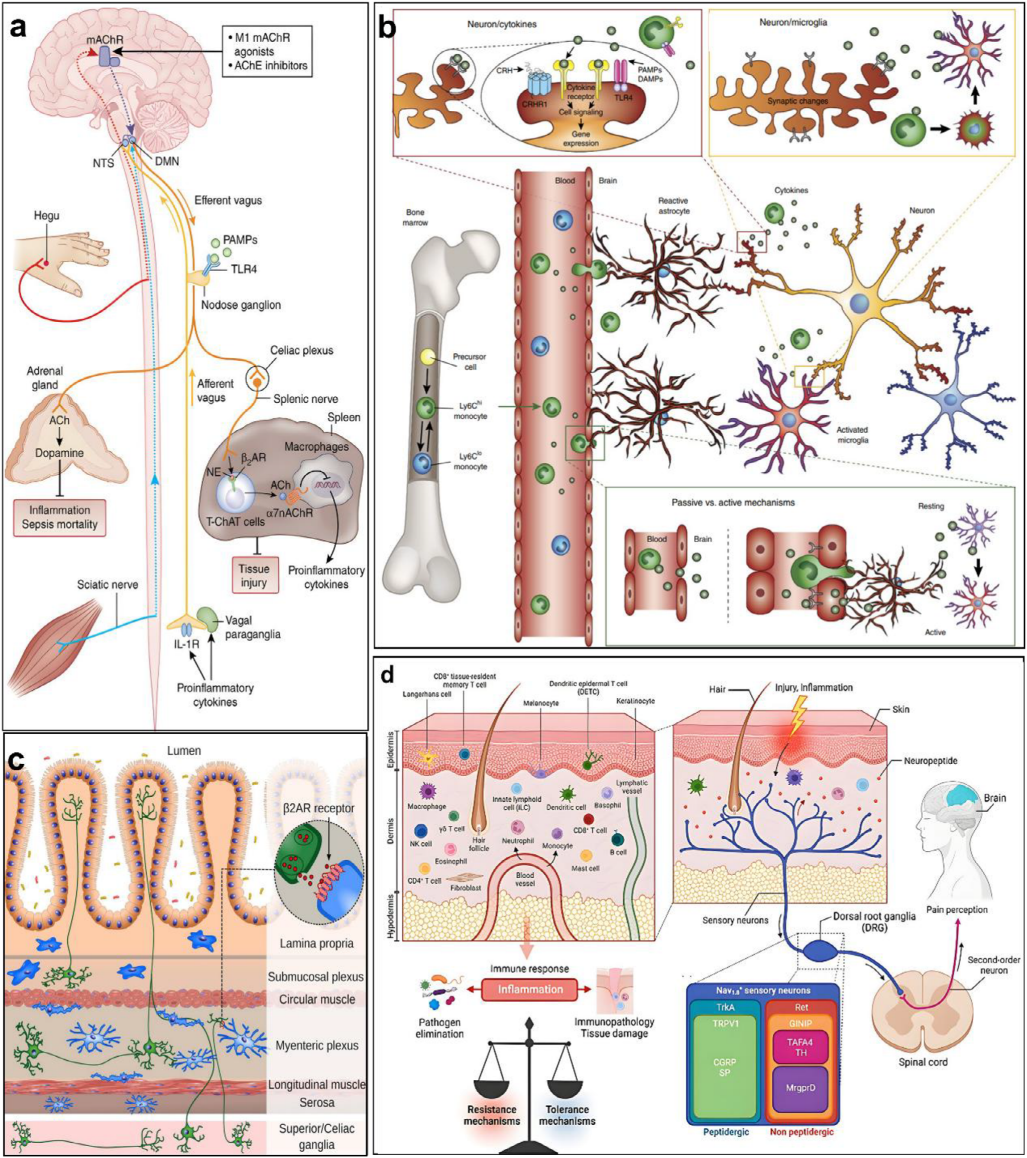
(a) Vagus nerve‐mediated reflex circuitry in immunity and inflammation. Reproduced with permission [24]. Copyright 2017, Springer Nature. (b) The relationship between inflammation and the brain: Chronic stress leads to an increase in circulating monocyte levels, particularly Ly6Chi cells, which are attracted by chemokines to brain regions associated with anxiety and depression. Reproduced with permission [25]. Copyright 2015, Springer Nature. (c) Rapid activation of extrinsic sympathetic neurons innervating the gut muscularis, and norepinephrine signaling to β_2_ adrenergic receptors on the myenteric plexus (MMs). Reproduced with permission [26]. Copyright 2016, Elsevier. (d) Neuroimmune responses in the skin. Reproduced with permission [23]. Copyright 2022, Elsevier.

Figure 2a illustrates one such example: the vagus nerve‐mediated inflammatory reflex circuit [23]. In this reflex arc, afferent fibers of the vagus nerve, situated in the nodose ganglion, are activated in response to circulating cytokines and pathogen‐associated molecular patterns (PAMPs). These signals are transmitted to the nucleus tractus solitarius (NTS), which communicates bidirectionally with the dorsal motor nucleus of the vagus (DMN). Efferent fibers originating from the DMN are subsequently activated, completing the circuit and modulating downstream immune functions.

Chronic stress exemplifies how dysregulated neuroimmune interactions contribute to disease. Prolonged stress elevates levels of circulating inflammatory monocytes, promoting their homing to brain regions associated with anxiety and depression. These monocytes, together with stress‑induced factors, alter synaptic plasticity by interacting with neuronal receptors such as CRHR1 and TLR4, thereby activating microglia to secrete additional cytokines. This establishes a positive feedback loop that recruits more monocytes. Some of the infiltrating monocytes can differentiate into microglia‑like phenotypes. Meanwhile, cytokines enter the brain either through passive diffusion across the stress‑compromised blood‑brain barrier or via receptor‑mediated transport. Increased blood‑brain barrier permeability further facilitates monocyte transendothelial migration, ultimately impairing astrocytic function and sustaining a state of neuroinflammation (see Figure 2b) [24].

Figure 2c highlights the role of neuroimmune crosstalk in shaping tissue‐specific immune programming within the gut [25]. Intestinal macrophages are compartmentalized into functionally distinct subsets: muscularis macrophages (MMs), which adopt tissue‐protective phenotypes, and lamina propria macrophages (LpMs), which display pro‐inflammatory characteristics. Upon luminal bacterial challenge, sympathetic neurons innervating intestinal smooth muscle are rapidly activated, releasing norepinephrine that engages β2‐adrenergic receptors on MMs, further promoting their tissue‐protective programs.

Similarly, neuroimmune communication in the skin orchestrates a complex network that governs inflammation, wound healing, and antimicrobial defense (Figure 2d) [26]. This system involves: (1) neuropeptides such as calcitonin gene‐related peptide (CGRP) and substance P (SP) released from sensory neurons that modulate immune cell function; (2) sympathetic neurotransmitters influencing immune cell polarization through receptor engagement; (3) neurogenic inflammation that facilitates immune recruitment and tissue repair; and (4) microbial metabolites that interface with immune pathways via neural signaling. Dysregulation of this finely tuned network is implicated in various disorders, including postherpetic neuralgia and atopic dermatitis. A deeper understanding of these mechanisms may open avenues for innovative therapies targeting neural regulation in skin pathologies.

Taken together, these examples underscore the central role of neuroimmune crosstalk in maintaining tissue integrity and responding to pathological insults. Nanogenerators, by precisely activating or modulating these pathways, hold immense potential for therapeutic intervention. Their ability to fine‐tune neuroimmune interactions offers promising strategies for alleviating inflammation, regulating CNS disorders, and enhancing antitumor immunity. The integration of nanotechnology with neuroimmunology heralds a transformative shift toward precision medicine, enabling targeted manipulation of disease‐relevant pathways. The following section delves deeper into the molecular architecture and physiological relevance of these neuroimmune circuits, laying the foundation for next‐generation therapeutic strategies.

### 2.1 Homeostatic Neuroimmune Interactions

The neuroimmune system maintains physiological homeostasis by mediating dynamic bidirectional communication between the neural and immune systems. This process is regulated by a complex network of signaling molecules, including neurotransmitters and cytokines, which ensures the stability of immune responses and neural functions while contributing to essential physiological processes such as immune surveillance. Disruption of this equilibrium can trigger neuroinflammation and neurodegenerative diseases, highlighting the therapeutic value of precise modulation of neuroimmune signaling under pathological conditions. Against this backdrop, self‐powered nanogenerators emerge as a powerful tool for the spatiotemporal regulation of neuroimmune interactions, offering opportunities for noninvasive and targeted intervention that align closely with the objectives of precision neuroimmune medicine.

**FIGURE 3:**
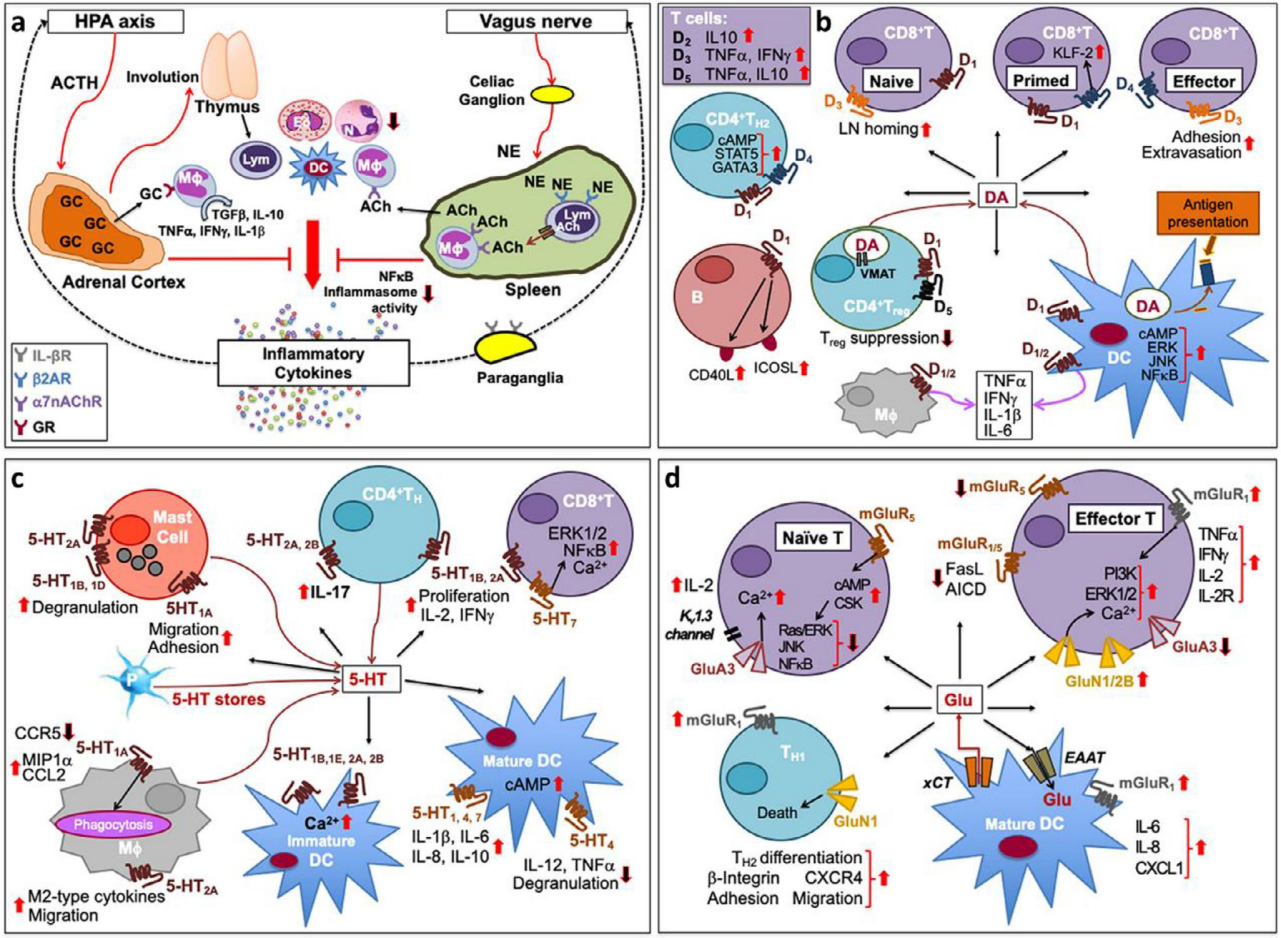
(a) Classical neuroimmune network. (b) Dopamine‐mediated cell‐to‐cell communication among immune cells. (c) Immune network of serotonin‐mediated cell‐to‐cell communication. (d) Glutamate‐mediated cell‐to‐cell communication among immune cells. Critical Neurotransmitters in the Neuroimmune Network. Reproduced with permission [21]. Copyright 2020, Frontiers Media S.A.

Figure 3a depicts a canonical neuroimmune network, characterized by a dynamic feedback loop involving reciprocal signaling between neural and immune components [21]. Key neurotransmitters such as dopamine, serotonin, and glutamate exert immunomodulatory effects by binding to specific receptors expressed on immune cells, either via classical synaptic transmission or through volume diffusion in the extracellular space. These bidirectional pathways maintain immune‐neural balance under healthy conditions and contribute to the pathophysiology of various diseases when dysregulated (see Figure 3b–d). Understanding and manipulating these complex signaling axes represent a key frontier in developing innovative therapeutic strategies for neuroimmune disorders.

#### 2.1.1 Bidirectional Signaling Networks

Neuroimmune interactions are mediated by diverse neural mediators, including neurotransmitters, cytokines, chemokines, and neuropeptides, facilitating bidirectional regulation between neurons and immune functions [27]. At the local level, the central nervous system participates in immune modulation via neuroendocrine mediators, while nerve terminals within immune organs directly communicate with immune cells. Concurrently, cytokines released by immune cells regulate neuronal excitability, synaptic plasticity, and neuroprotective functions, playing a crucial role in neurodevelopment and synaptic remodeling. At the systemic level, long‐range neuroimmune interactions coordinate the body's integrated response to challenges such as infection and injury. The nervous system modulates immune activity, while immune activity in turn influences neural homeostasis and behavior [28]. Dysregulation of this communication axis is closely associated with chronic inflammation, neurodegenerative diseases, psychiatric disorders, and autoimmune diseases, with disruption of this homeostatic balance recognized as a key mechanism underlying these conditions.

A deeper mechanistic understanding of bidirectional neuroimmune signaling not only advances our knowledge of neuroimmune pathophysiology but also provides a foundation for innovative therapeutic strategies. The bidirectional signaling network between neural signals and immune signals is a core mechanism for maintaining organismal homeostasis and regulating disease progression — the two do not act in isolation, but form a dynamic interactive network of “neural regulation of immunity and immune feedback to the nervous system” through shared molecular targets, cellular pathways, and microenvironmental mediators. It coordinates functions under physiological conditions, while it may exacerbate damage due to network imbalance under pathological conditions. Emerging technologies in bioelectronic medicine and nanotechnology offer novel approaches to precisely modulate these interactions, paving the way for targeted interventions in neuroimmune‐related diseases. As this field continues to evolve, integrating interdisciplinary insights will be crucial for developing transformative treatments that harness the full potential of neuroimmune modulation in health and disease.

#### 2.1.2 Neuroimmune Checkpoint Dynamics and Regulatory Mechanisms

**FIGURE 4:**
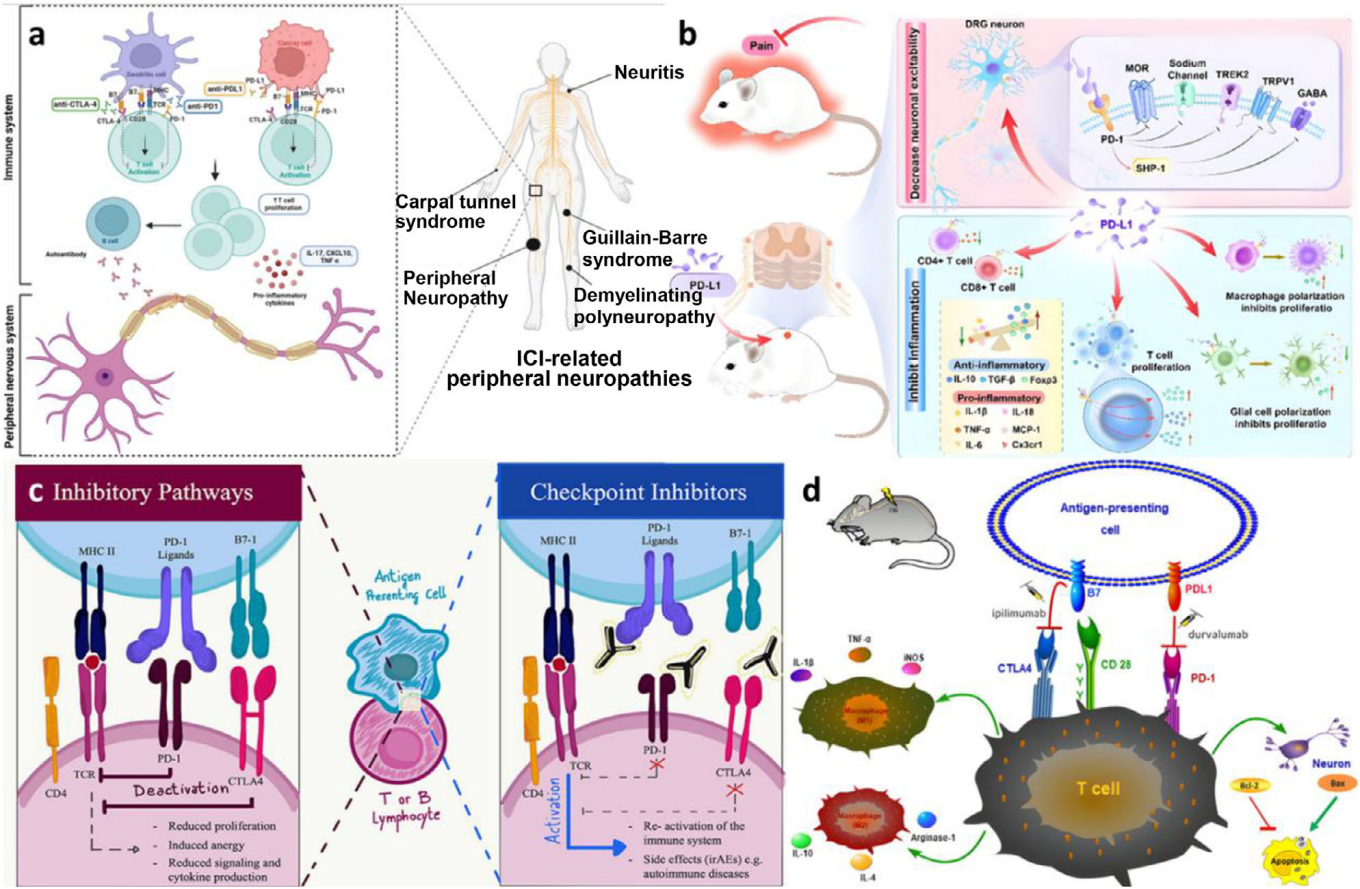
(a) The mechanism of action of ICIs and possible associated neuropathies. Reproduced with permission [33]. Copyright 2023, Frontiers Media S.A. (b) The mechanism overview of the PD‐L1/PD‐1 pathway in pain. Reproduced with permission [34]. Copyright 2024, BioMed Central. (c) Immune checkpoint function during activation or inhibition pathways. Reproduced with permission [35]. Copyright 2022, Frontiers Media S.A. (d) Schematic model for the role of CTLA4 in neuronal cell death following SCI. Reproduced with permission [36]. Copyright 2024, Springer Nature.

Immune checkpoint molecules, originally studied in cancer immunotherapy, have recently been acknowledged for their significant roles in regulating neuroimmune responses [29]. Figure 4a illustrates the mechanism of action of immune checkpoint inhibitors and their potentially associated neuropathies. The core mechanism involves blocking pathways that lift the immune suppression of tumor cells on T cells, thereby activating an anti‐tumor immune response. However, this process may trigger abnormal immune reactions that attack the nervous system. Molecules like PD‐1 and CTLA‐4, known for suppressing peripheral immune responses, are also expressed by CNS‐resident immune cells, including microglia, and play a critical role in maintaining microglial activity and neuronal survival [30, 31]. Loss of checkpoint regulation within the brain can precipitate uncontrolled microglial activation, increased oxidative stress, and excessive cytokine production, thereby exacerbating neuroinflammation and neurodegeneration [32]. This “dark side” of checkpoint inhibition in the CNS highlights the delicate balance between immune activation and protection, underscoring the importance of developing CNS‐specific checkpoint modulators that can preserve neuroprotection while preventing systemic immune suppression.

Neuronal immune checkpoints are crucial for maintaining immune tolerance and protecting neural tissues from excessive inflammation [37]. Key checkpoint molecules such as PD‐1, CTLA‐4, and LAG‐3 regulate immune responses through complex signaling pathways [38]. For instance, the PD‐1/PD‐L1 axis plays a pivotal role in modulating T cell activation, which is critical in neuroinflammatory and neurodegenerative conditions. CTLA‐4 limits T cell proliferation through interactions with CD80/CD86, while LAG‐3 inhibits T cell function via binding to MHC II molecules (see Figure 4b). These checkpoints help regulate immune homeostasis, preventing excessive neuroinflammation while allowing for immune surveillance [39]. However, their dysregulation can lead to pathologies such as neurodegenerative diseases and immune evasion by CNS tumors.

As shown in Figure 4c, the PD‐L1/PD‐1 pathway exerts analgesic effects by regulating macrophages/microglia, T cells, cytokines, and neurons. Specifically, it inhibits the release of pro‐inflammatory cytokines (such as IL‐6 and TNF‐α) from macrophages/microglia and induces their polarization toward an anti‐inflammatory phenotype, reduces T cell infiltration and activation to diminish immune attacks at neuro‐immune synapses, and directly acts on PD‐1 receptors on neurons to modulate ion channel activity—thereby inhibiting nociceptive signal transduction and alleviating neuropathic pain [34]. Figure 4d illustrates the role of CTLA4 in neuronal cell death following spinal cord injury. The schematic shows that CTLA4 contributes to the neuronal death process by regulating T‐cell activation and immune‐inflammatory responses. Possible mechanisms include: abnormal CTLA4 expression triggers excessive T‐cell activation and infiltration into the injured area, leading to the release of cytokines such as IFN‐γ that exacerbate microglial activation and oxidative stress; alternatively, CTLA4 binds to co‐stimulatory molecules on antigen‐presenting cells, inhibiting regulatory T‐cell function and weakening neuroprotective effects, ultimately promoting apoptosis or necrosis of neurons and oligodendrocytes [36].

The activity of neuronal immune checkpoints is dynamically regulated by environmental factors such as inflammation, hypoxia, and the tumor microenvironment [30].​ Pro‑inflammatory signals and oxidative stress can alter checkpoint expression, leading to immunosuppression or exacerbated neuroinflammation. Hypoxia remodels the checkpoint network through the HIF signaling pathway, promoting both immune escape and neuroprotection. Meanwhile, the immunosuppressive and metabolically abnormal tumor microenvironment establishes an immune‑privileged niche, thereby facilitating tumor progression [40]. Elucidating the interaction mechanisms between these factors and immune checkpoints is essential for developing precision therapies that target neuroimmune pathways. Future research should focus on optimizing checkpoint‑targeted strategies that balance neuroprotection with immune activation, thereby advancing treatments for neurodegenerative diseases, tumors, and neuroinflammatory disorders [41].

### 2.2 Pathological Neuroimmune Crosstalk

Pathological neuroimmune crosstalk refers to the dysregulated communication between the nervous system and the immune system, which exacerbates or drives the progression of various neurological diseases [42]. In pathological states such as Alzheimer's disease, Parkinson's disease, multiple sclerosis, and stroke, this balance is disrupted. For instance, in Alzheimer's disease, the accumulation of abnormal proteins like A_β_ and Tau activates microglia, leading to the release of pro‐inflammatory cytokines such as IL‐1β [5, 43]. Similarly, in Parkinson's disease, the misfolded protein α‐synuclein triggers inflammation through TLR_2_ activation [44]. After a stroke, damage‐associated molecular patterns (DAMPs) activate the NLRP_3_ inflammasome, further fueling inflammation [45]. These “danger signals”—abnormal proteins and nucleic acids released due to neuronal damage—are recognized by immune receptors such as TLRs and TREM_2_, initiating inflammatory pathways like NF‐κB and MAPK, which promote a cytokine storm and oxidative stress.

This pathological crosstalk leads to chronic activation of resident immune cells such as microglia and astrocytes, which continuously contribute to neuroinflammation [46, 47]. As a result, neurons experience sustained damage, synaptic loss, and a decline in cognitive functions. Additionally, peripheral immune cells, including T cells and macrophages, infiltrate the CNS, amplifying the inflammatory environment and disrupting the repair mechanisms needed for neural recovery. Cytokines like IL‐6 and TNF‐α secreted by these immune cells can further compromise the blood‐brain barrier (BBB), making it more permeable and exacerbating neuronal injury by inhibiting synaptic plasticity. This creates a vicious cycle of “nerve damage – immune activation – secondary damage,” perpetuating the disease's progression [48, 49]. Understanding the mechanisms of pathological neuroimmune crosstalk is vital for developing targeted therapies that can break this cycle.

#### 2.2.1 Mechanistic Insights into Neuroinflammation

**FIGURE 5:**
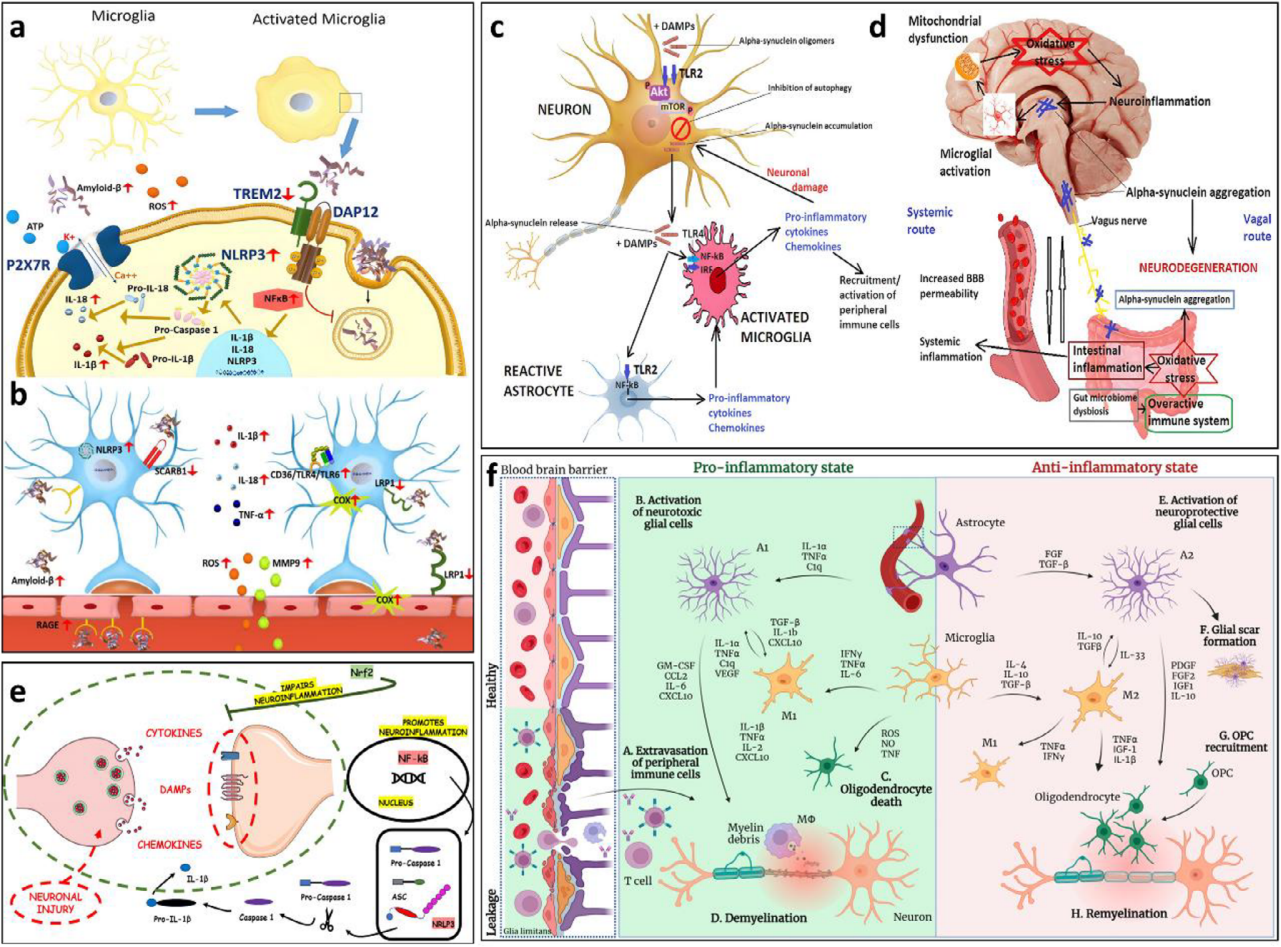
(a) Microglia activation in AD. (b) A representative scheme demonstrates astrocytes’ interaction with the BBB and the neuroinflammation effect mediated by astrocyte activation on the BBB function. Reproduced with permission [52]. Copyright 2022, MDPI. (c) The role of TLR_2_ and TLR_4_ in PD. Oligomeric α‐synuclein or other DAMPs activate neuronal TLR_2_ and inhibit autophagy via the Akt/mTOR pathway. (d) The gut–brain axis in Parkinson's disease. Reproduced with permission [55]. Copyright 2023, MDPI. (e) The pro‐neuroinflammatory potential associated with the activation of NF‐kB and the NLRP_3_ inflammasome lies in their capacity to initiate and intensify inflammatory cascades within the nervous system. Reproduced with permission [46]. Copyright 2024, Frontiers Media S.A. (f) Glial cells in the center of inflammation and neurodegeneration in MS. During early MS, there is a significant compromise of the BBB, through which activated immune cells extravasate from the periphery into the CNS. Reproduced with permission [56]. Copyright 2024, MDPI.

The core mechanisms of neuroinflammation involve the activation of glial cells, such as microglia and astrocytes, the release of inflammatory mediators, the infiltration of peripheral immune cells, and the disruption of the BBB [50]. As shown in Figure 5e, these processes are interconnected through complex signaling networks, including the activation of the NF‐κB pathway and NLRP_3_ inflammasome, which drive chronic inflammation and promote the progression of neurological diseases [51].

In various neurodegenerative conditions, the driving factors and pathological consequences of neuroinflammation are specific to the disease context. For example, in Alzheimer's disease (AD), the A_β_/NLRP_3_ axis activates microglia, triggering a persistent inflammatory response that leads to neuronal damage and cognitive decline (see Figure 5a) [52]. Figure 5b illustrates the interaction between astrocytes and the blood‐brain barrier (BBB), as well as the impact of astrocyte activation on BBB function mediated by neuroinflammation. Under normal conditions, astrocytes maintain the integrity of the BBB tight junctions by secreting neurotrophic factors. Upon activation, they release pro‐inflammatory cytokines such as IL‐1β and TNF‐α, which induce degradation of endothelial tight junction proteins, increasing BBB permeability. This allows peripheral immune cells to infiltrate the central nervous system and exacerbate neuroinflammation, forming a vicious cycle [53]. In multiple sclerosis (MS), glial cells are central to inflammation and neurodegeneration [54]. In the early stage of MS, the blood‐brain barrier (BBB) is significantly compromised, allowing activated immune cells to extravasate from the periphery into the central nervous system (CNS) through the damaged barrier. This triggers the activation of microglia and astrocytes, which release pro‐inflammatory cytokines such as IL‐1β and IFN‐γ, exacerbating local inflammatory responses (see Figure 5f).

In PD, α‐synuclein triggers inflammatory pathways through TLR_2_ activation, leading to neurodegeneration [57]. Each of these pathways represents a unique aspect of neuroinflammation, but they share common features, such as the release of pro‐inflammatory cytokines like TNF‐α and IL‐6, which amplify the inflammatory cascade and disrupt neural function (see Figure 5c). The gut–brain axis mechanism in Parkinson's disease involves metabolites of gut microbiota affecting the central nervous system via the vagus nerve or blood circulation. Abnormal aggregation of α‐synuclein can spread from intestinal neurons to the brain through the vagus nerve, causing damage to dopaminergic neurons in the substantia nigra‐striatum [55]. Meanwhile, gut barrier dysfunction exacerbates the entry of microbial metabolites into the bloodstream, activating microglia and promoting neuroinflammation, thus forming a pathological cycle of gut–brain interaction (see Figure 5d).

The communication pathways involved in neuroinflammation are diverse and include neural and non‐neural mechanisms that mediate immune surveillance and cellular defense in the CNS [58]. One critical pathway is the blood‐brain barrier (BBB), a specialized structure formed by brain microvascular endothelial cells (BMECs), pericytes, and astrocytes that protect the brain from the circulation and regulate the passage of substances into the CNS [59, 60]. The BBB's structural and functional integrity is essential for maintaining brain homeostasis, and its dysfunction is commonly observed in various neurodegenerative diseases [61]. In conditions such as Alzheimer's disease and Parkinson's disease, BBB breakdown results in the uncontrolled passage of toxic molecules, including pro‐inflammatory cytokines and neurotoxic compounds, leading to neuroinflammation, oxidative stress, and neuronal injury [62].

Beyond the BBB, another key communication pathway in neuroinflammation is the gut–brain axis, which integrates the CNS, the gastrointestinal system, and the immune system [63, 64]. Alterations in the gut microbiome have been linked to a wide range of neurological and psychiatric disorders, including irritable bowel syndrome (IBS), obesity, and neurodegenerative diseases. The gut microbiota communicates with the CNS through microbial‐derived molecules such as short‐chain fatty acids (SCFAs), secondary bile acids (2BAs), and tryptophan metabolites [65]. These molecules can activate immune receptors in the gut and even cross the intestinal barrier to enter the bloodstream, where they can influence the brain through the BBB [66].

Immune cells play a central role in the regulation of neuroinflammation and maintaining homeostasis within the CNS. Microglia, astrocytes, and peripheral immune cells such as T cells and macrophages are involved in immune surveillance and defense. Dysregulation of these immune cells can lead to chronic inflammation and contribute to the pathogenesis of autoimmune diseases and neurodegenerative conditions [67]. For example, in multiple sclerosis, immune cells attack the myelin sheath, while in Alzheimer's and Parkinson's diseases, the activation of microglia and other immune cells contributes to neuronal damage. Understanding the complex interplay between immune cells, signaling pathways, and the CNS environment is crucial for developing targeted therapies that can modulate neuroinflammation and protect against neurodegeneration [68, 69].

Neuroinflammation represents a double‐edged sword: it is essential for defending the brain against injury and infection, yet its chronic activation can drive pathology in a range of neurological diseases. Therapeutic strategies aimed at controlling neuroinflammation, preserving BBB integrity, and modulating immune responses offer promising avenues for treating neurodegenerative disorders and other CNS diseases. As research advances, the intricate relationship between the nervous system and immune system continues to provide valuable insights into the mechanisms underlying neuroinflammation and its potential therapeutic targets.

#### 2.2.2 Disease‐Specific Neuroimmune Dysregulation and Pathophysiological Pathways

**FIGURE 6:**
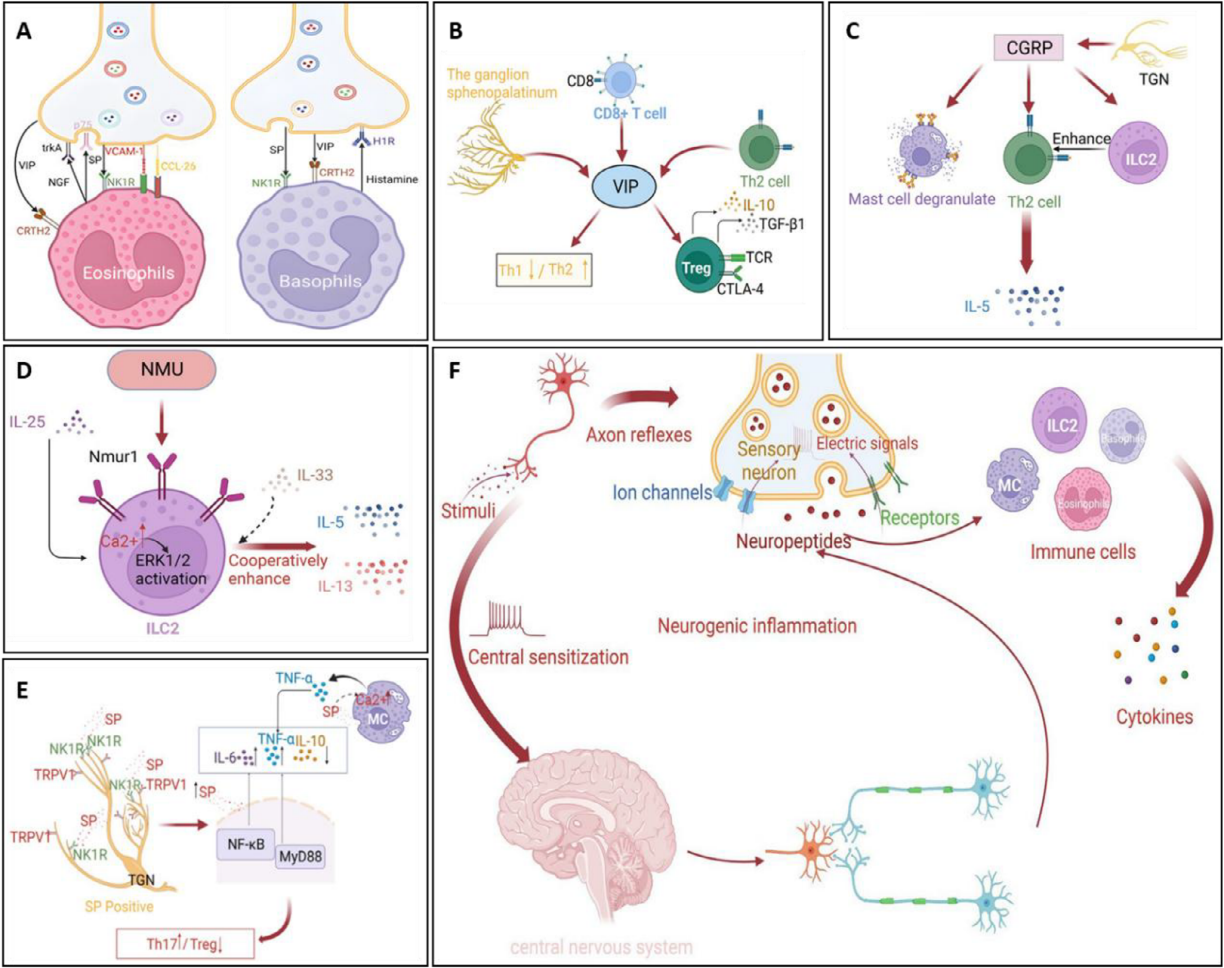
(A) Nerve‐eosinophils unit in neuroimmune communication in allergic rhinitis. (B) Vasoactive intestinal peptide (VIP) in neuroimmune communication of AR. (C) Calcitonin gene‐related peptide (CGRP) in neuroimmune communication of allergic rhinitis. (D) Neuromedin U (NMU) in neuroimmune communication of allergic rhinitis. (E) Substance P (SP) in neuroimmune communication of AR. (F) The process of neurogenic inflammation in AR. Reproduced with permission [71]. Copyright 2023, Frontiers Media S.A.

To better understand the disease‐specific neuroimmune dysregulation and the pathophysiological pathways associated with various conditions, it is important to explore how immune and neural systems interact and contribute to disease onset and progression. Allergic rhinitis (AR) is a prevalent atopic disorder driven by an immunoglobulin E (IgE)‐mediated immune response. Figure 6 illustrates the mechanisms of action of neuropeptide P, vasoactive intestinal peptide, calcitonin gene‐related peptide, and neuromedin U in neuro‐immune communication in allergic rhinitis, as well as the neurogenic processes in which they are involved. The pathogenesis of allergic rhinitis is characterized by the activation of transient receptor potential (TRP) channels, which mediate the influx of cations such as sodium and calcium. This process triggers action potentials and stimulates the release of neuropeptides, including substance P (SP) and calcitonin gene‑related peptide (CGRP). These neuropeptides modulate the differentiation of Th17 and Treg cells, increase vascular permeability and mucus secretion, and simultaneously activate sensory nerves, ultimately leading to symptoms such as itching, sneezing, nasal congestion, and rhinorrhea [70].

Given the involvement of both neurons and immune cells in these symptoms, allergic rhinitis (AR) is classified as a neuroimmune disease. In the nasal mucosa of AR patients, the nociceptive cationic thermo‑responsive channel TRPA_1_ is upregulated, and its pharmacological antagonist HC‑030031 alleviates nasal hyper‑responsiveness and upper respiratory tract inflammation in ovalbumin‑induced AR mouse models. Moreover, the capsaicin receptor TRPV_1_, which responds to thermal, chemical, and mechanical stimuli, is linked to histamine‑dependent pruritus triggered by arachidonic acid metabolites. The close anatomical proximity between sensory nerve fibers expressing TRPV_1_ and mast cells facilitates neuro‑immune interactions. As a TRPV_1_ agonist, capsaicin has been shown to ameliorate cold‑dry‑air‑induced symptoms and hyper‑responsiveness in humans, suggesting that dual targeting of TRPA_1_ and TRPV_1_ may represent a potential therapeutic strategy for AR [72].

Asthma is a chronic inflammatory disorder of the lower airways, characterized by bronchial hyperresponsiveness and airway remodeling [73]. Pulmonary neuroendocrine cells (PNECs), located within the airway epithelium, play a crucial regulatory role in asthma pathogenesis by releasing a diverse array of bioactive substances, including neurotransmitters such as serotonin and γ‐aminobutyric acid (GABA), as well as neuropeptides like substance P, neurokinin A (NKA), vasoactive intestinal peptide (VIP), and calcitonin gene‐related peptide (CGRP).

**FIGURE 7:**
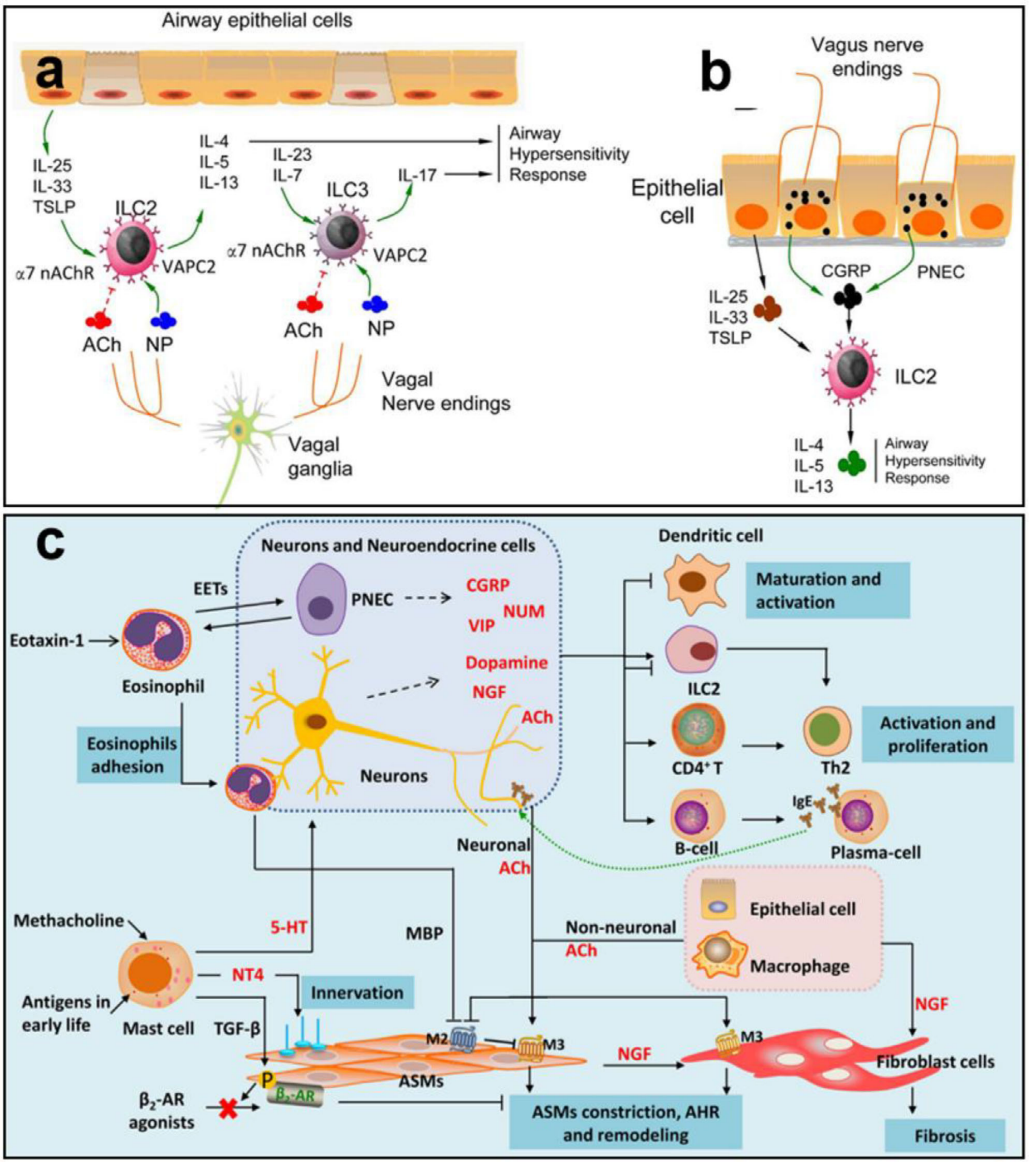
(a) ILC2/3 cells are adjacent to airway epithelial cells and express both neuropeptide receptors and α_7_ nAChR, which can be regulated by neuropeptides and ACh released from vagal nerve endings. (b) The PNECs are innervated by vagal nerve endings and contain CGRP granules. Reproduced with permission [78]. Copyright 2018, Oxford University Press. (c) Neuro‐immune interactions in inflammation and airway remodeling of allergic asthma. Reproduced with permission [79]. Copyright 2022, Frontiers Media S.A.

As illustrated in Figure 7a,b, the distal airways—including the alveolar regions—are innervated by vagal afferent fibers, particularly unmyelinated C‐fibers. These fibers release acetylcholine and neuropeptides that modulate pulmonary immune responses and defense against infection [73, 74]. Acetylcholine, a key parasympathetic neurotransmitter, is not only secreted by nerve terminals but can also be synthesized by various immune cells. Its signaling is primarily mediated through muscarinic acetylcholine receptors (mAChRs), which are G protein‐coupled receptors widely expressed in both neuronal and non‐neuronal tissues [75].

In allergic asthma, neuroimmune interactions are further amplified during airway remodeling. Sensory nerve terminals release neuropeptides such as CGRP and substance P, which activate mast cells, eosinophils, and other effector immune cells [76]. These cells, in turn, release inflammatory mediators—such as histamine and leukotrienes—that exacerbate bronchial hyperresponsiveness [77]. This neuroimmune feedback loop contributes to key pathological features of asthma, including airway smooth muscle hyperplasia, basement membrane thickening, and mucus overproduction (Figure 7c). Collectively, these events perpetuate chronic inflammation and bronchial remodeling, underscoring the critical role of neuroimmune crosstalk in asthma progression and severity.

The skin serves as a dynamic barrier organ, composed of a complex network of skin‐resident immune cells and sensory neurons that collaboratively maintain immune homeostasis and respond to environmental stimuli. Atopic dermatitis (AD) is a common chronic inflammatory skin disorder characterized by a persistent itch–scratch cycle that significantly impairs quality of life [80]. In AD, damage to epidermal keratinocytes initiates the release of inflammatory mediators that activate type 2 immune responses and pruritogenic cytokines [81]. These cytokines bind to receptors on pruriceptive sensory neurons, triggering intense itch and the compulsion to scratch.

A central feature of AD pathophysiology is the neuroimmune interaction that amplifies itch perception. Neuropeptides such as substance P and calcitonin gene‐related peptide (CGRP), along with pruritogenic cytokines such as interleukin‐31 (IL‐31), are released from sensory nerve terminals and immune cells. These mediators engage specific receptors on neurons and immune cells, promoting mast cell degranulation, dendritic cell recruitment, and T‐helper 2 (Th2) cell polarization, thereby intensifying the inflammatory and pruritic response [82].

**FIGURE 8:**
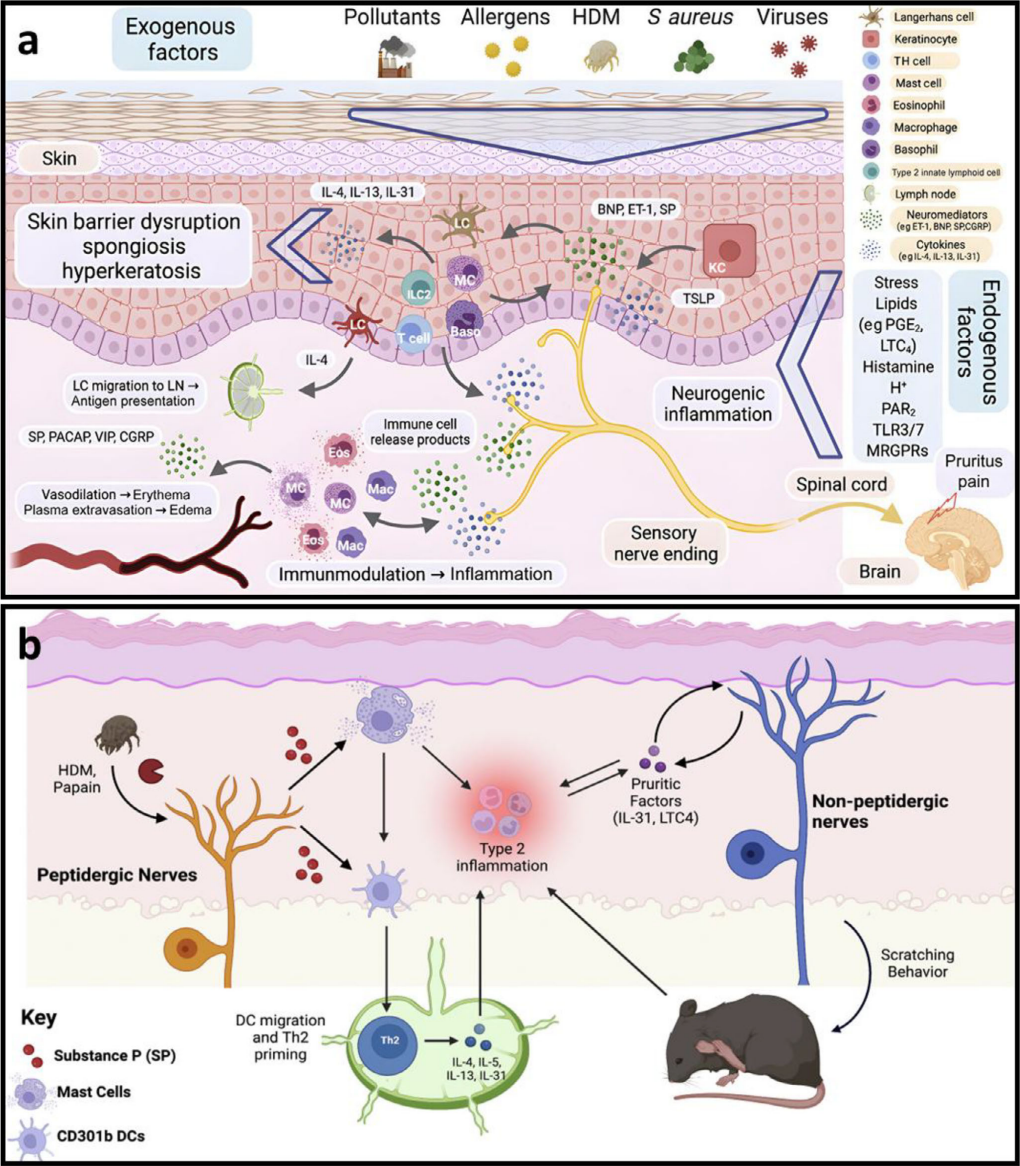
(a) Connection of neuroinflammation and itch or pain in atopic dermatitis. Reproduced with permission [83]. Copyright 2022, Elsevier. (b) Schematic of neuroimmune function in atopic dermatitis. Reproduced with permission [81]. Copyright 2023, Elsevier.

Figure 8a illustrates the role of neuroinflammation in mediating pruritus and pain in AD. Neuroinflammatory processes sensitize cutaneous sensory neurons, which subsequently release neuropeptides such as substance P that enhance vascular permeability and facilitate immune cell infiltration. In parallel, immune cells—including mast cells and basophils—secrete itch‐inducing mediators like histamine and IL‐31, which directly stimulate nerve endings, reinforcing a neuroimmune positive feedback loop that underlies chronic itch and neuronal sensitization [83]. In certain cases, prolonged inflammation and mechanical injury from persistent scratching may result in secondary neural damage, giving rise to neuropathic pain or hyperalgesia, as depicted in Figure 8b. This phenomenon highlights the dual role of neuroimmune crosstalk in mediating both pruritus and pain, offering critical insights into potential therapeutic targets for AD management [81].

The adult gastrointestinal tract harbors the body's largest reservoir of immune cells and is innervated by an extensive neural network containing as many neurons as the spinal cord—earning it the designation of the “second brain” [84]. This intrinsic network, known as the enteric nervous system (ENS), is a component of the autonomic nervous system composed of enteric neurons and glial cells. Beyond its classical role in regulating gastrointestinal motility, the ENS plays a critical role in maintaining intestinal homeostasis by orchestrating neuroimmune interactions within the gut microenvironment.

**FIGURE 9:**
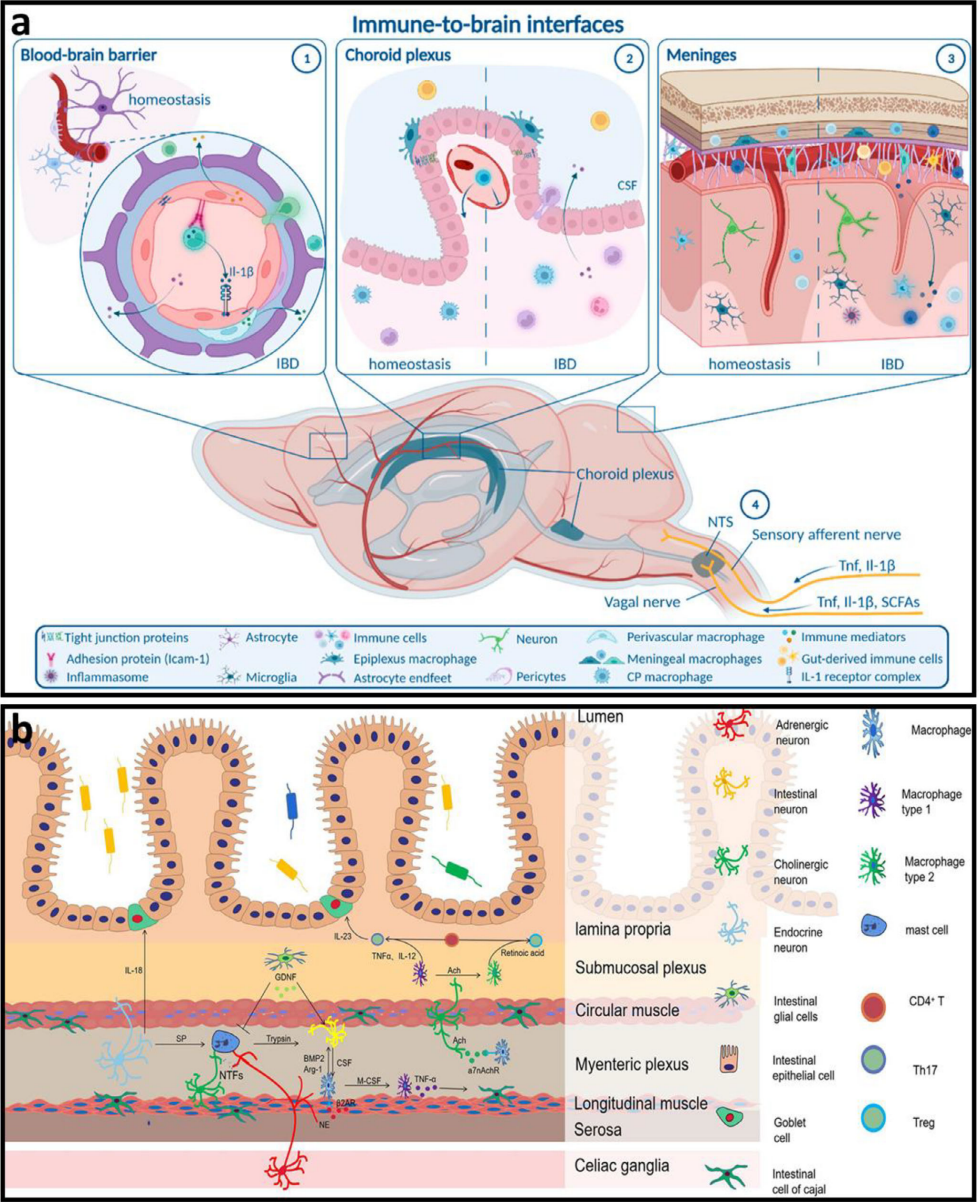
(a) Alterations at immune‐to‐brain interfaces during IBD. Reproduced with permission [85]. Copyright 2022, MDPI. (b) The mechanism of neuroimmune regulation in HAEC. Reproduced with permission [87]. Copyright 2023, Frontiers Media S.A.

In inflammatory bowel disease (IBD), dysregulation of the immune–brain interface has been increasingly recognized. This includes downregulation of tight junction proteins in the blood–brain barrier (BBB) and enhanced endothelial permeability, which facilitate the entry of proinflammatory cytokines and immune cells into the central nervous system (CNS) [85]. Additionally, upregulation of endothelial adhesion molecules promotes immune cell transmigration, while activated endothelial cells and perivascular macrophages secrete mediators that influence neuronal function (Figure 9a). Within the meninges, gut‐derived immune cells infiltrate and activate resident immune cells, including the NLRP_3_ inflammasome, contributing to neuroinflammation.

Within the gut, adrenergic signaling plays a protective role in neuroimmune regulation [86]. Activation of β_2_‐adrenergic receptors (β_2_AR) on muscularis macrophages by norepinephrine (NE) induces the production of neuroprotective polyamines, which are essential for the repair of intestinal neurons. Experimental studies have shown that wild‐type mice infected with attenuated Salmonella exhibit persistent gastrointestinal dysmotility and enteric neuronal damage for up to four months. Notably, deficits in NE signaling exacerbate neuronal loss and inflammation [87].

Figure 9b illustrates the neuroimmune crosstalk involved in Hirschsprung‐associated enterocolitis (HAEC). Macrophages stimulate intestinal neurons via bone morphogenetic protein 2 (BMP_2_) to regulate gut motility, while enteric neurons express colony‐stimulating factor 1 (CSF1) to promote macrophage development and proliferation. Adrenergic nerve fibers further modulate muscularis macrophages through β_2_AR signaling to support neuronal survival. In parallel, cholinergic nerve fibers promote the polarization of macrophages from a proinflammatory M1 phenotype to an anti‐inflammatory M2 phenotype via activation of α_7_ nicotinic acetylcholine receptors (α_7_nAChRs), thereby attenuating local inflammation and enhancing the Treg/Th17 ratio within the intestinal mucosa [87].

Acute kidney injury (AKI) is a clinical syndrome characterized by a rapid rise in serum creatinine and a decline in urine output, often accompanied by robust inflammatory responses that engage and modulate immune circuits. This inflammatory cascade triggers neurological stress, apoptosis, hormonal imbalances, and pain, collectively exacerbating disease progression [88].The kidneys function as critical sensory organs, richly innervated with afferent nerves and mechanosensitive receptors. Renal sympathetic nerves regulate key physiological processes, including water and sodium balance, renin secretion, and vascular resistance [89].

The development of pain in AKI involves complex neuroimmune interactions [90]. Damage‐associated molecular patterns (DAMPs) released from injured renal tissue activate resident immune cells (e.g., macrophages, neutrophils), which in turn secrete pro‐inflammatory cytokines such as IL‐1β that sensitize and damage local sensory nerve endings [91]. Mast cells release histamine and nerve growth factor (NGF), further amplifying nociceptive signaling. Sensory neurons respond by releasing calcitonin gene‐related peptide (CGRP) and substance P, enhancing immune cell activity and forming a positive feedback loop that sustains inflammation and pain [92].

**FIGURE 10:**
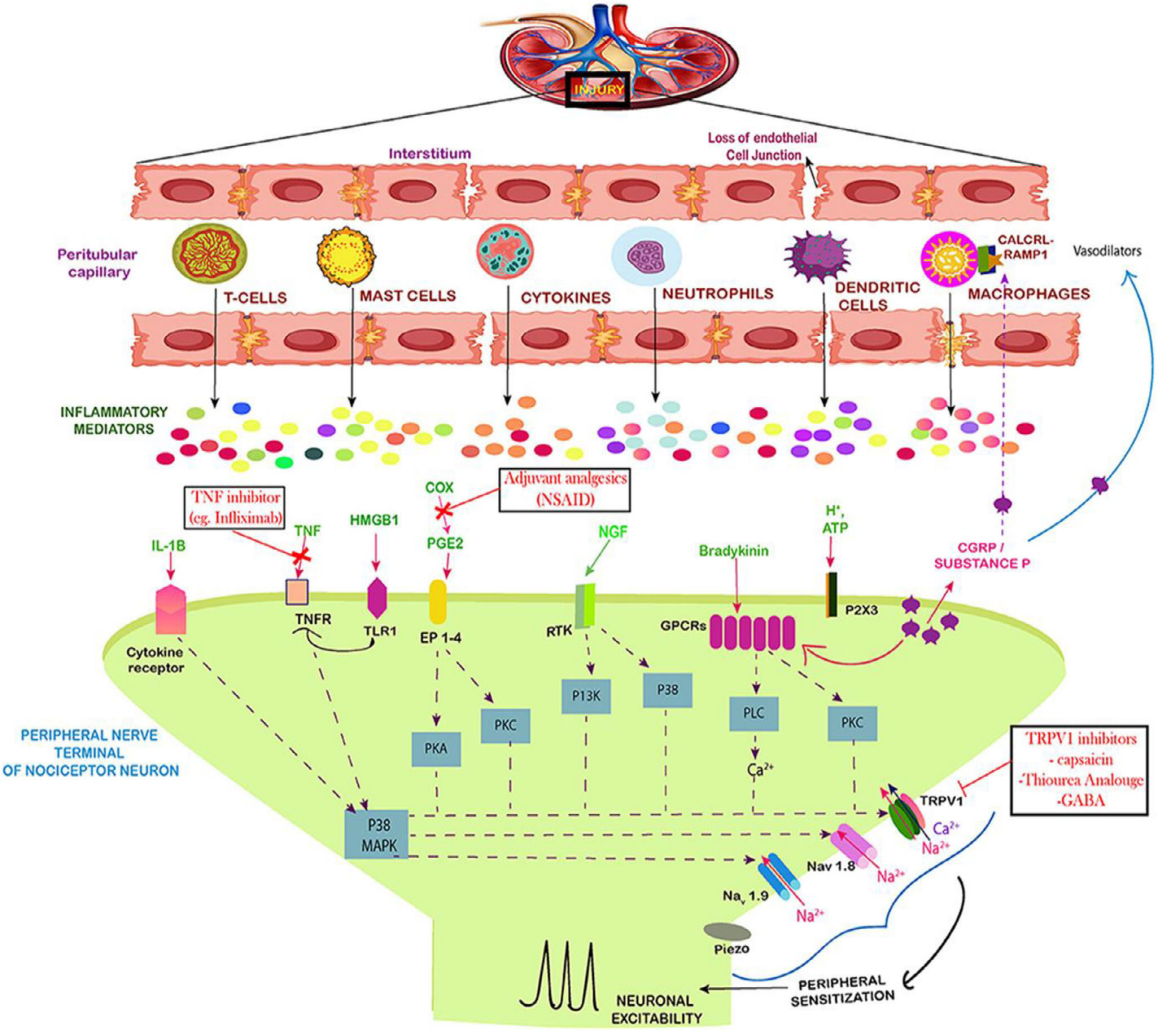
Neuro‐immune interaction contributing to the establishment of pain during AKI. Reproduced with permission [93]. Copyright 2020, Frontiers Media S.A.

Furthermore, immune mediators like prostaglandin E2 (PGE2) and bradykinin further sensitize peripheral nerves. At the central level, neurons in the spinal dorsal horn become hyperresponsive to inflammatory inputs, and functional reorganization of pain‐related brain regions amplifies pain perception. The transition from acute to chronic pain involves dysregulation of critical pathways, such as the NGF/TrkA axis, purinergic signaling, and the cholinergic anti‑inflammatory reflex. Importantly, pain severity is correlated with systemic inflammatory markers and linked to an increased risk of chronic kidney disease and persistent neuropathic pain [93]. Figure 10 illustrates these multidimensional neuroimmune mechanisms. Targeting peripheral sensitization, neuroimmune crosstalk, and central pain pathways may offer novel therapeutic strategies to alleviate pain and limit long‐term sequelae in AKI.

The neuroimmune system also plays a significant role in cerebellar movement disorders, such as ataxia, which presents with symptoms including static ataxia, dysarthria, and nystagmus. The cerebellum, rich in antigens, is particularly vulnerable to immune attacks [94]. Ion channels (e.g., potassium channels), secreted neural proteins (e.g., LGI1), adhesion molecules (e.g., IgLON5), and synaptic proteins (e.g., GluRδ) are among the autoimmunity targets that contribute to cerebellar ataxia [95]. In vivo studies demonstrate that local administration of IL‐1β to Purkinje neurons increases their firing rate and induces ataxia. Chemically activating microglia in the cerebellum similarly enhances Purkinje neuron firing and leads to ataxia. TGFβ1, a neuroimmune molecule involved in cerebellar development, regulates the expression of potassium channels in granule neurons, thereby influencing their electrical activity, growth, and maturation. Autoimmune responses targeting neuroglia, such as autoimmune GFAP astrocytopathy, can also trigger T‐cell‑mediated inflammation in the brainstem, resulting in cerebellar ataxia [96].

Multiple sclerosis (MS) is a chronic autoimmune disorder of the central nervous system (CNS), characterized by immune‐mediated demyelination and impaired remyelination. The pathogenesis of MS involves aberrant immune activation, glial cell dysfunction, and a disrupted CNS microenvironment [97]. Both infiltrating peripheral immune cells and resident glial cells contribute to progressive neurodegeneration [98].

**FIGURE 11:**
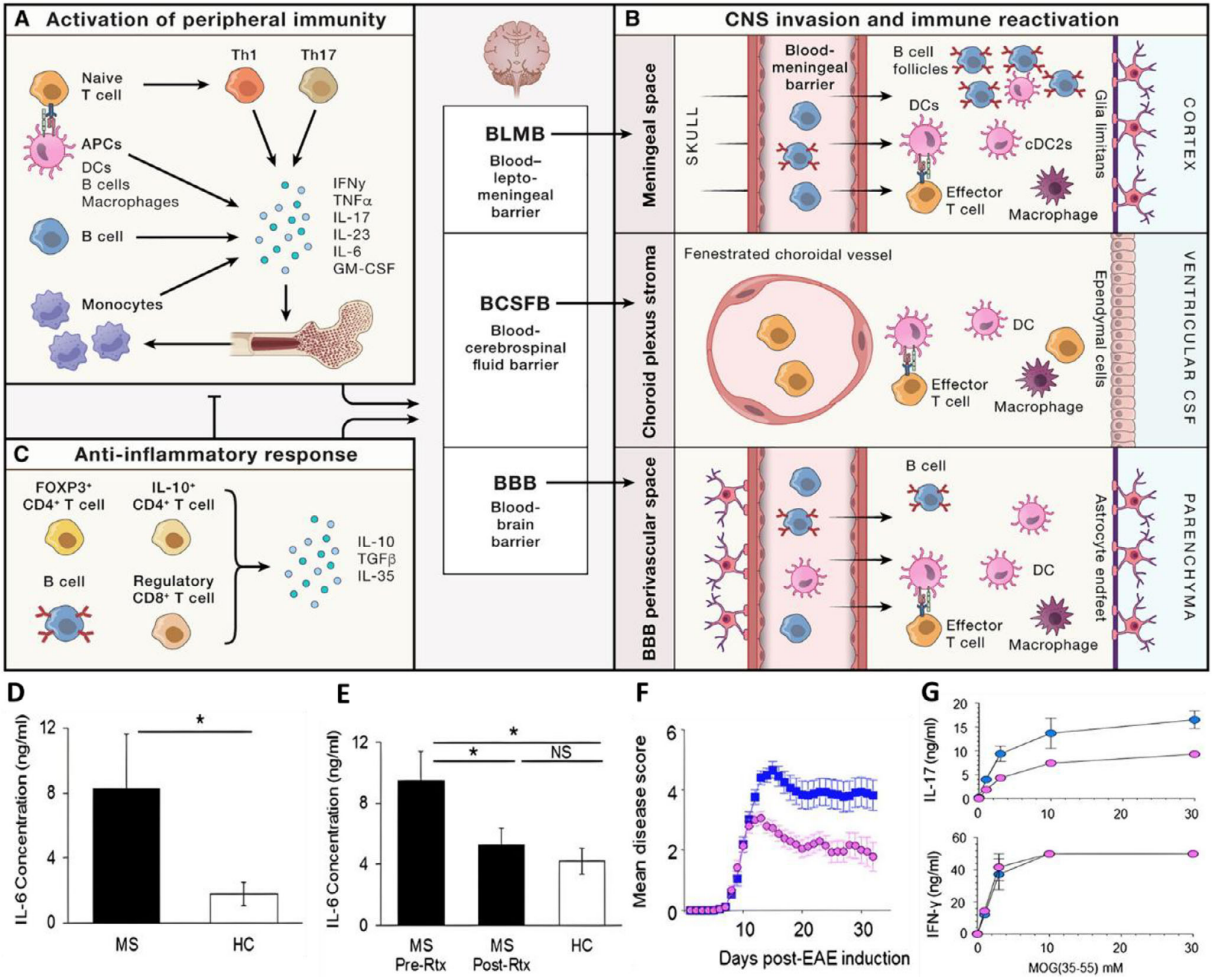
(A) APCs (DCs, macrophages, B cells) and T cells first interact in the periphery, leading to activation and dysregulation of peripheral immunity and the secretion of inflammatory molecules. (B) Peripheral immune cells invade the CNS through the BLMB, BCSFB, and BBB where they can interact with local APC subsets. (C) Impaired Treg suppressive activity contributes to MS pathogenesis. Reproduced with permission [101]. Copyright 2023, Elsevier. (D) IL‐6 production by B cells isolated from patients with multiple sclerosis (MS) is increased compared with healthy controls (HC) after in vitro stimulation. *p<0.05. (E) IL‐6 production from B cells from patients with MS before and after rituximab treatment. (F) Mice with a B‐cell IL‐6 deficiency (B‐IL‐6^−/−^) develop an attenuated form of experimental autoimmune encephalomyelitis (EAE), implying that B cells drive disease exacerbation through the production of IL‐6. (G) In the EAE model, IL‐17 and interferon (IFN)‐γ secretion by CD4 splenic T cells from B‐WT (blue circles) and B‐IL‐6^−/−^ mice (pink circles) shows impaired T helper 17 cell responses. Reproduced with permission [100]. Copyright 2015, Springer Nature.

Among the immune players, B cells are particularly critical in MS progression. They produce autoantibodies, present antigens, and modulate T‐cell responses, ultimately fueling neuroinflammatory cascades and lesion formation (see Figure 11A–C). Autoreactive T cells can breach the blood‐brain barrier (BBB), promoting the formation of characteristic demyelinated plaques and perpetuating local immune activation [99].

As shown in Figures 11D–G, B cells isolated from MS patients exhibit increased interleukin‐6 (IL‐6) production upon in vitro stimulation compared to those from healthy controls. This finding highlights a disease‐specific, pro‐inflammatory reprogramming of B cells in MS. IL‐6, a pleiotropic cytokine, exacerbates disease progression by promoting BBB disruption, activating astrocytes and microglia, and enhancing Th17 cell differentiation—all of which contribute to CNS demyelination and axonal degeneration [100]. These insights suggest that targeting B cells and their IL‐6‐mediated effector functions may represent a promising therapeutic strategy for halting or reversing MS‐associated neuroinflammation and neurodegeneration.

In Alzheimer's disease (AD), chronic neuroinflammation is driven by the synergistic effects of amyloid‐beta (A_β_) and Tau proteins. A_β_ oligomers activate microglial receptors like TLR_4_ and CD36, triggering the NLRP_3_ inflammasome and releasing pro‐inflammatory cytokines such as IL‐1β and IL‐18 [102]. Abnormal phosphorylation of Tau activates the Syk kinase pathway in microglia, leading to the secretion of pro‐inflammatory factors and amplifying the inflammatory response. This creates a positive feedback loop where IL‐1β exacerbates Tau phosphorylation, while Tau promotes A_β_ production, leading to a cycle of neurodegeneration [43]. Chronic inflammation impairs the phagocytic function of microglia, resulting in Aβ deposition and the spread of Tau pathology. Strategies targeting NLRP_3_, TREM_2_, or inhibiting Tau phosphorylation are under investigation to break this vicious cycle.

PD is characterized by neuroinflammation linked to α‐synuclein aggregation. Pathological α‐synuclein oligomers activate TLR_2_ on microglia, initiating downstream signaling that triggers the release of pro‐inflammatory cytokines and reactive oxygen species (ROS). ROS damage dopaminergic neurons by oxidizing mitochondrial DNA and lipids, promoting ferroptosis. IL‐1β exacerbates α‐synuclein aggregation, creating a positive feedback loop. Chronic microglial activation leads to autophagic dysfunction, iron metabolism imbalance, and further oxidative stress. Astrocytes release complement C1q and glutamate, contributing to excitotoxicity and synaptic damage. Strategies targeting TLR_2_/NLRP_3_, α‐synuclein antibodies, or iron chelation are being explored to disrupt this inflammatory axis and offer new therapeutic options for PD [103, 104].

This section systematically elaborates on the pathophysiological pathways of a spectrum of neuroimmune diseases, revealing a shared core pathological feature across disorders affecting the mucosae, skin, intestines, kidneys, brain, and other organs: dynamic dysregulation of the neuro‐immune axis [105, 106]. Although these diseases vary substantially in their initial target organs and clinical manifestations, their pathophysiological mechanisms are rooted in genetically or environmentally triggered aberrant immune responses—with interplay between local and systemic components—accompanied by neuronal sensitization, abnormal neuropeptide release, and neurogenic inflammation [107]. This dysregulation is not a unidirectional process of “immune system affecting the nervous system” or vice versa, but rather a sophisticated positive feedback loop. In this loop, cytokines, chemokines, and damage‐associated molecular patterns (DAMPs) interact reciprocally with neurotransmitters and neuropeptides, collectively driving chronic tissue inflammation, barrier dysfunction, pathological remodeling, and organ impairment.

## 3 Nanogenerator Technology: Fundamentals and Evolution

Nanogenerator technology, first introduced in 2006, leverages the piezoelectric, pyroelectric, and triboelectric effects of nanomaterials to convert environmental mechanical and thermal energy into electrical energy. Through the design of intricate micro‐nano structures, this technology has found applications in self‐powered sensors, wearable devices, and biomedicine [108]. As nanogenerator technology continues to evolve, its potential to transform sectors such as 5G, artificial intelligence, and the Internet of Things becomes more apparent. The deep integration of these technologies promises to propel nanogenerators into new frontiers, offering innovative solutions for the future of sustainable development [109]. In particular, their ability to manipulate neurons presents a unique opportunity to explore immune equilibrium, balancing energy generation with neural modulation for advanced therapeutic applications.

### 3.1 Historical Development and Technological Evolution

The development of nanogenerator technology originated from early research on the piezoelectric, pyroelectric, and triboelectric effects of nanomaterials, aiming to convert mechanical and thermal energy into electrical power. Initially applied primarily in energy harvesting and self‐powered devices (e.g., sensors and wearable technologies) [110], the technology has since expanded into biomedical fields such as medical monitoring and therapeutic devices, driven by significant improvements in energy density, efficiency, and stability. In recent years, the use of nanogenerators to modulate neuronal activity has emerged as a growing research direction. By generating electrical signals in response to environmental stimuli, this approach provides novel pathways for treating neurodegenerative diseases, modulating immune responses, and developing self‐powered neuroprosthetics [111]. As the technology continues to advance, the integration of nanogenerators with neuromodulation is expected to drive innovation in advanced biomedical therapies, opening a new era of energy‐driven neural treatments and immune regulation.

#### 3.1.1 Evolution of Nanogenerators: From Concept to Application

**FIGURE 12:**
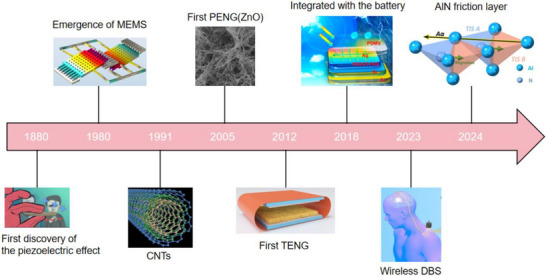
Development process of nanogenerator.

As illustrated in Figure 12, the development of the world's first piezoelectric nanogenerator (PENG) was preceded by over a century of conceptual and technological evolution. The piezoelectric effect was first discovered in 1880 by French physicists Pierre Curie and Jacques Curie, who observed that certain crystals could generate an electric charge in response to mechanical stress [112]. By the 1940s, piezoelectric materials such as quartz and barium titanate found applications in military technologies, including sonar and sensors [113].

The 1980s marked the dawn of a new era with the emergence of Micro‑Electro‑Mechanical Systems (MEMS) technology [114]. In 1991, Japanese physicist Sumio Iijima discovered carbon nanotubes (CNTs) [115]. In 2005, Zhong Lin Wang and his team developed a theoretical model describing the piezoelectric behavior of ZnO nanowires [116]. This led to the birth of the first piezoelectric nanogenerator (PENG), establishing a solid theoretical framework for the emerging fields of piezotronics and piezo‐phototronics and opening a new pathway for powering nanodevices.

In 2012, Zhong Lin Wang's team further advanced the field by inventing the first triboelectric nanogenerator (TENG), based on the triboelectric effect and electrostatic induction [117, 118]. Significant biomedical applications soon followed. In 2014, researchers developed an implantable piezoelectric nanogenerator [119]. In 2018, researchers developed an implantable self‐powered cardiac pacemaker, wherein a flexible thin‐film nanogenerator attached to the heart harvested mechanical energy from heartbeats to regulate cardiac function [120]. In 2013, the research teams utilized TENG technology to fabricate a smart insole based on a polydimethylsiloxane (PDMS) film and a polyethylene terephthalate (PET) film [121]. In 2016, researchers combined TENG and PENG technologies to simultaneously capture low‐frequency human motion and high‐frequency mechanical vibrations [122]. In 2017, the researchers proposed a multifunctional hybrid power device for harvesting blue energy [123]. In 2022, the research team developed a hybrid nanogenerator incorporating photovoltaic, TENG, and thermoelectric generator (TEG) modules, achieving continuous energy collection across various environmental conditions [124].

Nanogenerators have also demonstrated profound potential in specialized applications. In 2023, the research team proposed a novel polydimethylsiloxane elastomer with conjugated benzene rings for preparing heat‐resistant and flame‐retardant triboelectric nanogenerators (TENGs) [125]. In 2024, some researchers have prepared triboelectric nanogenerators made of hydrogels for extreme environments. They have rapidly prepared tough, antifreeze, and conductive hydrogels ([SL‐Fe^3^^+^/P]Li) [126].

This technology not only heralds the arrival of the “battery‐free era” but also redefines energy conversion theory, catalyzes the green energy revolution, and carries profound societal significance in medical accessibility, energy security, and space exploration [127]. Looking ahead, nanogenerators are poised to evolve toward ultra‐high‐efficiency materials, bio‐mechanical integration systems, and the intelligent energy internet, offering robust technological support for sustainable human development and deep‐space exploration.

#### 3.1.2 Breakthroughs in Energy Conversion Efficiency

The breakthroughs in the energy conversion efficiency of nanogenerators are mainly attributed to the development of high‐performance materials, the optimization of structural design, the application of advanced manufacturing processes, the improvement of energy management systems, and the introduction of multi‐physical field coupling technology. These breakthroughs have laid a solid foundation for the practical applications of nanogenerators in fields such as healthcare, the Internet of Things, and environmental monitoring, and have promoted the development of self‐powered systems and wearable devices [128]. In the future, with further technological innovation, nanogenerators are expected to achieve higher energy conversion efficiency and make greater contributions to the utilization of sustainable energy and the development of green technology.

First, the development of high‐performance materials has significantly advanced the field. Materials with higher piezoelectric coefficients, such as barium titanate (BTO), bismuth ferrite (BFO), sodium potassium niobate (KNN), and lead zirconate titanate (PZT), have been extensively studied. Through surface modification and composite material design, the charge density and stability of piezoelectric and triboelectric materials have been substantially improved [129]. Researchers have developed a triboelectric nanogenerator based on nanostructured silicon, which generates electricity by utilizing the charge effects produced when water flows through nanoscale channels. The energy conversion efficiency of this type of triboelectric nanogenerator reaches approximately 9%, representing the highest reported level among similar systems to date [130]. In addition, researchers have developed a hybrid triboelectric layer composed of a polyimide (PI) matrix embedded with carbon nanotube@barium titanate (CNT@BTO) nanoparticles. The enhanced output performance arises from the synergistic coupling of the piezoelectric effect, triboelectric effect, and the integration of microelectrode functionalities [131].

Secondly, the optimization of structural design has played a crucial role in improving nanogenerator performance. Structures such as nanowires, nanopillars, and nanofilms can significantly increase the surface area of materials, thereby enhancing energy conversion efficiency. Inspired by biological microstructures—such as the setae on gecko soles or the intricate textures of plant leaves—researchers have designed hierarchical micro‐nano structures that expand the effective contact area and boost charge capture capabilities, ultimately improving power generation efficiency. Researchers optimized the solid triboelectric layer by adopting a lotus‐leaf‐mimetic surface microstructure design, and constructed the MC‐TENG sensor by combining surface alkalization treatment with liquid metal electrodes. The bionic microstructure not only enhances the surface charge density and roughness, but also improves the wettability of the solid‐liquid interface, enabling the device's power density to exceed 33.54 W/m^2^, which is close to the level of solid‐solid TENGs [132].

Thirdly, advanced manufacturing processes have been introduced to enhance material properties and device performance. For example, a nano‐fiber embedded ZnO composite nanofilm was fabricated by combining electrospinning with traditional direct current (DC) sputtering, leading to improvements in both output efficiency and device lifespan [133]. Researchers fabricated laser‐induced graphene (LIG) patterned electrodes by direct laser engraving on the surface of polyimide (PI). Subsequently, a ZnO piezoelectric layer was loaded via electrochemical deposition to form a triboelectric‐piezoelectric hybrid device. This process avoids the complex procedures of traditional photolithography, improving the device fabrication efficiency by 50%. Meanwhile, the 3D micro‐nano structure enhances the interfacial triboelectric effect, increasing the output power density by more than 2 times compared with the planar structure [134].

Finally, significant advancements have been made in energy management systems to better harness the energy produced by nanogenerators. For example, the development of the Pulsed‐TENG introduced a synchronously triggered mechanical switch at the output end of the TENG, effectively reducing the equivalent output impedance to zero. This design maximizes the harvested energy, minimizes losses during storage, and makes the output independent of load resistance [135]. Researchers have developed a TENG power supply system that integrates energy storage and voltage stabilization functions. Through systematic circuit analysis, it solves the problem of electrical energy regulation for irregular mechanical energy. Under complex excitations such as human movement and environmental vibration, the system can still provide a stable DC output for electronic devices, increasing the energy utilization efficiency by more than 30% compared with traditional Buck converters [136].

Additionally, porous and ultrathin ZnP nanosheets with pseudocapacitive characteristics, combined with laser‐written graphene composites, were used to fabricate an interdigital‐structure micro‐supercapacitor array with an island‐bridge configuration [137]. By efficiently integrating two electrode materials with distinct energy storage mechanisms, the system significantly enhanced energy density without compromising power density or cycle life.

### 3.2 Systematic Classification of Nanogenerators

**FIGURE 13:**
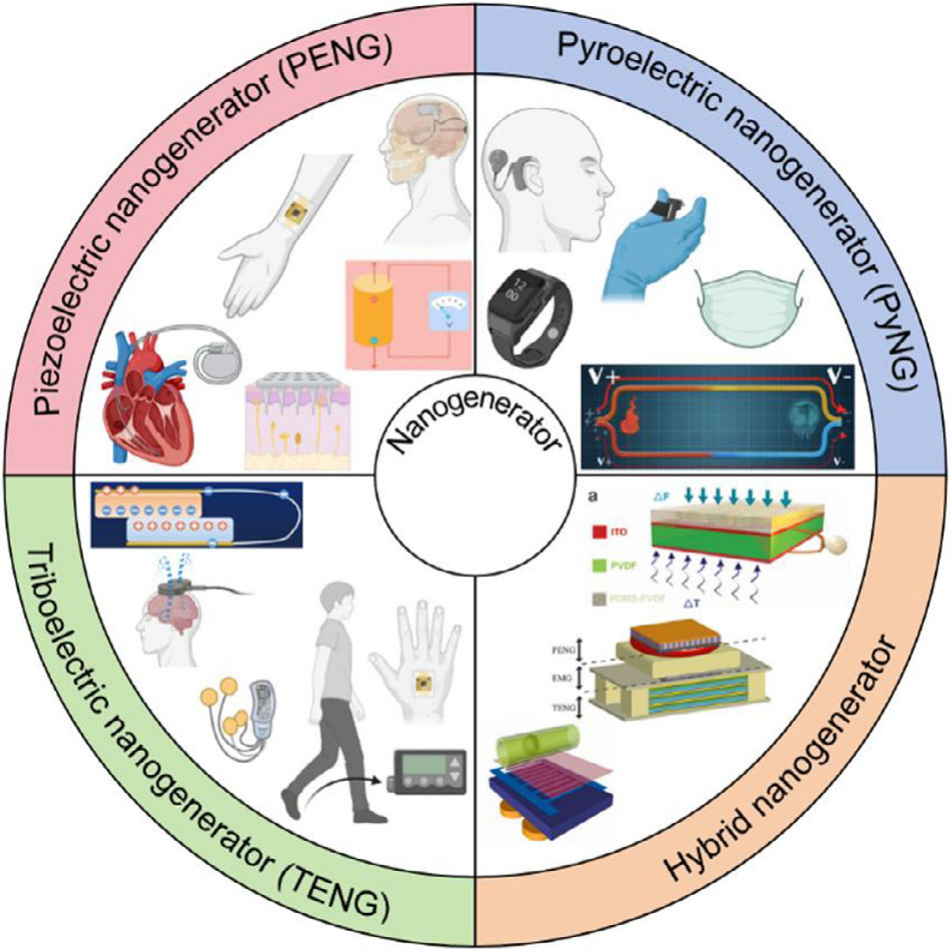
Systematic Classification of Nanogenerators. Created with BioRender 2026. License link: https://BioRender.com/k7tvtey.

Nanogenerators are classified into piezoelectric nanogenerators (PENGs), pyroelectric nanogenerators (PyNGs), triboelectric nanogenerators (TENGs), and hybrid nanogenerators (see Figure 13). Nanogenerators have gradually penetrated into multiple key scenarios such as medical diagnosis, disease treatment, and tissue engineering repair as an emerging class of functional devices featuring miniaturization, self‐powering capability, and biocompatibility. Their core characteristic lies in their ability to convert energy sources in the human body (e.g., biomechanical energy and thermal gradients) into electrical signals with specific parameters. The unique properties of these output signals, including amplitude, frequency, and waveform, can form specific coupling with the physiological electrical microenvironment in organisms, as illustrated in Table 1, thereby inducing a series of controllable and targeted biological effects—such as activating voltage‐gated calcium/potassium channels, promoting axonal growth, regulating neurotransmitter release, and enhancing synaptic plasticity.

**TABLE 1:** Output characteristics and biological effects of different nanogenerators.

	Output characteristics	Biological effects	Refs.
Piezoelectric nanogenerators (PENGs)	PENG generates electrical signals through mechanical deformation, typically producing an output voltage of 0.1–10 V, an output current at the nanoampere to microampere level, and a frequency range of 0.1–100 Hz, characteristics that closely resemble those of bioelectrical signals.	When the output voltage of PENG reaches 0.5–1.5 V, it can activate voltage‐gated sodium channels and induce neuronal action potentials; a voltage of 2–5 V effectively promotes neurite outgrowth and axonal regeneration. Low‐frequency stimulation (1–10 Hz) enhances neurite extension and synaptogenesis, while high‐frequency stimulation (50–100 Hz) suppresses neuronal hyperexcitability and is applicable in epilepsy treatment. A current density of 10–100 µA/cm^2^ effectively modulates neuronal membrane potential and promotes neural regeneration.	[138]
Pyroelectric nanogenerators (PyNGs)	Utilizing temperature gradients to generate direct current (DC) signals, with output voltages at the millivolt level and currents at the microampere range.	A temperature gradient of 0.5–2°C produces 10–50 mV, promoting neural cell migration and axonal guidance. Sustained DC stimulation guides the direction of neural regeneration and facilitates the recovery of damaged nerve function.	[139]
Triboelectric nanogenerators (TENGs)	Generate high‐voltage (10–1000 V), low‐current (nA‐level) pulsed signals via contact‐separation, with adjustable pulse width.	Short‐duration high‐voltage pulses (<1 ms) safely penetrate tissues to activate deep neurons without causing tissue damage. A pulse frequency of 10–50 Hz effectively modulates neurotransmitter release and improves synaptic plasticity. An energy density of 0.1–1 mJ/cm^2^ induces neural stem cell differentiation and promotes neural repair.	[140]
Hybrid nanogenerators	By integrating multiple energy harvesting mechanisms, output characteristics become more stable, enabling simultaneous multimodal stimulation.	The combined delivery of electrical and mechanical stimuli synergistically enhances neural regeneration, outperforming single‐mode approaches. Adaptive adjustment of output parameters based on physiological signals (e.g., ECG, EEG) enables closed‐loop neuromodulation.	[141]

#### 3.2.1 Piezoelectric Nanogenerators (PENGs)

Piezoelectric nanogenerators (PENGs) are typically composed of external driving electrodes, piezoelectric materials responsible for generating piezoelectric effects, and flexible stationary substrates. They are designed to convert tiny, irregular ambient mechanical energies—such as physical bending, ultrasonic cavitation, and vortex‐induced shear—into electrical energy through the action of piezoelectric nanomaterials [142]. The underlying mechanism relies on the behavior of electric dipoles within the piezoelectric material [143]. When subjected to an external mechanical force, the originally disordered electric dipoles realign into a regular and ordered arrangement [144, 145]. This reorientation causes the centers of positive and negative charges to shift towards opposite surfaces of the material, resulting in the generation of a potential difference across the material.

To address the challenge of mechanical flexibility, flexible PENGs based on inorganic/organic composites have been developed [146]. These composites synergistically combine the superior piezoelectric response of inorganic ceramic fillers with the high flexibility and mechanical robustness of organic polymer matrices. As a result, these hybrid energy harvesters are capable of maintaining stable power output under a wide range of mechanical deformations and efficiently capturing green energy from sources such as airflow, raindrops, and biomechanical motions [147]. This versatility makes them promising candidates as sustainable alternative energy sources, particularly for enhancing the applicability of smart Internet of Things (IoT) systems [148].

**FIGURE 14:**
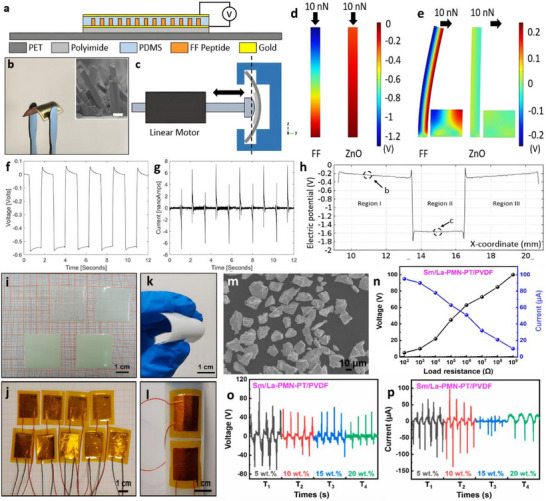
(a) Schematic of the FF peptide nanogenerator attached to a PET beam. (b) Image of FF peptide nanogenerator highlighting the flexible nature of the polyimide substrate. Inset shows SEM image of peptide microrods. Scale bar is 10 µm. (c) Test setup schematic showing nanogenerator and PET beam with periodic displacement applied by a linear motor. (d) Piezoelectric potential in a y‐z plane slice for FF peptide (left) and ZnO (right) nanowires under 10 nN compressive force. (e) Piezoelectric potential in a y‐z plane slice for FF peptide (left) and ZnO (right) nanowires under 10 nN transverse force. Insets show regions of potential reversal at the nanowire roots. (f) Open‐circuit voltage (*V*_oc_) and (g) short‐circuit current (*I*_sc_) for the peptide nanogenerator under periodic 3.5 mm displacement. (h) Electric potential at the top of the FF microrods as a function of position on the deformed nanogenerator. Reproduced with permission [149]. Copyright 2018, Elsevier. (i) The photo of Sm/La‐PMN‐PT ceramic disks. (j) The photo of Sm/La‐PMN‐PT/PVDF and Sm/La‐PMN‐PT/PDMS PENG devices. (k) The bending status of the composite film. (l) The devices can be wrapped on the surface of the cylinder. (m) Morphologies from SEM images of the Sm/La‐PMN‐PT powders. (n) The output voltage/current, (o) voltage, and (p) current with the external load resistance ranging from 102 to 109 Ω of 10wt.% Sm/La‐PMN‐PT/PVDF PENG device. Reproduced with permission [150]. Copyright 2024, MDPI.

Recent research has introduced innovative strategies to further enhance the performance of piezoelectric nanogenerators (PENGs). For example, a straightforward epitaxial growth method was utilized to fabricate double‐layered phenylalanine–phenylalanine (FF) peptide microrods, which were then aligned to construct an integrated double‐layer structured PENG (see Figures 14a–c). By precisely controlling the polarization orientations of the two FF microrod layers, the device exhibited significantly improved output voltage and current, providing new insights into molecular‐level engineering of piezoelectric systems [149].

Figures 14c–d illustrate the piezoelectric potentials in the y–z plane slices of FF peptide (left) and ZnO (right) nanowires subjected to compressive and lateral forces of 10 nanonewtons, respectively. Subsequently, the open‐circuit voltage, short‐circuit current, and position‐dependent responses of the peptide nanogenerator were evaluated under periodic displacements of 3.5 mm (see Figure 14f–h). These results underscore the promising application potential of FF peptides as piezoelectric materials and highlight the crucial role of finite element modeling in mechanism analysis and structural optimization. This work lays both theoretical and experimental foundations for the design of high‐performance, biocompatible peptide‐based piezoelectric devices.

In another study, a flexible PENG was developed by embedding co‐doped rare‐earth element ceramics (RE‐PMN‐PT) into a composite film of PVDF and PDMS (see Figure 14i‐l). Figure 14m presents a scanning electron microscopy (SEM) image of Sm/La‐PMN‐PT powder, indicating the material's favorable morphology. The open‐circuit voltage and short‐circuit current of Sm/La‐PMN‐PT/PVDF reach 98 V and 100 µA (see Figure 14n), respectively. Figure 14o reveals that the composite with 10 wt.% Sm/La‐PMN‐PT/PVDF exhibits the highest output voltage, while exceeding this proportion leads to a significant decline in short‐circuit current (see Figure 14p), suggesting the device's potential for energy harvesting in biomedical applications [150]. This design not only achieved significant output performance but also eliminated the need for a high‐voltage electrical poling process, simplifying device fabrication and improving operational safety.

The piezoelectric nanogenerator (PENG) based on barium titanate (BTO) exhibits output characteristics of high voltage (84 V), low current (1.32 µA), and short pulses (pulse width: 0.1–1 ms), which are significantly lower than the milliampere‐level current of typical vagus nerve stimulation (VNS) devices. This output profile meets the requirements of low charge injection, thereby mitigating the risk of tissue damage. Notably, PENG maintains stable output under low‐frequency mechanical stimulation (1–3 Hz), which precisely matches the physiological rhythm of carotid artery pulsation (1–2 Hz). Mechanistically, PENG delivers electrical stimulation generated by capacitor charging and discharge to activate the cholinergic anti‐inflammatory pathway (CAP) via vagus nerve stimulation. Acetylcholine (ACh) released upon stimulation inhibits macrophage inflammasome activation while promoting regulatory T cell (Treg) differentiation, thereby enhancing the anti‐inflammatory effect. Furthermore, α7 nicotinic acetylcholine receptor (α7nAChR) activation induces phosphorylation of the JAK2‐STAT3 signaling pathway, which suppresses the nuclear translocation of NF‐κB and subsequently blocks the transcription of tumor necrosis factor‐α (TNF‐α) [151].

Today, PENGs have broadened their applications beyond traditional energy harvesting, becoming a cornerstone in the development of self‐powered biomedical devices. They have been successfully explored in powering self‐sustained sensors, cardiac pacemakers, deep brain stimulators, drug delivery systems, electronic skin (e‐skin), and tissue regeneration platforms. These advancements position PENGs at the forefront of personalized medicine, offering wireless, efficient, and biocompatible solutions for future healthcare technologies [152].

#### 3.2.2 Pyroelectric Nanogenerators (PyNGs)

In daily life, a substantial amount of heat is wasted through various channels, such as the temperature difference between the human body and the environment during winter, temperature fluctuations around the nasal cavity during breathing, the discharge of hot water during industrial cooling processes, and the dissipation of heat generated by air conditioning systems into the environment [153]. Converting this abundant waste heat into usable electrical energy offers a promising strategy to address global energy shortages.

Compared to piezoelectric nanogenerators (PENGs) and triboelectric nanogenerators (TENGs), research on pyroelectric nanogenerators (PyNGs) has been relatively limited [154]. PyNGs operate based on the Seebeck effect and the pyroelectric effect, enabling the direct conversion of thermal gradients into electrical energy [155]. Notably, PyNGs can produce substantial output power under relatively small temperature differences. Unlike PENGs and TENGs, PyNGs do not rely on mechanical deformation for energy harvesting, thereby circumventing challenges related to mechanical durability and structural fatigue [156]. Structurally, a typical PyNG consists of three essential components: an upper metal layer (serving as the upper electrode) designed for efficient heat collection; a middle pyroelectric layer responsible for converting thermal energy into electrical energy through changes in internal polarization; and a lower metal layer that acts as the bottom electrode. Through this layered architecture, PyNGs can effectively generate electrical output in response to temperature variations [157].

**FIGURE 15:**
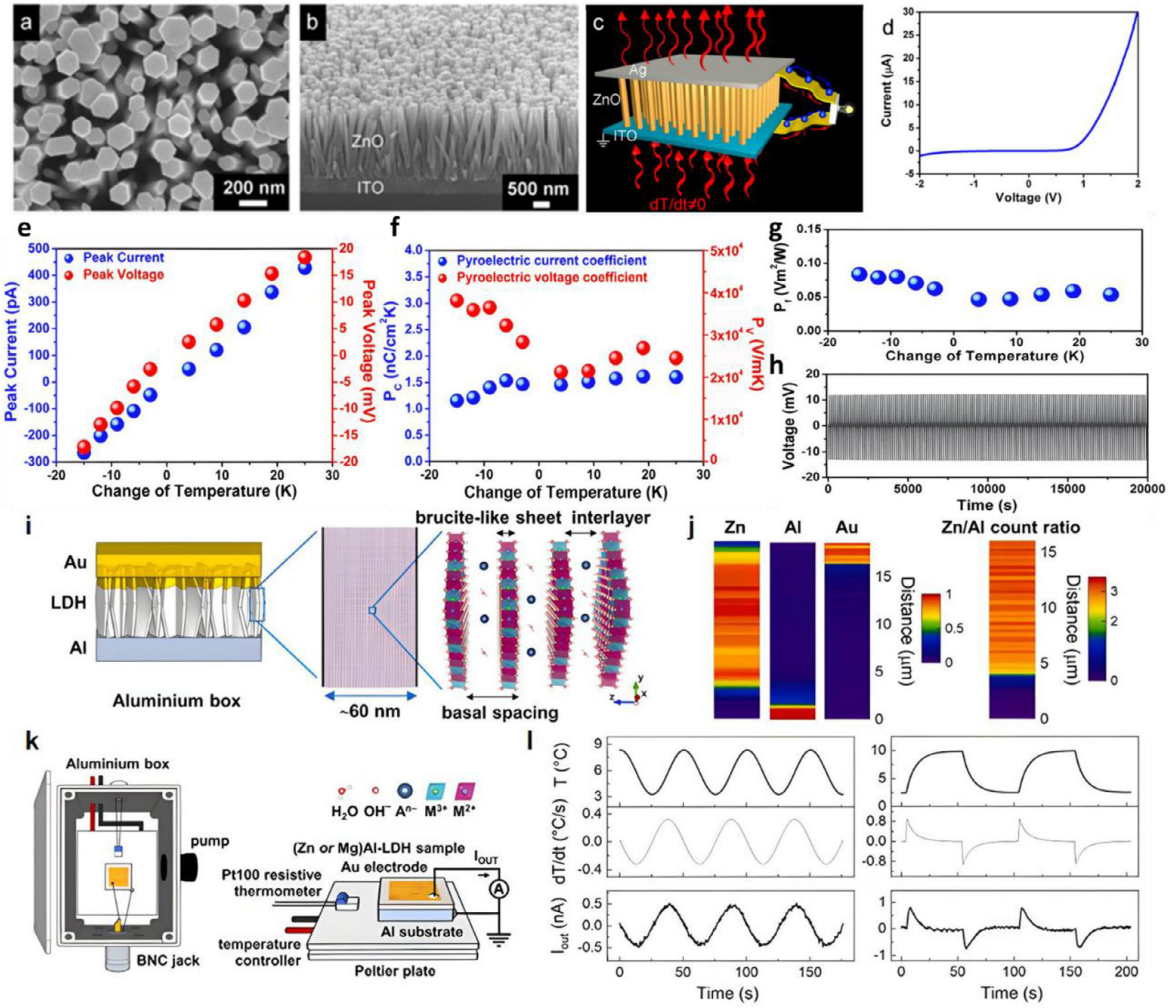
(a) SEM image of the as‐grown ZnO nanowire array. (b) Tilted cross‐sectional SEM image of the ZnO nanowire array. (c) Schematic diagram showing the structure of the pyroelectric nanogenerator. (d) *I−V* characteristics of the nanogenerator measured at room temperature, showing the presence of a Schottky contact between Ag and ZnO nanowires. The output peak voltage/current (e), pyroelectric voltage/current coefficients (f), and energy conversion characteristic value (g), as a function of change in temperature. (h) Output stability test of the nanogenerator through cyclic temperature change for over 200 cycles. Reproduced with permission [158]. Copyright 2012, American Chemical Society. (i) Schematics showing the multilayered Au/LDH/Al device structure (left panel), magnified view of the two‐dimensional lamellar structure of a ∼60 nm thick LDH nanoplatelet (central panel), and hydrotalcite‐type crystal structure (3 R polytype) of an LDH (right panel). (j) Contours of normalized depth profiles of Zn, Al, and Au (left panel) and of Zn/Al count ratio (right panel) from the EDS line scan data. (k) Schematic illustration of the experimental setup for characterizing the pyroelectric nanogenerators. (l) Measured temperature, calculated derivative of temperature with respect to time, and short circuit output current Iout of the ZnAl LDH pyroelectric nanogenerator under sinusoidal (left panels) and square‐wave (right panel) cyclic changes in the Peltier heating current. Reproduced with permission [159]. Copyright 2023, Elsevier.

Pyroelectric nanogenerators have shown significant potential for applications in infrared sensing, body temperature monitoring, thermal imaging, electronic skin, and wearable health monitoring devices. The nanogenerator based on pyroelectric ZnO nanowire arrays, which converts thermal energy into electrical energy, provides a foundation for self‐powered nanotechnology to harvest thermal energy from ambient temperature fluctuations [158]. Figure 15a–c shows the SEM images and structural schematics of the ZnO nanowire array. Then, the *I*–*V* characteristics of the nanogenerator at room temperature were measured, along with the changes in output peak voltage/current, thermoelectric voltage/current coefficients, and energy conversion characteristic values with temperature (see Figure 15d–g). Comparative experiments were conducted using the Ag‐ITO film and the Ag‐TiO_2_ nanowire array‐ITO structure. Under temperature fluctuations, no obvious voltage/current pulses were observed, and the linear superposition test showed that the output of the nanogenerator conforms to the characteristics of the pyroelectric effect and maintains high output stability (see Figure 15h). Figure 15i,k shows the schematic diagram of the multi‐layer Au/LDH/Al device structure and the experimental setup for characterizing the pyroelectric nanogenerator, respectively. All curves in Figure 15l indicate that the output short‐circuit current of the ZnAl LDH pyroelectric nanogenerator is nearly perfectly proportional to the rate of temperature change, consistent with the expectations for thermoelectric devices [159]. Some studies have introduced novel nano‐engineering methods to enhance PyNG performance. For instance, an Ag_2_Se/polyvinylpyrrolidone (PVP) composite film was fabricated on a nylon membrane, resulting in a flexible material with a unique microstructure. A six‐leg flexible thermoelectric generator (F‐TEG) assembled with this optimized film achieved a maximum power output of 4.58 µW, corresponding to a high power density of approximately 31.2 W m^−^^2^ under a temperature gradient of 38.7 K [160].

Furthermore, pyroelectric nanogenerators based on layered double hydroxides (LDHs) have demonstrated remarkable properties, including facile fabrication of high‐density aligned nanostructures over large and flexible substrates, high surface‐to‐volume ratios, excellent biocompatibility, inherent flame retardancy, and multiple degrees of compositional tunability (such as adjusting the ratio of divalent to trivalent cations, intercalated anions, and nanostructure geometries). These tunable features allow for the precise engineering of LDH‐based PyNGs to optimize their band structures and energy harvesting capabilities [159].

In patients with asthma, PyNG can generate pulsed electrical signals whose output pattern is highly compatible with the physiological electrical activity of the nervous system. This device enables real‐time monitoring of respiratory pattern changes; upon detecting abnormal breathing, it can stimulate vagal nerve terminals and activate the vagus‐adrenal axis. Following vagal nerve excitation, neurotransmitters (e.g., acetylcholine) act on the adrenal medulla to promote the release of catecholamines (norepinephrine and epinephrine). These neurotransmitters can inhibit the excessive activation of immune cells such as macrophages and neutrophils, thereby alleviating airway inflammatory responses. Additionally, electrical stimulation by PyNG induces neurons to secrete neuropeptides (e.g., substance P). Specifically, substance P enhances the phagocytic function of macrophages, while calcitonin gene‐related peptide (CGRP) suppresses T‐cell activation, achieving precise immunomodulation [139]. Collectively, PyNG exhibits considerable therapeutic potential for respiratory diseases such as asthma and chronic obstructive pulmonary disease (COPD).

Integrating PyNGs into respirators or joint regions enables the creation of wearable energy harvesting devices capable of converting waste heat from respiration or joint motion into electrical signals [161]. Such wearable sensors are critically important for real‐time human health monitoring, especially for elderly individuals, asthma patients, and people with mobility impairments [162, 163]. Overall, the integration of PyNGs into wearable systems not only enhances energy sustainability by utilizing otherwise wasted heat but also opens new avenues for non‐invasive, continuous health monitoring technologies.

#### 3.2.3 Triboelectric Nanogenerators (TENGs)

Triboelectric nanogenerators (TENGs) represent a novel class of sustainable energy harvesting devices that do not require external power sources. Based on Maxwell's theory of displacement current, TENGs utilize the electrostatic induction effect generated by the contact and separation of two different materials [164, 165]. When these materials are separated due to external forces, the frictional charges induce the movement of electrons and holes to compensate for the surface potential difference, generating a reverse current. As a result, TENGs can effectively generate alternating current (AC) from the mechanical processes of contact and separation, efficiently converting amorphous mechanical energy into electrical energy [17].

**FIGURE 16:**
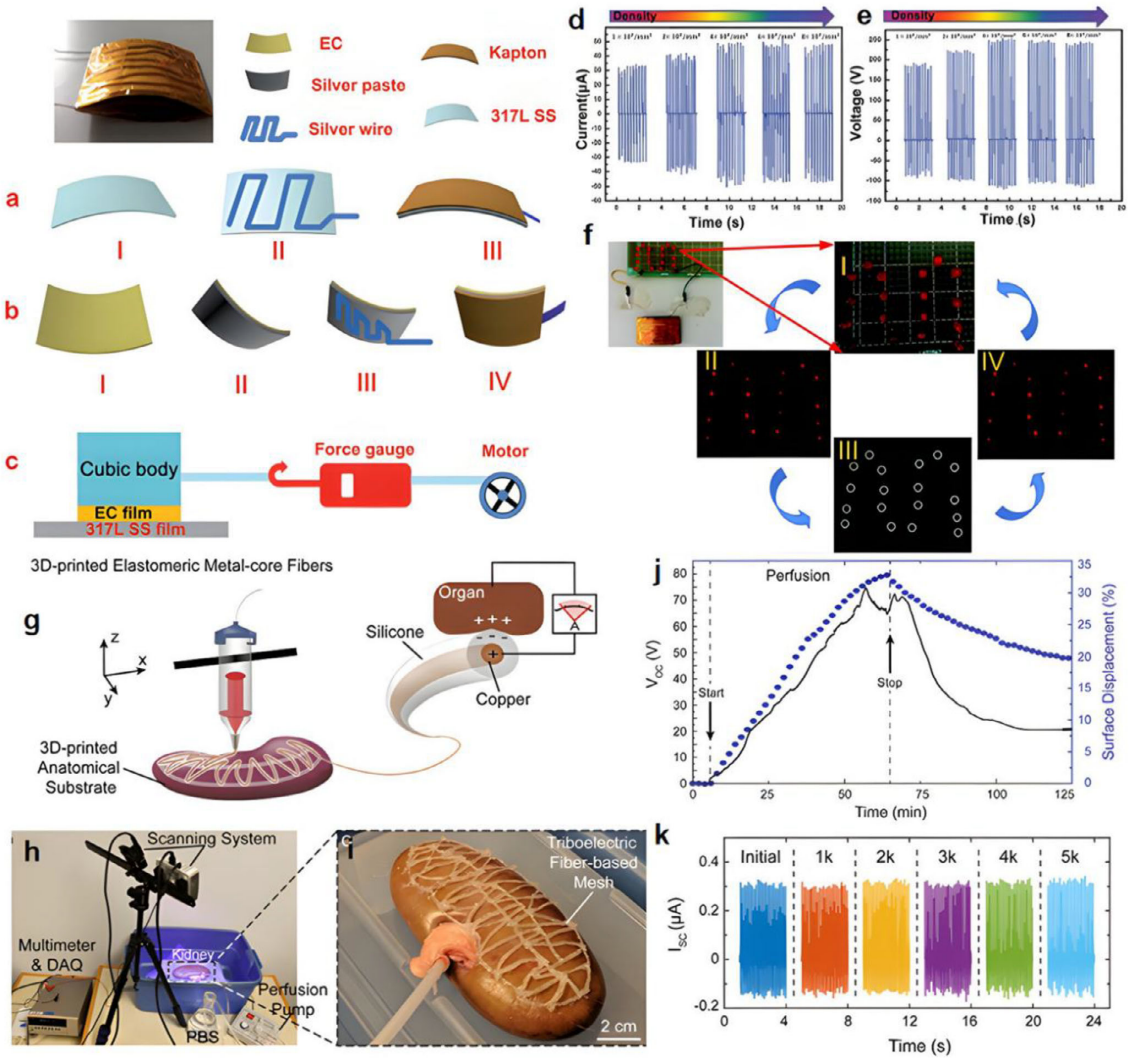
(a) Fabrication process flowchart for (a) top plate and (b) bottom plate of the TENG. (c) The testing device for the friction force. (d‐e) Their corresponding short‐circuit current and open‐circuit voltage. (f) Snapshots of eighteen TENG‐driven flashing paralleled commercial LEDs: (II), (IV), and (I), (III) show two processes: before applying the external force and continuing to exert force for a few seconds, respectively. Reproduced with permission [167]. Copyright 2017, Royal Society of Chemistry. (g) Schematic illustrating conformal 3D printing of elastomeric metal‐core TENG fibers on objects with organic shape, specifically, a 3D‐printed porcine kidney model, for fabrication of form‐fitting wearable triboelectric devices. (h) Photograph of the custom machine perfusion apparatus. (i) Photograph of the 3D‐printed kidney‐conforming TENG fiber‐based mesh sensor. (j) Real‐time responses of organ displacement associated with perfusion‐induced edema acquired using 3D scanning, shown with the corresponding *V*_OC_ response of the 3D‐printed TENG fiber‐based mesh sensor. (k) Short‐circuit current generated in the durability tests. Reproduced with permission [168]. Copyright 2020, Elsevier.

The simple and rigid structure of TENGs enables stable operation even in harsh conditions [166]. Their ability to generate high voltages and low currents avoids safety issues associated with conventional energy sources, such as battery explosions or semiconductor overheating [13]. Recent research has introduced biomedical TENGs based on the triboelectric effect and electrostatic induction between biocompatible, medical‐grade 317L stainless steel (317L SS) plates and ethyl cellulose (EC) membranes [167]. Figures 16a‐c show the manufacturing flow chart of the TENG and the friction force testing device. Under optimal conditions, the open‐circuit voltage and short‐circuit current of the TENG can reach 245 V and 50 µA (see Figure 16d‐e). As shown in Figure 16f, to further clarify the working principle of the triboelectric nanogenerator, it was used as a direct power source to supply power to LEDs without any rectification or energy storage devices [167]. The surfaces of these materials were engineered using photolithography and inductively coupled plasma (ICP) etching techniques to optimize their triboelectric performance.

A novel fabrication strategy has emerged. As shown in Figure 16i,j, this strategy utilizes elastomeric metal‐cored triboelectric nanogenerator (TENG) fibers to enable the preparation of stretchable films, meshes, and hollow three‐dimensional structures via 3D printing on planar, rotating, and non‐planar anatomical substrates [168]. The triboelectric properties of these 3D‐printed elastomeric metal‐cored silicone‐copper (Cu, cladding‐core) fibers were quantitatively analyzed under cyclic loading tests. The data in Figure 16j demonstrates that 3D‐printed conformable structures composed of silicone‐copper triboelectric generator fibers provide attractive self‐powered, wearable force sensors for organ preservation and biomanufacturing applications, capable of real‐time monitoring of perfusion‐induced edema. Additionally, no obvious decay in short‐circuit current (ISC) was observed in silicone‐copper triboelectric nanogenerator (TENG) fibers and 3D‐printed structures after 5000 loading cycles, indicating their reliability as sensing and energy harvesting devices (see Figure 16k).

A triboelectric nanogenerator (TENG) with a multi‐gap structure can generate more output current pulses per driving cycle. The high‐voltage, low‐current output profile effectively stimulates neural tissues while avoiding the risk of tissue damage. TENG exhibits high sensitivity to minute mechanical changes. For instance, a single‐electrode mode TENG based on conductive sponge demonstrates a pressure sensitivity of 3.5 V/N, an ultralow force detection limit of 14 mg, and a response time of only 72 ms. Electrical stimulation generated by TENG effectively inhibits the polarization of microglia toward the pro‐inflammatory M1 phenotype, thereby alleviating neuroinflammation and brain injury. Through the NF‐κB pathway, TENG‐mediated stimulation reduces astrocyte activation and suppresses the expression of pro‐inflammatory cytokines such as IL‐1β and TNF‐α. Furthermore, it promotes the release of neuropeptides, including substance P and calcitonin gene‐related peptide (CGRP), from neurons, which in turn modulates microglial function [169].

TENGs are increasingly utilized in healthcare applications, including gait phase detection, wearable assistance, touch sensors, smart monitoring systems, extracorporeal pacemakers, and electronic skins (e‐skins) [170]. They offer a promising solution for self‐powered electricity generation due to their advantages: they are self‐sustaining, safe, high‐output, low‐cost, flexible, lightweight, and made from widely sourced materials.

#### 3.2.4 Hybrid Nanogenerators

Hybrid nanogenerators are advanced energy harvesting devices that can simultaneously or alternately harness various ambient energy sources. By integrating multiple energy conversion mechanisms—such as triboelectric, piezoelectric, thermoelectric, photovoltaic, and electromagnetic—the performance and efficiency of these devices are significantly enhanced. This combination of mechanisms improves energy harvesting efficiency and adaptability to different environmental conditions, making hybrid nanogenerators a prominent research topic in the energy field in recent years.

One of the most promising types of hybrid nanogenerators is the triboelectric‐piezoelectric hybrid nanogenerator. This device integrates both the triboelectric and piezoelectric effects, enabling it to harness mechanical energy from the environment, such as vibrations and human body movements, either simultaneously or alternately. By combining these two energy conversion mechanisms, the hybrid generator significantly improves energy‐harvesting efficiency and expands the range of potential applications [171]. The design typically incorporates both a triboelectric layer and a piezoelectric layer within the same device, allowing mechanical energy to stimulate both effects concurrently. This results in a combined energy output. The triboelectric effect is particularly effective for harvesting low‐frequency mechanical energy, while the piezoelectric effect is more suited for high‐frequency mechanical energy. Together, they complement each other, enhancing overall energy efficiency.

Recent studies have demonstrated that more efficient triboelectric nanogenerators (TENGs) can be fabricated using inversely polarized ferroelectric films, which are made of the same material as the contacting layers. A notable correlation was found between the piezoelectric response of counter‐polarized ferroelectric PVDF/BaTiO_3_ films and the amplified electrostatic induction driven by both the piezoelectric charges and the ferroelectric properties of these materials [172]. This hybrid tribo‐piezoelectric nanogenerator effectively captures the interaction between the ferroelectric layers during the contact‐separation process and the subsequent charge redistribution in the external circuit.

In another advancement, researchers have integrated flexible thermoelectric and piezoelectric materials into a single device structure. In this configuration, carbon nanotube/polymer films serve as flexible thermoelectric generators while also functioning as electrodes for a piezoelectric generator made from polyvinylidene fluoride (PVDF) [173]. This innovative design addresses several challenges faced by traditional thermoelectric and piezoelectric generator combinations, optimizing the overall power output.

**FIGURE 17:**
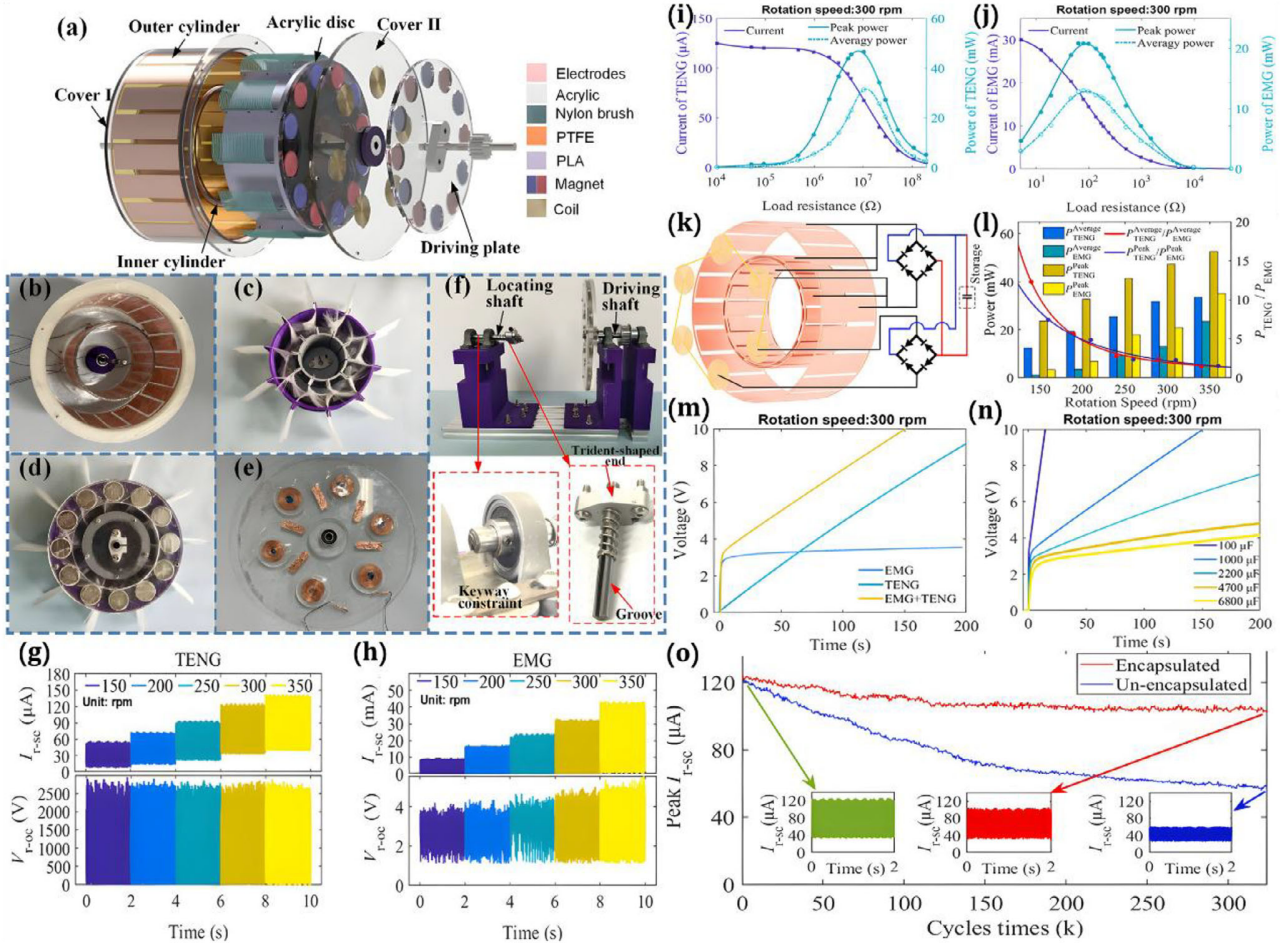
Architecture of the hybrid generator: (a) components of the hybrid generator. Photographs of (b) the internal of the stator, (c) the rotator with multilayered brushes, (d) part of the magnetic coupling on the rotator, (e) cover II attached to home‐made Cu coils, and (f) the 3D printed support with driving shaft, locating shaft, and driving plate. The insets are the enlarged views of the keyway constraint and the locating shaft with the trident‐shaped end and the groove, respectively. The electricity output of (g) MBC‐TENG and (h) RD‐EMG at different rotation speeds. Dependences of output power and generated current on the load resistance for (i) MBC‐TENG and (j) RD‐EMG. (k) the equivalent circuit for the hybrid generator. (l)The ratios of the output power of MBC‐TENG to RD‐EMG at different rotation speeds. (m) The charging performances of the MBC‐TENG, EMG and the hybrid generator for a 1000 µF capacitor at the rotation speed of 300 rpm. (n) The charging performances of the hybrid generator for different capacitors at 300 rpm. (o) Variation of the peak rectified short‐circuit current for the MBC‐TENG in the encapsulated and the un‐encapsulated cases under continuous measurement for 324 000 cycles. Reproduced with permission [174]. Copyright 2021, Elsevier.

Additionally, a wave energy conversion device based on a heaving point absorber has been developed, integrating a multi‐layer soft brush cylindrical triboelectric nanogenerator (MBC‐TENG) with a rotary disc electromagnetic generator (RD‐EMG) (see Figure 17a–f). The MBC‐TENG comprises a stator—consisting of an acrylic cylinder, cover, and copper electrode coated with PTFE film—and a rotor made of an acrylic disc, PLA cylinder, nylon soft brush, and magnet. The RD‐EMG features six copper synchronous wound coils arranged on the inner surface of Cover II [173].

Figure 17g–l systematically illustrates the power performance of the optimized MBC‐TENG and RD‐EMG hybrid system. For the RD‐EMG, as the rotational speed increases from 150 to 350 rpm, the open‐circuit voltage rises linearly from 4 V to 5.5 V, while the current increases from 10 mA to 45 mA. The charging performance and stability of the hybrid nanogenerator were further evaluated (see Figure 17m–o), demonstrating stable and tunable power output over a wide range of rotational speeds.

This combined system offers an effective solution for converting dynamic mechanical energy into electrical energy, with promising applications in self‐powered sensing, wearable devices, and other fields. Importantly, the device provides a reliable power source for marine environments, capable of powering commercial Bluetooth temperature and humidity sensing systems (BTHSS) and illuminating numerous LED lights.

#### 3.2.5 Emerging Types of Nanogenerators

Apart from the aforementioned nanogenerators, several emerging types of nanogenerators have emerged, such as bio‐nanogenerators. These generate electrical energy by leveraging biological materials or biological processes (such as cell metabolism). These bio‐nanogenerators demonstrate extensive application potential in fields including biomedicine, environmental monitoring, and wearable devices (Table 2).

**TABLE 2:** Classification of bio‐nanogenerators.

Types of nanogenerators	Operation mechanism	Advantages	Disadvantages	Application	Refs.
Cell‐driven nanogenerator	Electrical energy is generated by harnessing the metabolic processes or mechanical motion (e.g., contraction of muscle cells) of living cells. Such generators enable direct energy extraction from biological organisms.	Good biocompatibility, sustainable energy supply, and strong adaptability.	Low output power, complex synthesis technology, and the stability need to be improved.	Drug delivery system, water quality and soil monitoring, bioenergy production, self‐powered sensor network, bio‐robot	[175]
Microbial fuel cells (MFCs)	Electricity is generated by microbes through their metabolic processes, converting organic matter into electrical energy. These devices utilize the microbial breakdown of organic matter to produce electrical current.	Mild reaction conditions, and capable of in‐situ power generation	Low power density and poor microbial adaptability.	Energy recovery, water quality purification, soil remediation, environmental monitoring, bioenergy production	[176]
Enzyme‐catalyzed nanogenerator	Electricity generation through enzyme‐catalyzed reactions is typically achieved via electrochemical processes. Such enzymatic biofuel cells can be applied both in vivo and in vitro.	High catalytic efficiency and strong specificity.	Poor enzyme stability and high substrate dependence.	Power supply for implantable medical devices, drug release control, environmental monitoring field, and self‐powered system	[177]

Photoelectric nanogenerators are based on the photoelectric effect, generating electrical energy by utilizing light illumination. They exhibit broad application potential in fields such as solar energy utilization, environmental monitoring, and smart devices (Table 3).

**TABLE 3:** Classification of photoelectric nanogenerators.

Types of nanogenerators	Operation mechanism	Advantages	Disadvantages	Application	Refs.
Photovoltaic nanogenerator	The photovoltaic effect enables the direct conversion of light energy into electricity through semiconductor materials such as silicon and perovskites, making it a cornerstone of solar power generation.	High‐efficiency photoelectric conversion, good stability, and easy integration.	Limited by quantum efficiency and restricted in material selection.	Distributed generation, biomedical monitoring, environmental monitoring, self‐powered display screen, power supply for transportation facilities	[178]
Photosensitive material nanogenerator	Photosensitive materials, such as organic photosensitizers, generate electrical current under illumination, enabling energy harvesting in low‐light environments.	Wide spectral response range, high sensitivity, and flexibility.	Problems with photostability, complex preparation process, and limited working environment.	Aerospace, Microelectronic Devices, Self‐powered Photoelectric Detector, Biomedical Sensor, Environmental Monitoring Sensor	[179]
Photoelectrochemical nanogenerator	Photocatalytic systems utilize light‐driven excitation of catalysts to enable electricity generation through enhanced chemical reactions, with applications in water splitting and organic compound conversion.	High‐efficiency photoelectric conversion, strong customizability, and wide potential applications.	Problems of photocorrosion, serious charge recombination, and poor electrolyte compatibility.	Portable power supply, Water quality monitoring, Air pollutant detection, Implantable medical devices, Smart display	[180]

Chemical nanogenerators produce electrical energy through chemical reactions such as fuel cell reactions or electrochemical reactions. They exhibit extensive application potential in fields such as energy conversion, environmental monitoring, and biomedicine (Table 4).

**TABLE 4:** Classification of chemical nanogenerators.

Types of nanogenerators	Operation mechanism	Advantages	Disadvantages	Application	Refs.
Lithium‐ion battery	Although primarily employed for energy storage, certain systems can be redefined as chemical nanogenerators, where electrical current is generated through lithium‐ion migration.	High energy density, low self‐discharge rate, high output voltage, and long cycle life.	Safety issues, relatively high cost, and poor performance at low temperatures.	Consumer electronics products, Electric vehicles, Energy storage field, Pacemaker, Insulin pump, Portable ventilator	[181]
Fuel battery	Electrical energy is generated through the electrochemical reaction of hydrogen or other fuels with oxygen, typically employing proton exchange membranes (PEMs) or solid oxide fuel cells (SOFCs).	High energy conversion efficiency, strong flexibility, and low pollution.	Limited fuel supply, high cost, and limited lifespan.	Transportation field, Distributed generation field, Portable power supply field, Aerospace field	[182]

Acoustic nanogenerators generate electrical energy by harnessing the vibrations of sound waves. They demonstrate broad application potential in fields such as environmental monitoring, wearable devices, and intelligent sensors (Table 5).

**TABLE 5:** Classification of acoustic nanogenerators.

Types of nanogenerators	Operation mechanism	Advantages	Disadvantages	Application	Refs.
Ultrasonic generator	Ultrasound waves (with a frequency greater than 20 kHz) induce the resonance of nanosheets, and the piezoelectric effect outputs a voltage of approximately 10 volts.	High energy conversion efficiency, advantages of nanoscale properties, and controllability.	Challenges in material preparation, difficulties in performance optimization, and limited application scope.	Self‐powered sensors for medical ultrasonic imaging devices	[183]
Bionic resonant cavity nanogenerator	Mimicking the auditory organs of insects (such as the antennae of crickets), a microcavity structure is adopted to focus sound waves of specific frequencies, achieving a selective enhancement of sound waves in the 2–5 kHz range and improving the efficiency by 40%.	Efficient energy capture, good biocompatibility, and high sensitivity.	Complex preparation process, strong frequency selectivity, and limited energy output.	Energy relay node of a directional acoustic communication system	[184]
Biohybrid nanogenerators	Leverage the unique mechanical or electrical properties of biological materials (such as proteins, DNA, or bacterial cellulose) and combine them with acoustic wave vibrations to generate electrical energy.	Good biocompatibility, diverse energy sources, and self‐powered characteristics.	Relatively low energy output, performance is easily affected by interference, and has a limited lifespan.	Acoustic‐driven energy supply for implantable medical devices (e.g., cardiac pacemakers).	[185]

Electromagnetic nanogenerators utilize the principle of electromagnetic induction to convert mechanical motion into electrical energy. These electromagnetic nanogenerators exhibit extensive application potential in fields such as energy harvesting, environmental monitoring, and power supply for miniature devices (Table 6).

**TABLE 6:** Classification of electromagnetic nanogenerators.

Types of nanogenerators	Operation mechanism	Advantages	Disadvantages	Application	Refs.
Electromagnetic induction generator	Leveraging Faraday's law of electromagnetic induction, electrical current is generated through magnetic field variations or conductor motion, enabling efficient energy conversion from vibrational or kinetic sources.	Mature technological development, strong adaptability, efficient and stable output.	Large volume and weight, low energy density, existence of electromagnetic interference.	Power production, Transportation, Industrial automation equipment, Household wind power generation equipment, Hand‐cranked power generation equipment	[186]
Electromagnetic vibration generator	Harnessing mechanical vibrations and electromagnetic induction, electrical energy is generated through vibration‐induced relative motion, enabling the harvesting of ambient vibrational energy.	Fast response speed, wide range of applicable scenarios, and efficient energy conversion.	Energy harvesting depends on vibration conditions, limited output power, and relatively large size.	Medical devices, Biomedical detection, Vibration monitoring and measurement field, Distributed energy system	[187]
Magnetohydrodynamic generator	Exploiting the motion of ferrofluids under magnetic fields enables electrical current generation, suitable for energy conversion in dynamic environments.	Rapid response, no rotating parts, can use a variety of fuels, and has high energy conversion efficiency.	Requirements for high temperature and strong magnetic field, complex system, and limited application scope.	Nuclear energy, Solar power generation, Aircraft power system, Power supply for military equipment, Metallurgical industry, Chemical industry	[188]

As emerging representatives of micro‐nano energy harvesting technology, the five types of nanogenerators—bio‐nanogenerators, photoelectric nanogenerators, chemical nanogenerators, acoustic nanogenerators, and electromagnetic nanogenerators—break through the limitations of traditional energy supply by virtue of their innovative energy conversion mechanisms. Each targets a unique form of accessible energy: bio‐nanogenerators tap into sustainable biomass energy from biological systems, photoelectric ones harness ubiquitous light energy, chemical ones capitalize on energy stored in chemical reactions or trace substances, acoustic ones capture unused vibration energy from sound waves, and electromagnetic ones retrieve energy from magnetic field changes or electromagnetic radiation. Despite their distinct working principles and energy sources, these emerging devices share core advantages of miniaturization, low environmental impact, and high adaptability to extreme or confined scenarios, filling the gap in self‐power supply for micro‐nano electronic devices [189]. Their emergence not only enriches the technical system of distributed energy harvesting but also paves the way for the development of cable‐free, long‐endurance technologies in fields such as wearable electronics, implantable medical devices, IoT sensor networks, and environmental monitoring—heralding a new era of “energy‐on‐demand” for microscale systems.

### 3.3 Advanced Fabrication Methodologies

**FIGURE 18:**
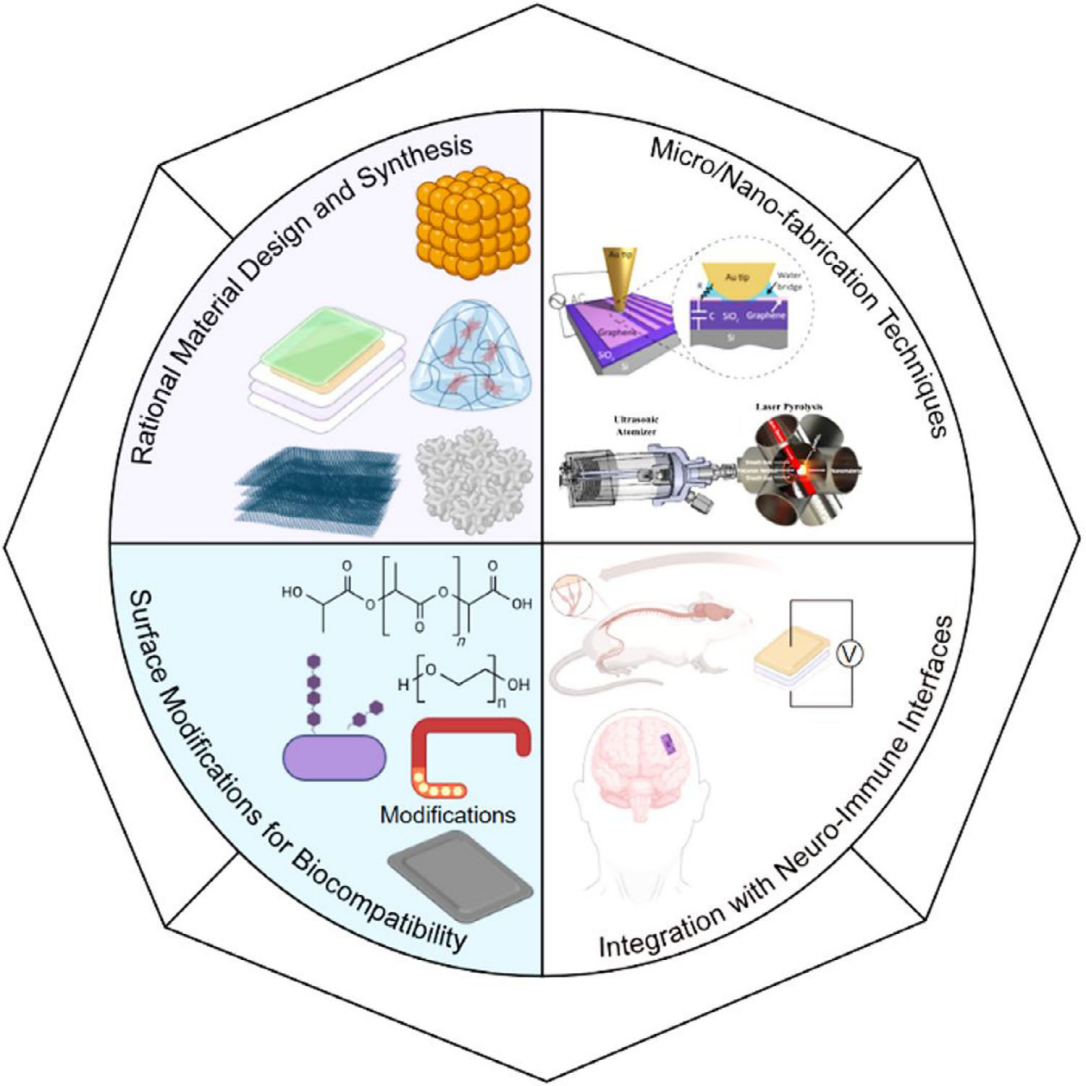
Advanced fabrication methodologies. Created with BioRender 2026. License link: https://BioRender.com/k7tvtey.

The advanced manufacturing methods for nanogenerators encompass several key aspects. First and foremost is the rational design and synthesis of materials. Materials with superior piezoelectric or pyroelectric properties are carefully selected to optimize energy conversion efficiency. Next, micro/nanofabrication techniques, such as photolithography and electron beam lithography, are employed to precisely construct nanostructures, ensuring the devices’ performance and reliability. Additionally, biocompatible surface modification plays a crucial role. By coating the nanogenerators with polymers or bioactive molecules, their stability in biological environments is enhanced, and immune responses are minimized. Finally, integrating neuroimmune interfaces enables nanogenerators to efficiently harvest energy while interacting seamlessly with biological systems, paving the way for their application in the biomedical field (see Figure 18). The synergistic effect of these methods collectively drives the development of nanogenerators, showcasing their immense potential in energy harvesting and medical monitoring.

#### 3.3.1 Rational Material Design and Synthesis

Materials for fabricating nanogenerators should exhibit a combination of excellent electrical properties, outstanding mechanical characteristics, chemical stability, favorable surface traits, environmental friendliness, ease of preparation and processing, and versatility. Enhancing these properties will facilitate the widespread application of nanogenerators across various domains and accelerate the development of sustainable energy technologies. Through material design and structural optimization, the electrical conductivity of these materials can be further improved to meet the demands of different applications.

The fundamental function of a nanogenerator is to convert mechanical energy into electrical energy, necessitating materials with superior electrical properties such as high conductivity and a high dielectric constant [190]. Common materials exhibiting high electrical conductivity include metal nanoparticles, conductive polymers (e.g., polyaniline and polypyrrole), and carbon‐based materials (e.g., graphene and carbon nanotubes), which maintain effective current conduction while preserving robust nanoscale performance [191].

**FIGURE 19:**
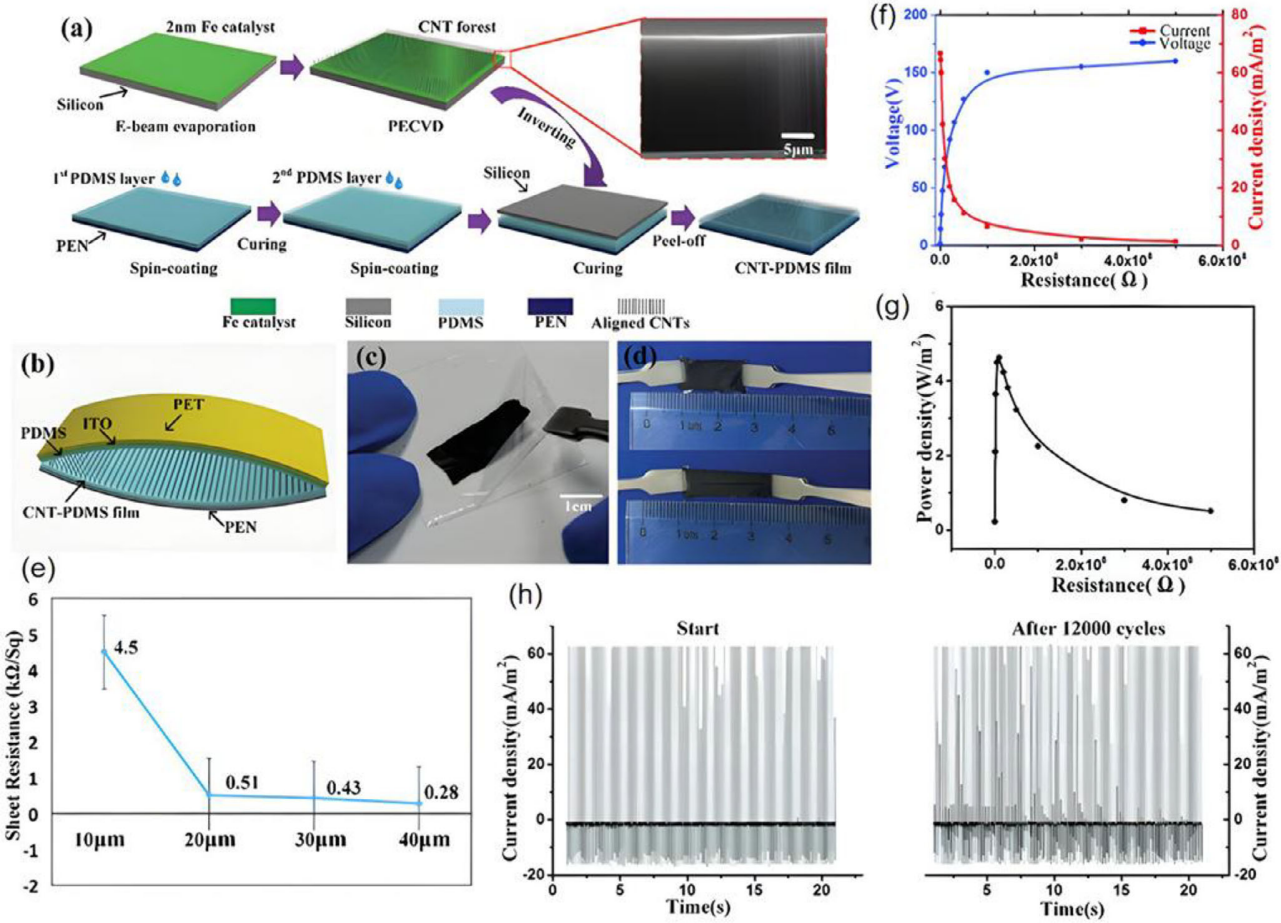
(a) Schematic diagram of the process for fabricating the aligned CNT–PDMS film. (b) Schematic diagram of the TENG. (c) Photographic image of the TENG. (d) An optical image of the stretchable aligned CNT–PDMS film. (e) The sheet resistance of the CNT–PDMS films with CNTs of different lengths. (f) Output voltage, current density, and (g) power density under different external loads. (h) Electrical stability tests of the TENG for 12 000 cycles. Reproduced with permission [193]. Copyright 2016, Royal Society of Chemistry.

The dielectric constant, which quantifies a material's charge‐storage capability, directly influences energy conversion efficiency. A higher dielectric constant increases charge storage, thereby enhancing the output voltage and current of nanogenerators. As illustrated in Figure 19a–d, researchers embedded aligned carbon nanotubes (CNTs) onto the surface of polydimethylsiloxane (PDMS) to create an effective electron‐donating dielectric layer. This composite layer not only boosts electron generation but also exhibits excellent stretchability [192].

Figure 19e–g demonstrates that the resulting CNT‐PDMS triboelectric nanogenerator (TENG) achieves an output voltage of 150 V and a current density of 60 mA/m^2^—improvements of 250% and 300%, respectively—compared to TENGs fabricated with directly doped PDMS/multi‐walled carbon nanotubes. Moreover, the aligned CNT‐PDMS film displays superhydrophobicity, with a contact angle of 154°, and a favorable sheet resistance of 280 Ω/sq [193]. The TENG maintains outstanding stability over cyclic testing exceeding 12,000 cycles (see Figure 19h), indicating that CNT doping significantly enhances the electrical properties and durability of the nanogenerator, thereby expanding its potential for long‐term energy harvesting and sensing applications.

Nanogenerators typically operate in dynamic environments, necessitating materials with exceptional mechanical properties, particularly flexibility, stretchability, and fatigue resistance. Prominent candidates include poly(dimethylsiloxane) (PDMS), poly(vinylidene fluoride) (PVDF), poly(vinyl alcohol)/poly(acrylic acid) (PVA/PAA) double‐network hydrogels, and gelatin‐methacryloyl (GelMA) hydrogels, which are often employed in flexible and stretchable energy‐harvesting systems [194]. These materials must adapt to various shapes and surfaces to suit different application scenarios. Flexible materials, such as polymers and nanocomposites, maintain their strength while offering excellent bending and stretching capabilities, making them ideal for wearable devices and smart textiles.

**FIGURE 20:**
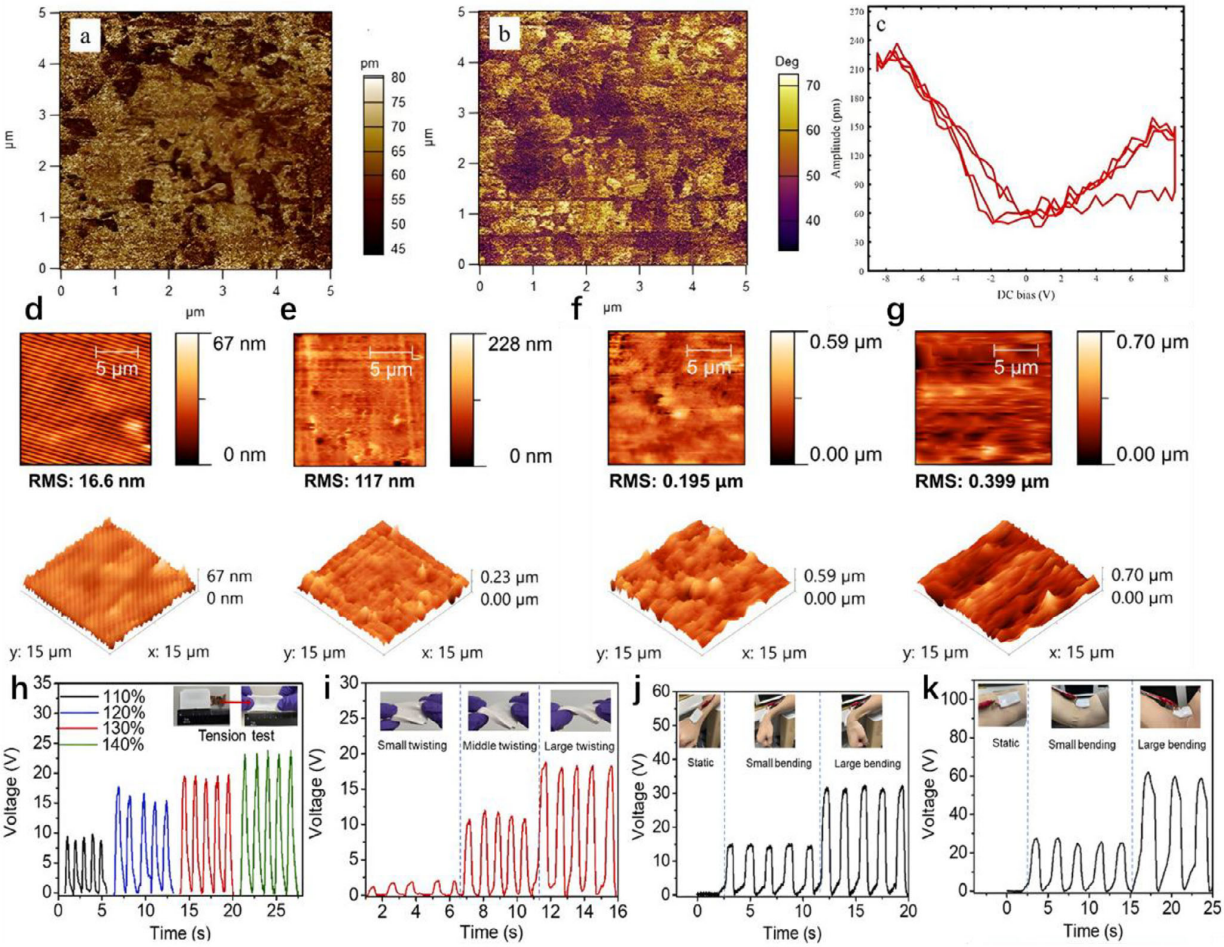
(a) and (b) represent the PFM amplitude and phase of PVDF film at an applied voltage of 1.5V, respectively, and (c) represents the butterfly loop obtained for the active material in the range ‐8V to +8V. Reproduced with permission [146]. Copyright 2020, Elsevier. AFM 2D and 3D images (d–g) of PDMS, S‐PDMS, SG‐PDMS, SG‐PDMS‐ I, and SG‐PDMS‐ II of composite films, respectively. Reproduced with permission [195]. Copyright 2020, MDPI. (h) Open‐circuit voltage measurement of the F‐MPPh TENG during a tensile test (110–140%). (i) The open‐circuit voltage of the F‐MPPh TENG under various degrees of twisting. (j) Open‐circuit voltage is recorded when the F‐MPPh TENG is bent downwards at different angles. (k) Open‐circuit voltage was observed with varying angles of upward bending of the F‐MPPh TENG. Reproduced with permission [196]. Copyright 2024, Elsevier.

Figure 20a–c shows the PFM amplitude and phase of the PVDF film under a voltage of 1.5 V, as well as the butterfly‐shaped loop of the active material obtained in the range of −8 V to +8 V, indicating that the PVDF film has excellent mechanical properties. For example, PDMS‐based triboelectric nanogenerators (TENGs) integrated with flexible sensors are conformally attached to the skin for real‐time monitoring of physiological signals, such as heart rate and respiration [195]. Figures 20d‐g show the two‐dimensional and three‐dimensional AFM surface images of each modified PDMS sample, with root‐mean‐square roughness (RMS roughness) provided for further analysis. In addition, researchers modified the surface of MXene nanosheets using 3‐chloropropyltrimethoxysilane (CPTMS) and perfluorooctyltriethoxysilane (FOTS) to prepare Cl‐MXene and F‐MXene hydrogel TENGs, named Cl‐MPPh and F‐MPPh TENGs, respectively. As shown in Figures 20h‐k, the F‐MPPh energy harvester exhibits distinct output voltages when the hand stretches its elongation from 110% to 140%, and the device generates different output voltage waveforms during wrist and elbow bending [196]. It indicates that materials such as PVDF, PDMS, CPTMS, and FOTS possess good flexibility and stretchability, and have promising application scenarios in the field of nanogenerators.

Moreover, nanogenerator materials must possess sufficient strength to withstand repeated mechanical stress and fatigue. Materials must resist environmental factors like temperature fluctuations, humidity, and chemical corrosion. In practical applications, materials may be exposed to corrosive environments, so selecting corrosion‐resistant materials—such as certain metal oxides and polymers—enhances the durability of the device. Materials should also maintain stable performance under extreme temperature conditions, adapting to diverse working environments. Recent studies have fabricated high‐temperature and corrosion‐resistant nanogenerators using nickel‐based superalloys, graphene/ceramic composites, zirconia (ZrO_2_), silicon carbide (SiC), and alumina (Al_2_O_3_), which demonstrate exceptional thermal stability (>1000°C) and corrosion resistance in harsh conditions [197].

The surface properties of materials significantly affect charge generation and transfer. Materials with high surface roughness or specialized surface structures can increase the contact area, facilitating charge separation and transfer—key factors in triboelectric generators. Recent studies have shown that introducing microscale groove structures on the surface of PDMS enhances the output voltage of nanogenerators by approximately 30% [198]. Furthermore, applying hydrophobic coatings on nanogenerator materials has improved their stability in high‐humidity environments, thereby extending their operational lifespan [199]. Through surface modification techniques like coating and doping, materials' electrical performance and chemical stability can be further optimized.

As sustainability becomes an increasingly important consideration, the environmental friendliness of nanogenerator materials must be prioritized. The material preparation process should minimize energy consumption and reduce environmental harm. Eco‐friendly synthesis methods—such as aqueous‐phase synthesis, sol–gel techniques, electrochemical synthesis, photochemical synthesis, and microwave‐assisted synthesis—play a vital role in sustainable development. These methods reduce toxic solvent use, cut energy consumption, and improve atom economy [200, 201].

The material preparation process should also be straightforward, reproducible, and scalable to meet the demands of large‐scale manufacturing. Choosing materials that are easy to synthesize through solution‐based approaches, vapor deposition, or mechanical grinding can significantly reduce production costs and enhance efficiency. For instance, through ligand‐regulated self‐assembly of CdSe quantum dots, flexible photovoltaic films with carrier mobility exceeding 10 cm^2^/V·s have been fabricated, cutting synthesis costs by 80% compared to conventional vapor deposition methods [202]. Additionally, solvothermal synthesis of ZIF‐8/graphene composites has resulted in a specific surface area greater than 2000 m^2^/g, making them highly efficient for mechanical energy harvesting applications [203].

Ensuring good reproducibility in the preparation process is crucial to maintaining product consistency. Materials should exhibit excellent processability, enabling them to be fabricated into desired shapes and structures that meet diverse application requirements. The versatility of nanogenerator materials is also of great significance. When materials can simultaneously provide energy‐harvesting and sensing functionalities, their potential applications expand dramatically. By integrating these multifunctional capabilities, nanogenerator materials can be utilized in advanced applications, including smart devices, the Internet of Things, and wearable technologies.

#### 3.3.2 Micro/Nano‐Fabrication Techniques

The fabrication techniques used for nanogenerators are broadly classified into top‐down and bottom‐up approaches. Top‐down methods involve the creation of nanogenerators from bulk materials, making them suitable for mass production (Table 7). These methods include nanolithography, anodizing, laser processing, and dry etching. Among these, nanolithography is based on the minimum size requirements of the nanogenerator, with a perfectly aligned mask over the wafer pattern. Lithography techniques can utilize light (optical lithography), electrons (electron‐beam lithography), ions (ion‐beam lithography), nanoimprint lithography, thermal imprinting lithography, or X‐rays (X‐ray lithography) [204].

**TABLE 7:** Classification of micro/nano‐fabrication techniques.

Types of fabrication techniques	Technical principle	Advantages	Disadvantages	Refs.
Top‐down approaches				
Nanoimprint lithography	Micro‐nano templates are imprinted on substrates, followed by curing/demolding to form micro‐nano structures.	High resolution; simple process and low cost; great mass production potential and high efficiency; compatibility with various substrates.	Difficult template fabrication and easy abrasion; high difficulty in controlling the uniformity of large‐area patterns; strict requirements for substrate flatness.	[205]
Thermal nanoimprint lithography	The substrate is softened by heating, imprinted with a template, and then cooled and demolded to form micro‐nano structures.	High pattern fidelity and structural stability; compatibility with a variety of thermoplastic photoresists and rigid substrates.	Requires a heating‐cooling cycle; long process period and low efficiency; incompatibility with heat‐sensitive substrates and materials.	[206]
Extreme ultraviolet lithography	13.5 nm extreme ultraviolet (EUV) light exposes the photoresist, transferring micro‐nano patterns through a mask.	Ultra‐high resolution; enables the fabrication of more miniaturized and high‐performance semiconductor chips.	Extremely high manufacturing and maintenance costs; stringent requirements for the performance of specialized EUV photoresists.	[207]
Anodic oxidation	A metal is used as the anode and is electrified in an electrolyte, forming a dense oxide film on the metal surface.	Strong adhesion and excellent corrosion resistance of the oxide film; simple process, low cost, and easy mass production.	Limited range of applicable metals; high brittleness of the film layer, which is prone to cracking upon impact.	[208]
Femtosecond laser processing	A femtosecond ultra‐short pulse laser is focused on the material surface, and micro‐nano structure processing is achieved through nonlinear absorption.	Minimal heat‐affected zone and high processing precision; capability of fabricating complex micro‐nano structures.	High equipment cost; stringent requirements for focusing accuracy and pulse parameter control.	[209]
Dry etching	Micro‐nano structures are formed by etching materials via physical bombardment or chemical reactions using plasma/reactive gases.	High etching precision and excellent anisotropy; no waste liquid pollution.	High equipment cost and complex process; relatively low etching rate.	[210]
Bottom‐up approaches				
Sol–gel processing	Precursors undergo hydrolysis and condensation to form sol, which is then gelated and subjected to drying/sintering for material preparation.	Low‐temperature process with low energy consumption; uniform composition and precise doping.	Prone to shrinkage and cracking during drying; long preparation cycle.	[211]
Laser pyrolysis	Laser heats reactants for rapid pyrolysis to prepare nanomaterials.	Fast heating rate, high product purity, and uniform particle size; strong process controllability, no need for complex post‐treatment.	High cost of laser equipment; difficulty in large‐scale mass production.	[212]
Chemical vapor deposition	Gaseous precursors undergo chemical reactions on the substrate surface to deposit and form thin films/materials.	Uniform thin films with strong adhesion; a wide range of applicable materials (metals, ceramics, semiconductors, etc.).	Requires a high‐temperature/vacuum environment and high equipment cost; relatively low deposition rate.	[213]
Aqueous Chemical Growth	In aqueous media, precursors react and grow on the substrate surface to form materials.	Low‐temperature and mild process with low energy consumption; low cost, simple process, and easy scaling‐up.	Relatively low product crystallinity; moderate adhesion and sensitivity to reaction parameters (pH, temperature).	[214]
The hydrothermal process	Under sealed high‐pressure conditions, with water as the medium, precursors react/crystallize to prepare materials.	High product crystallinity and purity; mild process with easy regulation of morphology/particle size.	Requires high‐pressure reactors, leading to high equipment cost; long reaction cycle and difficulty in large‐scale mass production.	[215]
Chemical Bath Deposition	In aqueous solution, precursors undergo chemical reactions to deposit and form thin films on the substrate surface.	Simple process, low cost, and easy for large‐area preparation; low‐temperature operation without complex equipment.	Slow deposition rate and relatively thin films; moderate crystallinity and limited adhesion.	[216]

**FIGURE 21:**
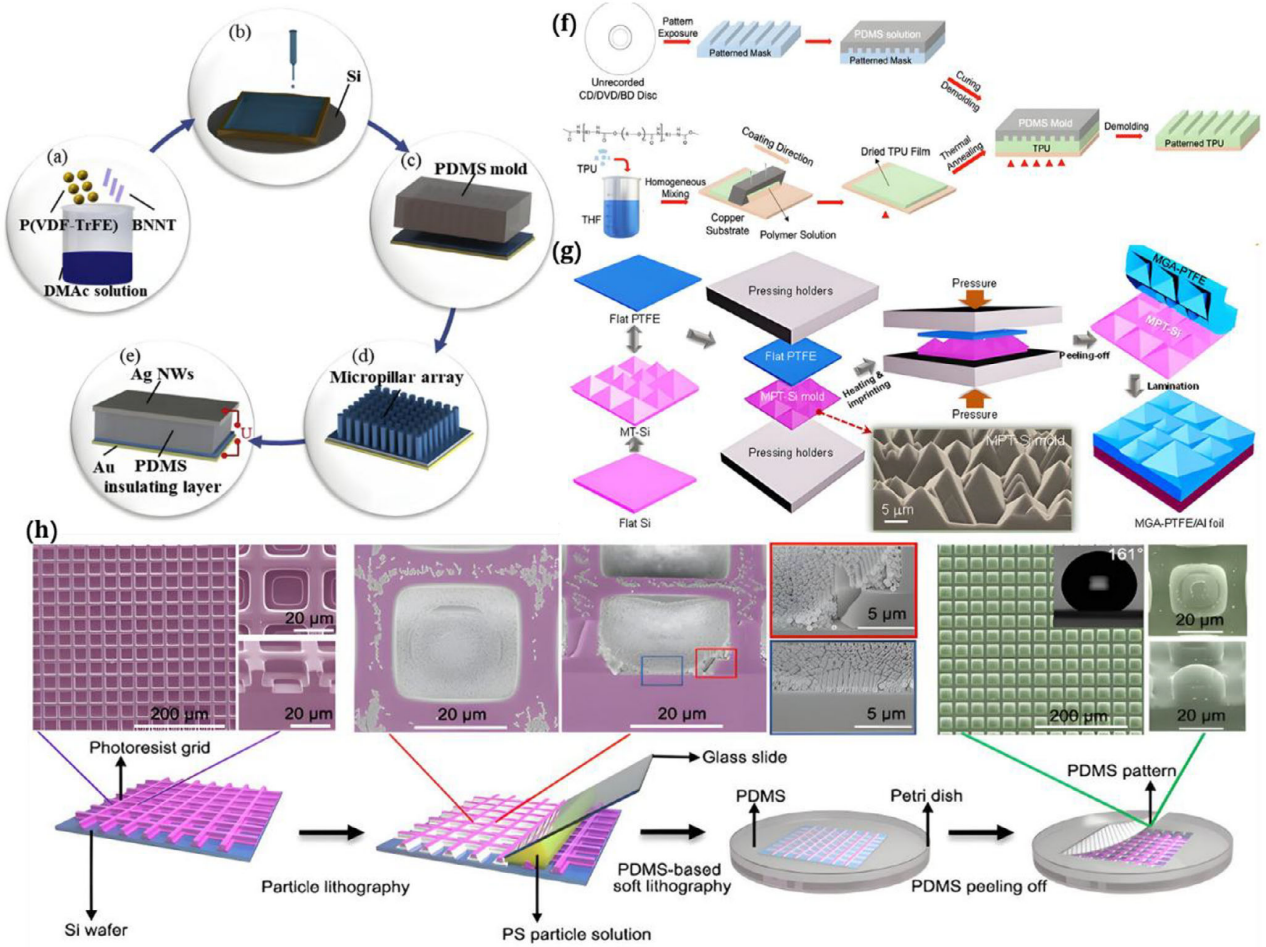
(a‐e) Experimental processes used for the fabrication of a piezoelectric nanogenerator based on P(VDF‐TrFE)/BNNTs nanocomposite micropillar array. Reproduced with permission [205]. Copyright 2019, Elsevier. (f) Schematic process of preparing PTPU film on a copper substrate through the doctor blade method and capillary force lithography. Reproduced with permission [217]. Copyright 2023, Elsevier. (g) Fabrication process of MGAPTFE by TIL technique via an MPTSi mold by an anisotropic chemical etching process. Reproduced with permission [206]. Copyright 2018, American Chemical Society. (h) Schematic of the fabrication process of a 3D hierarchical PDMS interlayer via particle lithography. Reproduced with permission [218]. Copyright 2019, Elsevier.

Nanoimprint lithography (NIL) is a technology for achieving nanoscale pattern transfer through mechanical imprinting, with the core being the replication of high‐precision nanostructures on a substrate using a template [205]. Based on this technology, researchers have fabricated a novel piezoelectric nanogenerator (PENG) with a nano‐composite micropillar array of P(VDF‐TrFE)/boron nitride nanotubes (BNNTs), demonstrating enhanced performance and excellent neutron radiation shielding properties (see Figure 21a–e).

Thermal nanoimprint lithography transfers nanoscale patterns from a template to a substrate through a “heating‐imprinting‐cooling” process [206]. This technology is used to enhance the performance of microstructure‐based PTFE triboelectric nanogenerators for self‐powered electronic products (see Figure 21g). The introduction of extreme ultraviolet lithography (EUV) with a 13.5 nm wavelength has enabled the fabrication of interdigitated electrodes with a linewidth of 50 nm, significantly increasing the triboelectric nanogenerator's output charge density to 3.5 µC/m^2^—double that of conventional photolithography [207]. Additionally, combining stepper photolithography with reactive ion etching (RIE) has facilitated the creation of 3D pyramidal arrays on silicon substrates, achieving a 25% energy conversion efficiency in piezo‐triboelectric hybrid devices [219]. Nanogenerators fabricated using electron beam lithography (EBL), which enables site‐specific growth of InAs quantum dots (<20 nm spacing) on GaAs substrates, demonstrate single‐electron tunneling effects [220].

Researchers designed an antibacterial flexible triboelectric nanogenerator by precisely controlling the geometric shape of nanostructures through adjusting the curing conditions of capillary force lithography (see Figure 21f) [217]. For example, when fabricating an antibacterial flexible nanogenerator, an appropriate structure can be shaped as designed to optimize the power generation performance. Additionally, it can pattern thermoplastic polyurethane (TPU) films at the nanoscale, preventing biofilm formation without adding chemical antibacterial agents and thus meeting the hygiene requirements of wearable devices and other applications [221].

Particle lithography is an advanced patterning technique that leverages interactions between energetic particles (e.g., electrons, ions) and materials to fabricate nanoscale features [218]. Building on this technology, researchers have synthesized a three‐dimensional (3D) hierarchically porous superhydrophobic interlayer using particle lithography, enabling the fabrication of triboelectric nanogenerators (TENGs) with exceptional humidity resistance and anti‐fouling properties at the device interface (see Figure 21g).

**FIGURE 22:**
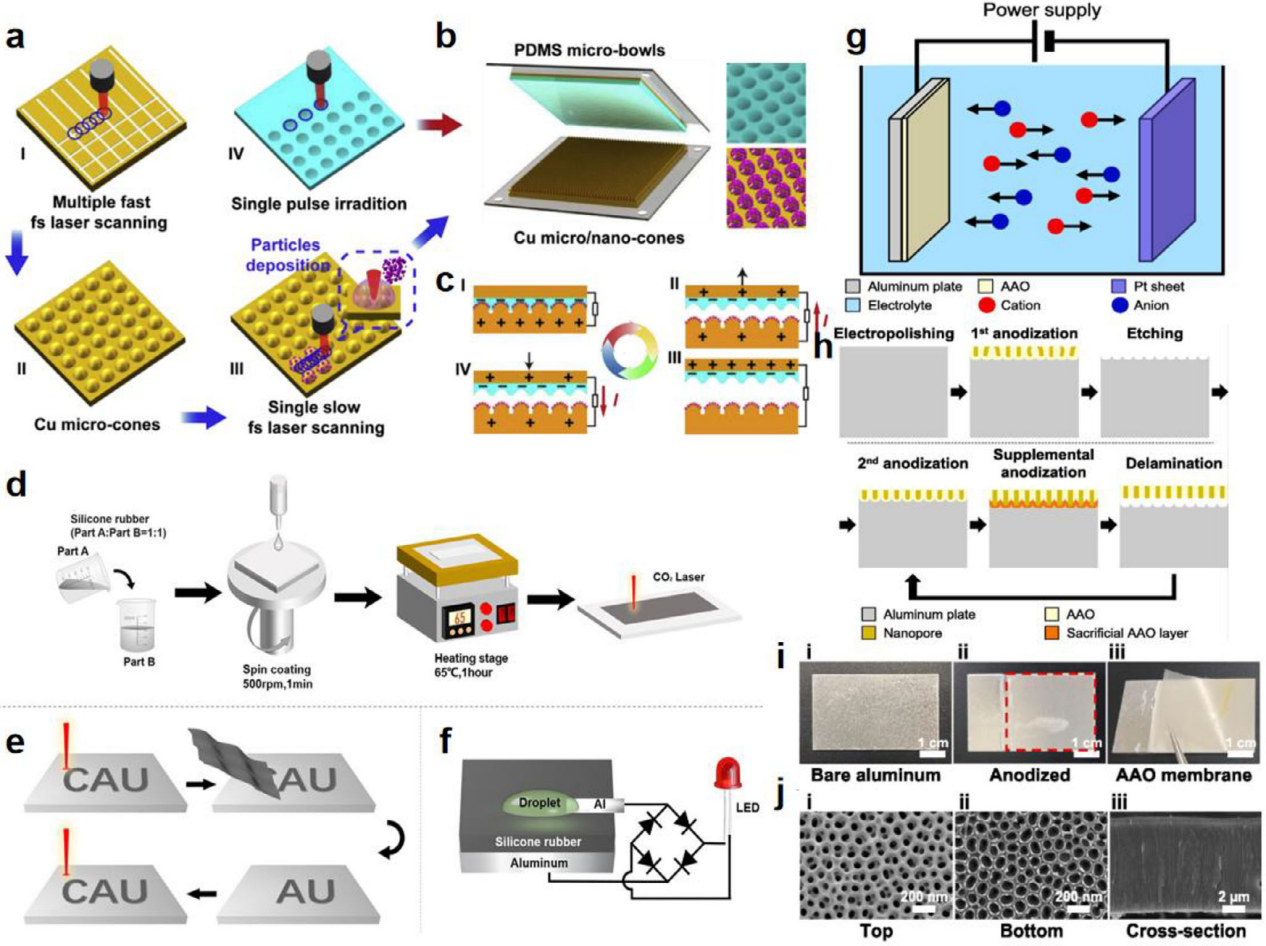
(a) Femtosecond laser direct writing processes for preparing micro/nano structures on surfaces of Cu and PDMS films, respectively. (b) Fabricated TENG based on Cu film with micro/nano‐cones structure and PDMS film with micro‐bowls structure. (c) Working principle of the TENG in contact‐separation mode. Reproduced with permission [228]. Copyright 2019, Elsevier. (d) Schematic illustration of the whole experiment method. (e) Laser recovery of worn‐out superhydrophobic letter “C.” (f) The structure of the TENG device. Reproduced with permission [229]. Copyright 2022, Frontiers Media S.A. (g) Schematic of anodization process setup. (h) Series of processes used AAO membranes from an aluminum plate. After all the processes, the continuous repeat process from second anodization to delamination results in the sustained productivity of the AAO membranes. (i) Appearance in the aluminum plate and AAO membrane during the main step of the AAO membrane fabrication process. (j) SEM images of the top, bottom, and cross‐section of the AAO membrane. Reproduced with permission [222]. Copyright 2024, Elsevier.

Anodic oxidation is an unbalanced process that occurs in two phases (see Figure 22g–j): during the voltage rise, there is rapid expansion and rearrangement of the pores, with the oxidation rate exceeding the dissolution rate [208]. In the subsequent constant voltage anodization, the oxidation and dissolution rates approach one another due to the thickening of the barrier layer, resulting in the growth of a thin, uniform oxide barrier on the metal [222].

However, anodized oxide layers exhibit high brittleness and therefore need to be integrated with flexible substrates (e.g., PDMS, PET) [223]. Furthermore, the low conductivity of oxides necessitates the inclusion of conductive layers (e.g., graphene, Ag nanowires) [224]. Dry etching, which operates in the gas phase without an aqueous solution, requires a reliable etch mask to remove the material [210]. Recent studies have demonstrated the effectiveness of dry etching in improving nanogenerator performance. For example, Deep Reactive Ion Etching (DRIE) was used to fabricate silicon micropillar arrays with an aspect ratio of 20:1 (5 µm diameter, 100 µm height) on silicon substrates. After polydimethylsiloxane (PDMS) deposition, the triboelectric nanogenerator (TENG) achieved an output power density of 12.5 W/m^2^—six times higher than that of planar configurations [225]. Additionally, plasma etching was used to create TiO_2_ grating structures (500 nm periodicity) on titanium dioxide films, achieving a light absorption rate of 95% [226]. These films were integrated into a hybrid piezo‐triboelectric generator driven by light, yielding an energy conversion efficiency of 18.3% [227].

Femtosecond lasers enable the direct fabrication of micro/nanostructure‐enhanced triboelectric nanogenerators (TENGs) on two triboelectric layers [209]. Using laser scanning ablation technology, researchers created strip‐shaped and cone‐shaped micro/nano dual‐scale structures on the surface of copper (Cu) films. Additionally, microbowl structures of varying sizes were fabricated on polydimethylsiloxane (PDMS) surfaces through single‐pulse laser irradiation (see Figure 22a‐c). These nanostructures enhance the piezoelectric polarization effect due to their aligned morphology and increased surface‐to‐volume ratio [228]. Additionally, excimer laser ablation has been used to create high‐density electrode arrays on flexible substrates such as silicone rubber, improving both mechanical flexibility and electrical output performance (see Figure 22d‐f). Laser processing technologies provide high accuracy, fast response times, non‐contact capabilities, and excellent operability [230]. These techniques offer distinct advantages in enhancing the performance and mechanical stability of nanogenerators, though they also face technical challenges. Future research should focus on integrating the strengths of these approaches, optimizing process parameters, reducing manufacturing costs, and improving scalability to advance the development of high‐performance, mechanically robust, and flexible nanogenerators.

Bottom‐up methods involve the self‐assembly of atoms or molecules through chemical reactions to synthesize nanoparticles. Sol–gel processing is commonly used to produce inorganic oxide materials at low temperatures via wet chemistry, resulting in hydrophobic membranes resistant to water and offering advantages such as low processing temperatures, good molecular properties, homogeneity, and low equipment costs [211].

**FIGURE 23:**
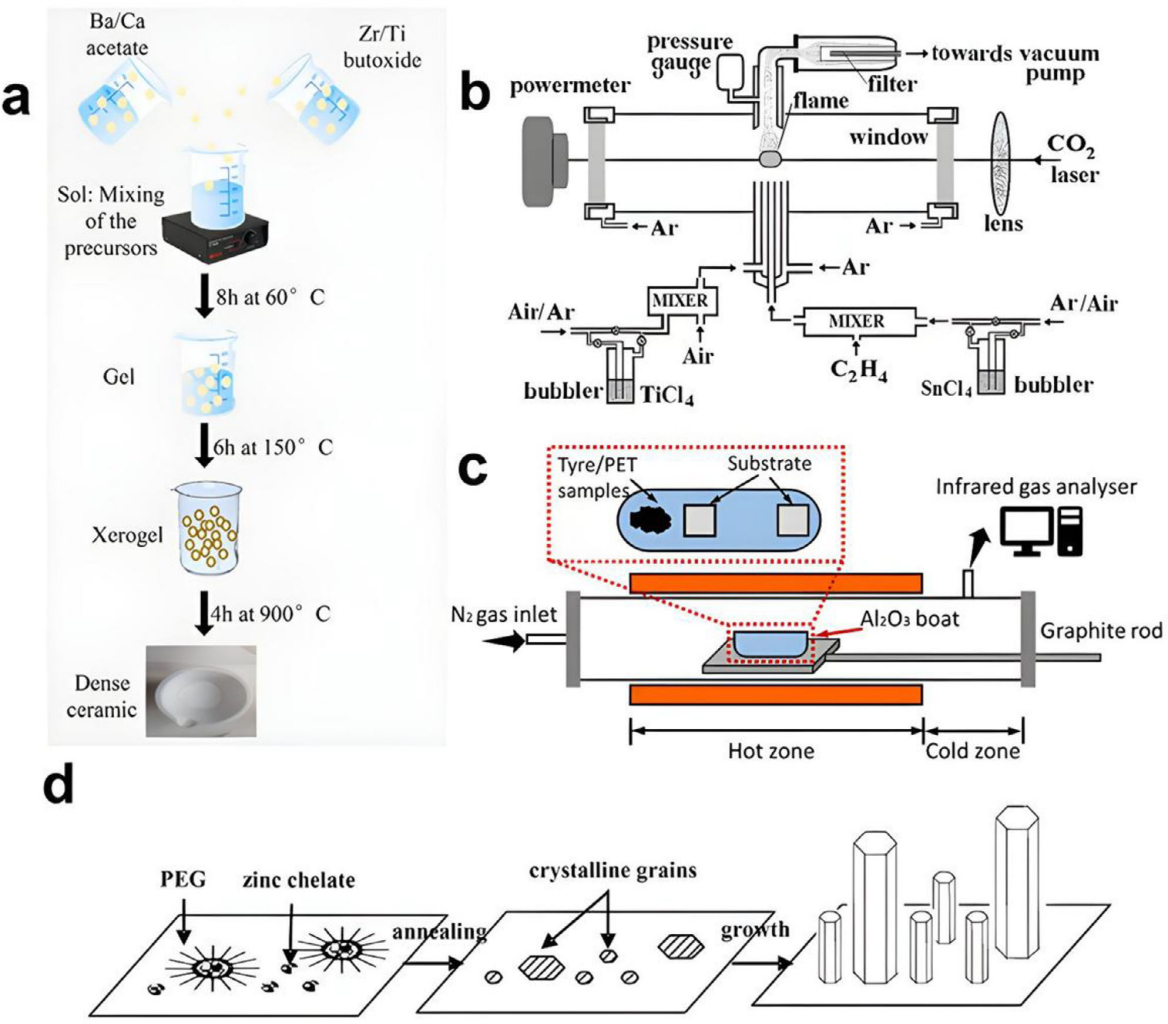
(a) Sol–gel for the synthesis of the BZT and BCZT nanoceramics. Reproduced with permission [232]. Copyright 2022, Elsevier. (b) Experimental setup for the synthesis of TiO_2_/SnO_2_ nanoparticles by laser pyrolysis. Reproduced with permission [235]. Copyright 2017, Elsevier. (c) Schematic of Green‐Chemical Vapor Deposition (G‐CVD) system. Reproduced with permission [238]. Copyright 2017, Royal Society of Chemistry. (d) Growth schematic diagram of the ZnO nano‐micro‐crystals grown on seed layers controlled by PEG assistant. Reproduced with permission [241]. Copyright 2006, Elsevier.

Recent studies have explored the sol–gel process for fabricating nanogenerators. For example, polyimide (PI) films were prepared using the sol–gel method for the triboelectric layer of triboelectric nanogenerators (TENGs). Optimizing sol pH and drying temperature resulted in PI films with increased surface roughness, significantly enhancing TENG performance [231]. In another study, the sol–gel technique was employed to synthesize lead‐free piezoelectric ceramic (Ba, Ca)(Zr, Ti)O_3_, which enhances the performance of flexible lead‐free nanogenerators (see Figure 23a). A maximum output voltage of 4.55 V was achieved, underscoring the potential of sol–gel processing in advancing the performance of nanogenerators [232].

Laser pyrolysis, which uses infrared lasers to decompose substances in an oxygen‐deficient environment, is capable of producing high‐purity spherical nanoparticles with small diameters and minimal agglomeration [212]. For instance, CO_2_ laser pyrolysis of Zn(CH_3_COO)_2_ precursors in an argon atmosphere has been used to produce ZnO nanowires. High‐temperature transient reactions promote the preferential growth of ZnO crystals, reducing grain boundary defects and enhancing piezoelectric polarization [233]. In another study, laser pyrolysis of Bi_2_Te_3_ precursors was used to fabricate Bi_2_Te_3_ nanosheets (∼10 nm thick), which were then assembled into porous thermoelectric films. The porous structure improved the Seebeck coefficient, enhancing thermoelectric performance [234]. Additionally, researchers synthesized TiO_2_/SnO_2_ nanocomposites via laser pyrolysis using volatile TiCl_4_ and SnCl_4_ precursors (see Figure 23b) [235]. The resulting materials exhibited lower bandgap energies, and some compositions (containing 1.8 or 4.8 at.% Sn) demonstrated enhanced photoactivity during the UV‐induced discoloration of methyl orange solutions [236].

Chemical vapor deposition (CVD) is another technique used in nanogenerator fabrication. In this process, carbon nanotubes are grown in a horizontal quartz tube to design nanogenerators, producing stable, high‐quality carbon nanotubes [237]. Low‐pressure CVD (LPCVD) has been used to grow single‐layer graphene on copper foil, which is then transferred to a polydimethylsiloxane (PDMS) substrate as the triboelectric layer of a TENG [213]. The green chemical vapor deposition (G‐CVD) method transforms waste into functional materials of various forms by condensing waste‐generated gases onto selected substrates (see Figure 23c) [238]. Alternatively, plasma‐enhanced CVD (PECVD) has been used to grow zinc oxide (ZnO) nanorod arrays on silicon substrates at low temperatures, enabling high‐performance piezoelectric nanogenerators, expanding the application of CVD techniques in flexible electronics [239].

Aqueous Chemical Growth (ACG) is a simple, low‐cost, low‐temperature process that synthesizes well‐aligned, controllable nanostructures with low energy consumption and hazard levels [214]. By regulating the molar ratio of zinc nitrate to hexamethylenetetramine (HMTA), ACG precisely controls the aspect ratio and orientation of ZnO nanowires, offering a scalable production method for wearable TENGs [240]. Special plate‐like zinc oxide crystals and well‐aligned zinc oxide nano/micro composite rod arrays were synthesized on zinc oxide seed glass substrates by the aqueous chemical growth method (ACG) (see Figure 23d). Polyethylene glycol (PEG) was added to the seed precursor solution to obtain seed layers with different textures, on which nano/micro composite rods were formed, providing an effective strategy for synthesizing high‐performance zinc oxide nanogenerators [241].

**FIGURE 24:**
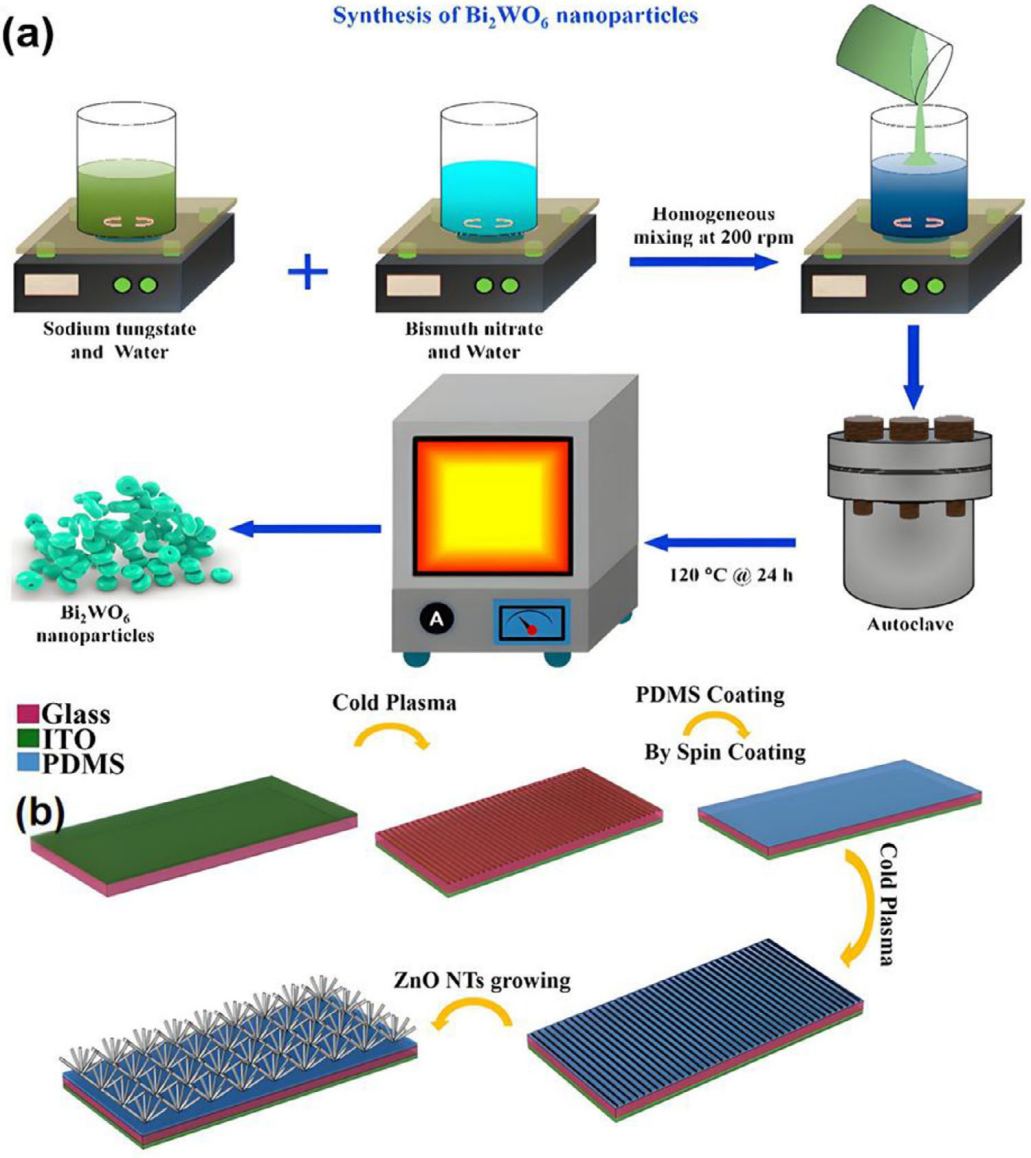
(a) The Chemical Bath Deposition Synthesis process of Bi_2_WO6 nanoparticles. Reproduced with permission [243]. Copyright 2022, American Chemical Society. (b) Schematic of S‐TENG preparation and ZnO NTs growing on the PDMS layer. Reproduced with permission [244]. Copyright 2023, Elsevier.

The hydrothermal process is a low‐cost, mass‐production method that yields flexible substrates and high‐crystallinity nanogenerators at low temperatures (around 100°C) [215]. For example, hydrothermal synthesis has been used to produce TiO_2_ nanotube arrays (∼50 nm pore size) for TENG triboelectric layers [242]. Additionally, SnSe nanosheets prepared via hydrothermal synthesis have demonstrated a thermoelectric figure of merit (ZT) of 1.8, representing a 120% improvement over bulk SnSe. Double‐concave Bi_2_WO_6_ nanoparticles with an orthorhombic (pseudo‐tetragonal) structure were synthesized via a simple and low‐cost hydrothermal method (see Figure 24a). Under the application of low vertical compressive force (0.15 kgf) without electrode polarization, stable and high output voltage (50 V) and current density (0.6 µA/cm^2^) were achieved, respectively [243].

Chemical Bath Deposition (CBD) is a solution‐based synthesis technique used for depositing thin films or nanostructures on substrates by controlling chemical reactions in precursor solutions [216]. Researchers employed ZnO nanotubes (ZnO NTs) to modify the surface through a cost‐effective and straightforward chemical bath deposition (CBD) method (see Figure 24b). The ZnO NTs increased surface roughness and provided a suitable pathway for electron transitions [244]. For example, CBD has been employed to deposit CuS nanosheets on cotton fabric, enhancing electron trapping capabilities through sulfur vacancies and rough surface morphology. This method's solution‐wetting properties make it ideal for producing uniform coatings on porous or fibrous substrates, such as textiles, making it suitable for large‐scale production of wearable TENGs [245].

Bottom‐up technologies provide a highly adaptable, cost‐effective manufacturing pathway for nanogenerators through molecular‐level assembly. This approach has advanced self‐powered sensing technologies, such as wearable energy devices, self‐powered sensor networks, and biomedical applications. By leveraging material innovations, process optimization, and interdisciplinary collaboration, these methods are poised to overcome challenges related to energy density, stability, and cost. In the future, bottom‐up techniques are expected to be a core technology in distributed energy systems, intelligent sensing, and green electronics, accelerating the transition toward self‐sustainability and intelligent systems.

#### 3.3.3 Surface Modifications for Biocompatibility

The biocompatible surface modification of nanogenerators can be approached from various angles, including chemical modification, biomolecule functionalization, nanostructure optimization, antibacterial property enhancement, and surface coating techniques. The selection of biodegradable materials with excellent biocompatibility, such as polylactic acid (PLA) and polyvinyl alcohol (PVA), followed by surface chemical modifications, such as introducing functional groups like hydroxyl and amino groups, can improve the interaction between the material and the biological system while minimizing immune responses. For example, the chemical modification of nanogenerators with functional groups like hydroxyl (─OH) and amino (─NH_2_) has demonstrated significant improvements in biocompatibility, without compromising their electromechanical performance [246].

**FIGURE 25:**
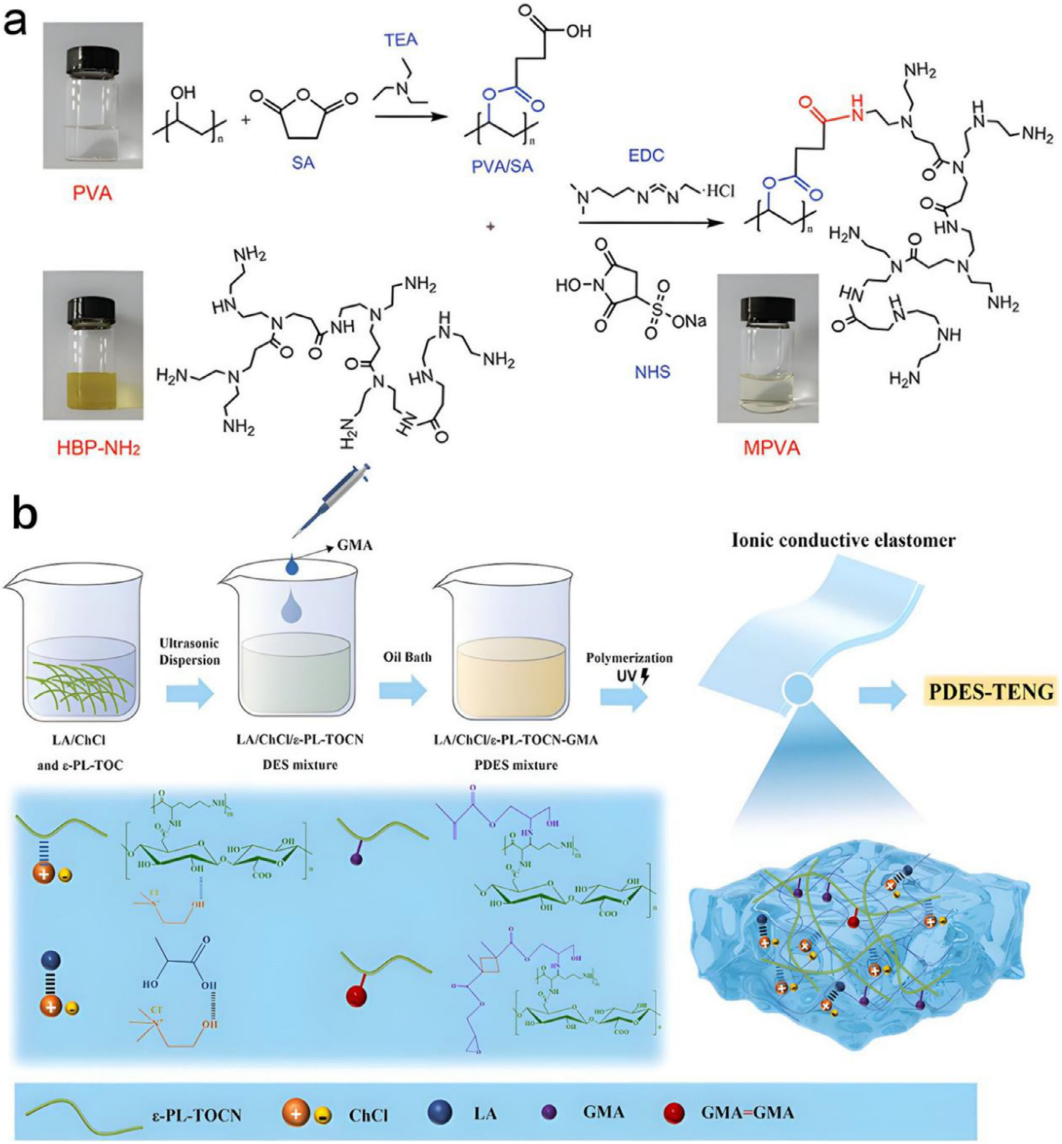
(a) Synthesis process of MPVA. Reproduced with permission [248]. Copyright 2022, Wiley. (b) Schematic illustration of the fabrication process and the crosslinking structure of the ε‐PL‐TOCN /PDES‐based ionic conductive elastomer. Reproduced with permission [251]. Copyright 2025, Elsevier.

For instance, APTES‐modified PVDF‐based triboelectric nanogenerators (TENGs) exhibited improved hemocompatibility and cell adhesion. The formation of an aminosilane layer on PVDF surfaces enhanced interfacial adhesion through hydrogen bonding, extending the activated partial thromboplastin time (APTT) from 80 s (unmodified) to 130 s [247]. Additionally, polyvinyl alcohol (PVA) was modified with an amino‐terminated hyperbranched polymer (HBP‐NH_2_) to develop a spinnable elastic polymer (MPVA). A knot‐reinforced electrospun nanofiber membrane (MPVA) with a lower Young's modulus and improved stretchability was demonstrated (see Figure 25a). It exhibits a robust network structure, tunable breathability, and high biocompatibility [248]. The strategic balance between biological safety and electromechanical efficiency positions these chemical modifications as a promising approach for advancing implantable and wearable nanogenerator technologies [249].

For instance, surface treatment of ZnO nanowires with 3‐aminopropyltriethoxysilane (APTES), which undergoes hydrolysis to form silanol (Si‐OH) groups that condense with surface hydroxyl groups on ZnO, resulted in enhanced biocompatibility. This modification increased the viability of human umbilical vein endothelial cells (HUVECs) from 70% to 92% [250]. Researchers incorporated glycidyl methacrylate (GMA) into a deep eutectic solvent (DES) matrix dispersed with ε‐PL‐tunicate cellulose nanofibrils (ε‐PL‐TOCN), promoting in‐situ polymerization through ring‐opening reactions between the GMA epoxides and both ε‐PL‐TOCN and the DES. The ε‐PL‐TOCN forms a dense hydrogen‐bond network, enhancing biocompatibility (see Figure 25b).

Moreover, biomolecule modification, such as the attachment of peptides, saccharides, and proteins, on the material surface can further promote cell attachment and proliferation. For example, coating with collagen or fibronectin can enhance the interaction between cells and materials, facilitating cell growth and tissue regeneration. Peptide functionalization mimics extracellular matrix (ECM) components, thereby promoting cell adhesion and tissue integration. For instance, covalent bonding between peptide chains and functional groups (e.g., carboxyl or amino groups) or self‐assembly via hydrophobic/electrostatic interactions can anchor peptides onto material surfaces. A study demonstrated that RGD peptide‐modified ZnO nanowires significantly improved fibroblast and endothelial cell adhesion while reducing macrophage activation and pro‐inflammatory cytokine secretion [252]. Similarly, carbohydrate‐based modifications, such as grafting hyaluronic acid (HA) or chitosan via chemical conjugation or physical adsorption, mimic cell‐surface glycoprotein structures to minimize immune recognition and nonspecific adsorption [253, 254]. Enzymatic surface glycosylation further reduces immune cell responses. For example, HA‐coated polydimethylsiloxane (PDMS)‐based TENGs exhibited a hemolysis rate below 2%, in compliance with ISO 10993 standards.

Protein functionalization directly leverages biomolecular activities to enhance biocompatibility. Physical adsorption (via surface charge/hydrophobicity) or covalent immobilization (using crosslinkers like EDC/NHS) enables specific biofunctions. Fibronectin (FN) coatings promote angiogenesis and tissue regeneration, while heparin modifications suppress platelet adhesion and coagulation. These biomolecular strategies synergistically improve biocompatibility by emulating biological microenvironments and introducing tailored bioactivities, without compromising energy‐harvesting efficiency [255, 256]. Future advancements in surface engineering, combined with novel functional molecules, are expected to expand nanogenerator applications in implantable medical devices, wearable electronics, and intelligent sensing systems, driving innovation in biomedical technologies.

**FIGURE 26:**
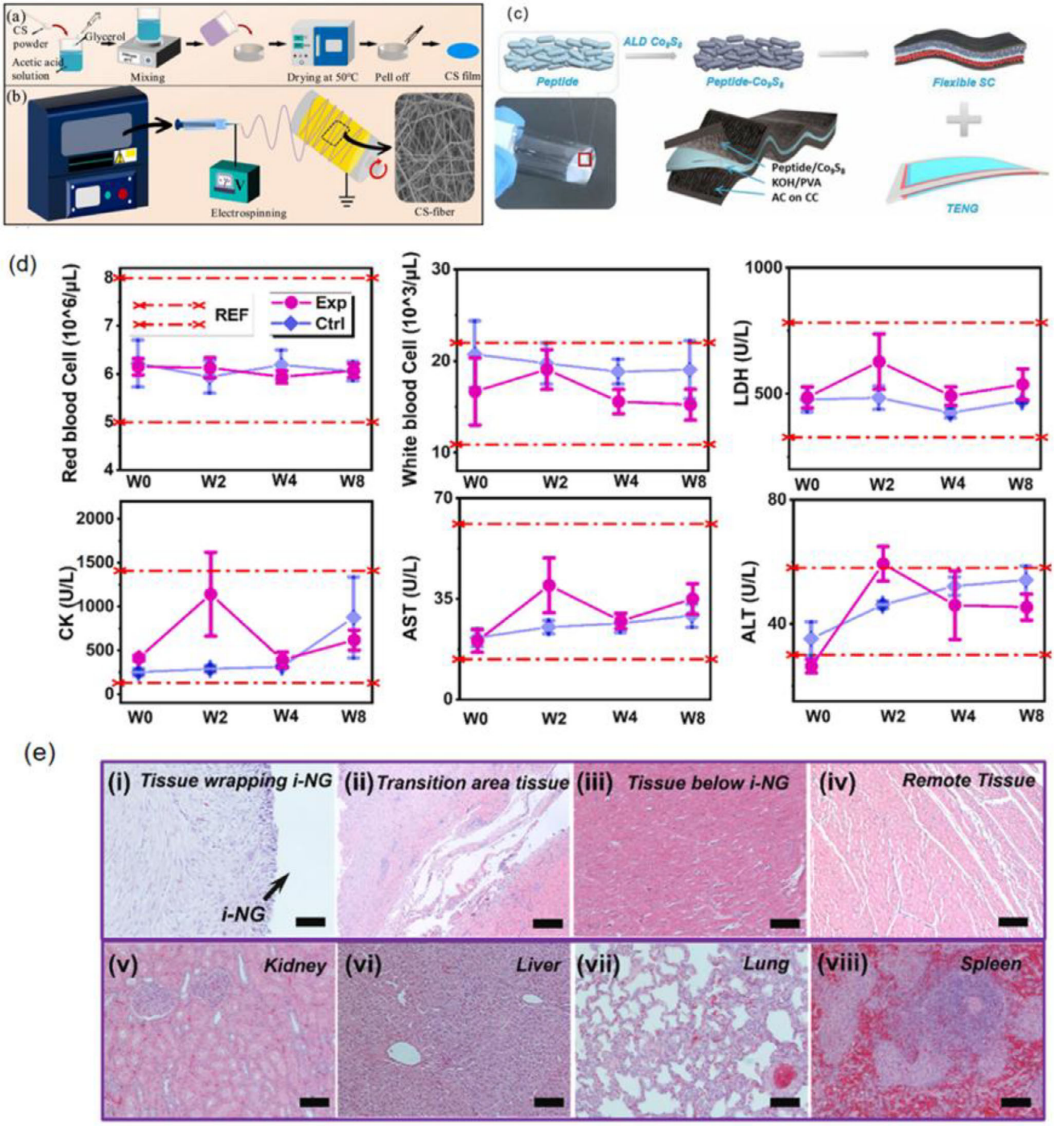
(a) The preparation process of CS film. (b) The process of preparing CS‐fiber film by electrospinning. Reproduced with permission [257]. Copyright 2024, Elsevier. (c) Preparation for peptide‐Co9S8 core‐shell nanostructures and TENG/SC system. Reproduced with permission [258]. Copyright 2019, Elsevier. (d) Blood and serum test results. Experiment group (N  =  4) are the pigs implanted with i‐NGs. Control group (N  =  4) are the pigs without i‐NG implantation. The normal range is marked in between two orange dashed lines. Red blood cell (RBC) and white blood cell (WBC) are indicators of hematopoietic function and infection, respectively. Alanine aminotransferase (ALT), aspartate aminotransferase (AST), creatine kinase (CK), and lactate dehydrogenase (LDH) are the common cardiac enzymes to evaluate heart function. (e) pathological analyses by H&E staining of regional tissues surrounding the implanted device (i)‐(iv) and on most vital organs, such as (v) kidney, (vi) liver, (vii) lung, and (viii) spleen. Reproduced with permission [259]. Copyright 2021, Elsevier.

To enhance the biocompatibility of triboelectric nanogenerators (TENGs), researchers have proposed a surface modification approach involving either replicating the surface topography of sandpaper to create microstructures on chitosan films or using electrospinning to construct porous structures (see Figure 26a,b). These methods significantly increase the effective contact area between the two triboelectric materials. The output performance of a vertical contact‐separation TENG using chitosan (positive) and fluorinated ethylene propylene (FEP, negative) films as triboelectric materials is greatly enhanced [257]. Nanostructured peptides possess inherent flexibility and biocompatibility. A core–shell structure of peptide–Co_9_S_8_ nanobricks was synthesized by conformally coating a thin shell of Co_9_S_8_ (see Figure 26c). The flexible asymmetric supercapacitor can also be coupled with a triboelectric nanogenerator (TENG) to provide a flexible self‐powered TENG/SC system with good biocompatibility [258].

In addition, adjusting the nanostructure of the material, such as surface roughness and porosity, can improve cell adhesion and growth. Surface roughness directly influences biocompatibility by regulating cell adhesion, spreading, and signal transduction. Electrospun PVDF nanofibers with rough surfaces (*R*_a_ = 30 nm) significantly reduce platelet adhesion density by 70%, thereby enhancing hemocompatibility [260]. A three‐dimensional porous PDMS structure (pore size 10–50 µm, porosity 65%), constructed via the salt‐template method, doubles vascular density while maintaining stable energy output [261]. By precisely controlling surface roughness and porosity, the biocompatibility of nanogenerators is significantly enhanced while preserving high‐efficiency energy harvesting capabilities.

Surface coating techniques, such as polymer coating and silanization, can provide a protective layer to prevent direct contact between the material and the biological system. A polyethylene glycol (PEG) coating reduced the hemolysis rate of TENGs from 5% to 1% [262]. Polydopamine (PDA) coatings enhanced NIH‐3T3 fibroblast adhesion density by threefold [263]. Chitosan coatings suppressed Staphylococcus aureus proliferation (antibacterial rate >99%) and increased keratinocyte migration speed by 50% [264]. Polymer coatings and silanization techniques significantly improved the hemocompatibility, cytocompatibility, and anti‐infection capabilities of nanogenerators by tailoring surface chemistry, while ultrathin or conductive coating designs preserved energy‐harvesting efficiency. The modified nanogenerator, when implanted in a pig's heart, can operate stably in vivo for a long term, with its hematopoietic function and cardiac function remaining unchanged (see Figure 26d‐e). Pathological analysis of the tissues in the area around the implanted device and other vital organs via H&E staining shows that the implanted nanogenerator causes minimal damage to the surrounding areas and organs [259]. Future efforts should focus on optimizing coating stability and multifunctionality to advance practical applications in implantable medical devices.

#### 3.3.4 Integration with Neuro‐Immune Interfaces

The integration of nanogenerators with the neuro‐immune interface offers vast potential for applications in energy harvesting, signal transmission and regulation, sensing, adaptive functions, and multi‐functional integration. This integration not only provides novel tools for fundamental research but also paves the way for groundbreaking advancements in future biomedical applications.

Nanogenerators can efficiently harvest energy from various human body functions, such as breathing, heartbeat, pulse, and concentration gradients, converting it into electricity. This capability allows them to supply continuous power to micro‐sensors and stimulation devices at the neuro‐immune interface, eliminating the need for external power sources. Implantable ZnO piezoelectric nanogenerators, for example, utilize mechanical deformations from heartbeats or respiration to generate electrical pulses (∼100 mV), which have been used to stimulate sciatic nerve regeneration, improving motor function recovery by 40% in rats [16]. Additionally, implantable nanogenerators harvest abdominal pressure fluctuations to drive vagus nerve electrical stimulation, reducing IL‐6 release by splenic macrophages and increasing the survival rate of septic mice from 20% to 80% [110]. Ion‐based nanogenerators, leveraging K^+^/Na^+^ transmembrane gradients, generate localized electric fields (∼50 mV/mm), which polarize macrophages toward an anti‐inflammatory M2 phenotype and increase IL‐10 secretion threefold [265]. This self‐sustaining power capability enhances the portability and practicality of these devices, enabling long‐term monitoring of the neuro‐immune system.

Nanogenerators can also regulate the activities of nerve and immune cells through electrical stimulation, a key mechanism for exploring interactions at the neuro‐immune interface. By adjusting the electric field intensity, it is possible to influence the excitability of nerve cells and the reactivity of immune cells, providing insights into their changes during disease states [266]. Studies have shown that an electric field intensity of 50 mV/mm generated by nanogenerators significantly enhances neural stem cell differentiation and increases axonal length. At an electric field intensity of 150 mV/mm, synaptic transmission efficiency in hippocampal neurons improves, leading to a marked enhancement in long‐term memory formation [267]. Moreover, high‐frequency electric fields (100 Hz) suppress the release of pro‐inflammatory cytokines like TNF‐α, while low‐frequency fields (10 Hz) promote the secretion of anti‐inflammatory factors such as IL‐10 [268]. This modulation of electric field intensity offers new possibilities for the treatment of neuroimmune diseases.

The integration of sensor functions into nanogenerators enables real‐time monitoring of the neuro‐immune interface. For example, nanogenerators can track cellular electrophysiological properties or biomarker dynamics, providing critical insights into inflammatory responses, cellular viability, neural signaling, and other essential biological processes. Such real‐time data acquisition contributes to early diagnosis and treatment, advancing the development of personalized medicine [187]. Studies have demonstrated the integration of triboelectric nanogenerators (TENGs) with biomarker‐specific sensors for real‐time monitoring of dynamic inflammatory responses. TENG‐based systems with IL‐6 sensitivity, for instance, exhibit rapid responsiveness during acute inflammatory phases, offering real‐time data to guide precision therapeutic interventions [269].

Nanogenerators can also incorporate adaptive functions, enabling them to adjust their operating states in response to environmental changes such as temperature and pH [270]. Studies have shown that poly(N‐isopropylacrylamide) (PNIPAM)‐based nanogenerators exhibit temperature‐sensitive properties. At 37°C (physiological temperature), PNIPAM undergoes a phase transition that alters the nanogenerator's output characteristics. This modulation enables precise regulation of neural stem cell differentiation and immune cell activity [271]. Furthermore, graphene oxide (GO)‐integrated nanogenerators can monitor extracellular pH variations in real time and adaptively adjust electric field intensity based on pH changes. Under low‐pH conditions, such as during acute inflammation, these nanogenerators autonomously enhance electric field output, promoting the secretion of anti‐inflammatory factors like IL‐10 [272]. This adaptive capacity allows nanogenerators to function more effectively in dynamic biological environments, thereby improving therapeutic efficacy.

The multi‐functional integration of nanogenerators enables simultaneous energy harvesting, signal transmission, sensing, and adaptive regulation. This integrated design enhances system efficiency and allows for comprehensive monitoring and regulation of the neuro‐immune interface, driving forward the potential of nanogenerators in biomedical technologies.

## 4 Application in Neuroimmunomodulation

Nanogenerators have demonstrated promising potential in the field of neuroimmunomodulation by enabling precise, self‐powered stimulation of neural and immune systems. Their ability to harvest biomechanical energy from physiological activities, such as muscle contraction or cerebrospinal fluid flow, allows them to generate localized electrical signals without the need for external power sources. These electrical cues can modulate neuroimmune interactions, influencing processes such as neuroinflammation, nerve regeneration, and immune cell activation. For instance, implantable nanogenerators composed of biocompatible and degradable materials can be strategically positioned at injury sites to provide continuous, adaptive stimulation, promoting nerve repair while minimizing inflammatory responses [273]. Moreover, the integration of nanogenerators with flexible biosensors enables real‐time monitoring of local microenvironmental changes—such as cytokine levels and electrical activity—facilitating personalized and dynamic modulation strategies. Through these advances, nanogenerators offer a transformative platform for non‐invasive or minimally invasive therapies aimed at restoring neural‐immune homeostasis and treating neurodegenerative or autoimmune disorders.

### 4.1 Bridging Nanogenerators with Neuroimmune Modulation

Bridging nanogenerators with neuroimmune modulation represents a pioneering frontier at the intersection of bioengineering and immunotherapy. By harnessing the mechanical‐to‐electrical energy conversion capabilities of nanogenerators, researchers are developing innovative strategies to modulate neuroimmune interactions with unprecedented precision. These self‐powered systems can generate localized electrical stimuli in response to physiological activities, offering a minimally invasive means to regulate neural circuits and immune responses. This emerging approach holds transformative potential for treating a range of neurological and inflammatory disorders, opening new avenues for non‐pharmacological, adaptive, and highly targeted therapies.

#### 4.1.1 Mechanistic Basis: Electrical and Mechanical Signals in Neuroimmunity

Neuroimmunity is a complex process that involves the intricate interaction between the nervous and immune systems. These two systems, traditionally viewed as separate, are deeply interconnected and communicate through various signaling mechanisms. Electrical and mechanical signals play a crucial role in modulating neuroimmune responses, influencing both neural and immune cell activities. Understanding the mechanistic basis of these signals is essential for unraveling the complexities of neuroimmune disorders and developing novel therapeutic strategies. By examining the role of electrical and mechanical signals, researchers can uncover new insights into how these signals contribute to neuroinflammation, immune regulation, and neural plasticity.

**FIGURE 27:**
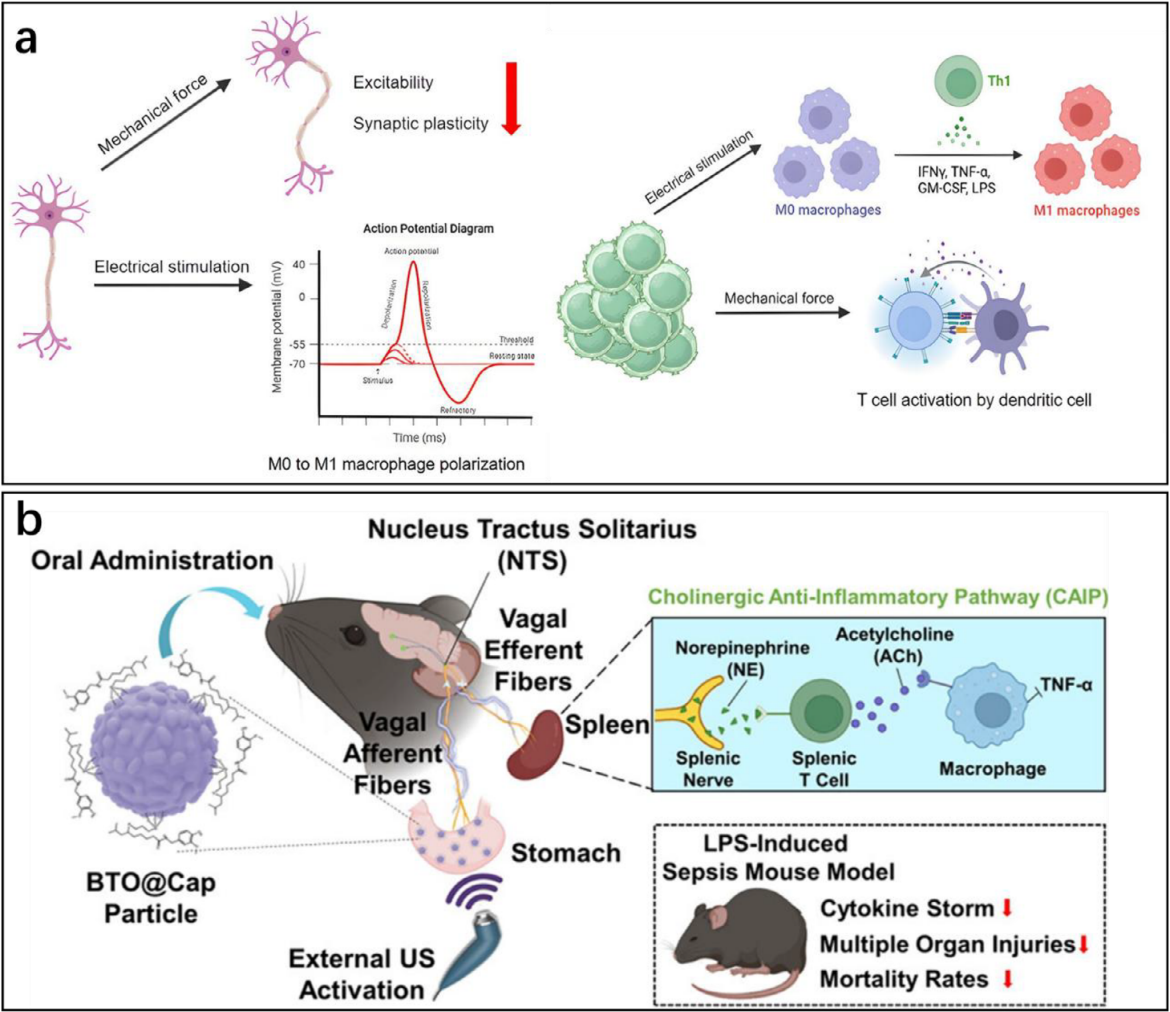
(a) The behaviors of neural cells and immune cells under the influence of electrical stimulation and mechanical force, respectively. (b) Noninvasive vagus nerve electrical neuroimmunomodulation. Reproduced with permission [276]. Copyright 2025, American Chemical Society.

Electrical signaling is fundamental to the functioning of both the nervous and immune systems. Neurons communicate through electrical impulses, or action potentials, which transmit information across synapses (see Figure 27a). Similarly, immune cells, such as macrophages and T‐cells, respond to electrical stimuli in their environment. The interplay between electrical signals in neurons and immune cells forms a critical axis of neuroimmune communication, influencing the onset and progression of neuroinflammatory diseases [274].

Mechanical signals, including shear stress, tension, and deformation, are also integral to neuroimmune interactions. These signals arise from physical forces such as blood flow, tissue movement, or changes in cell shape. Mechanical forces exert a profound influence on both neurons and immune cells. For example, mechanical deformation of neurons can affect their excitability and synaptic plasticity, while immune cells are highly sensitive to mechanical stimuli, which can alter their migratory behavior and activation states (see Figure 27a). In the context of neuroinflammation, mechanical signals can influence the recruitment of immune cells to sites of injury or infection, enhancing or suppressing immune responses as needed [275].

At the neuroimmune interface, electrical and mechanical signals often act in concert, leading to electromechanical coupling that governs cellular responses. For instance, neurons can generate electrical signals that induce mechanical changes in surrounding tissues, which, in turn, affect immune cell migration and activation. Conversely, immune cells can generate mechanical forces that impact neuronal activity, creating a feedback loop of signaling. Precise regulation of the neuroimmune system can be achieved through non‐invasive vagus nerve electrical stimulation (see Figure 27b) [276]. This bidirectional communication is particularly important in maintaining homeostasis in the brain and spinal cord, where precise regulation of immune responses is crucial to prevent neurodegenerative diseases or chronic inflammation.

Ion channels are pivotal in translating electrical signals into cellular responses. In neurons, ion channels regulate the flow of ions across membranes, generating action potentials that propagate electrical signals along axons and dendrites. In immune cells, ion channels, such as voltage‐gated and ligand‐gated channels, mediate the movement of ions in response to electrical stimuli, contributing to processes like cell migration, activation, and cytokine secretion [277]. For example, ion channels such as the transient receptor potential (TRP) channels are involved in the sensing of mechanical stimuli in both neurons and immune cells. Understanding how these ion channels contribute to neuroimmune signaling can provide insights into the molecular mechanisms underlying neuroinflammatory diseases and potential therapeutic targets [278]. For instance, electrical stimulation‐induced membrane potential changes can block the opening of potassium channels, reducing K^+^ efflux and reversing the membrane polarization state of activated microglia. This process activates JAK kinases, which phosphorylate STAT proteins. The phosphorylated STAT proteins then dimerize and translocate into the nucleus, thereby inhibiting the initiation of pro‐inflammatory signaling pathways. By downregulating potassium channel activity, electrical stimulation directly reduces the transcription and release of pro‐inflammatory factors such as IL‐6. Additionally, electrical stimulation activates voltage‐gated calcium channels, leading to a moderate increase in intracellular Ca^2^^+^ concentration. This subsequently activates calmodulin‐dependent phosphatases, which inhibit the nuclear translocation of pro‐inflammatory pathways such as NF‐κB, further suppressing IL‐6 gene transcription [279].

**FIGURE 28:**
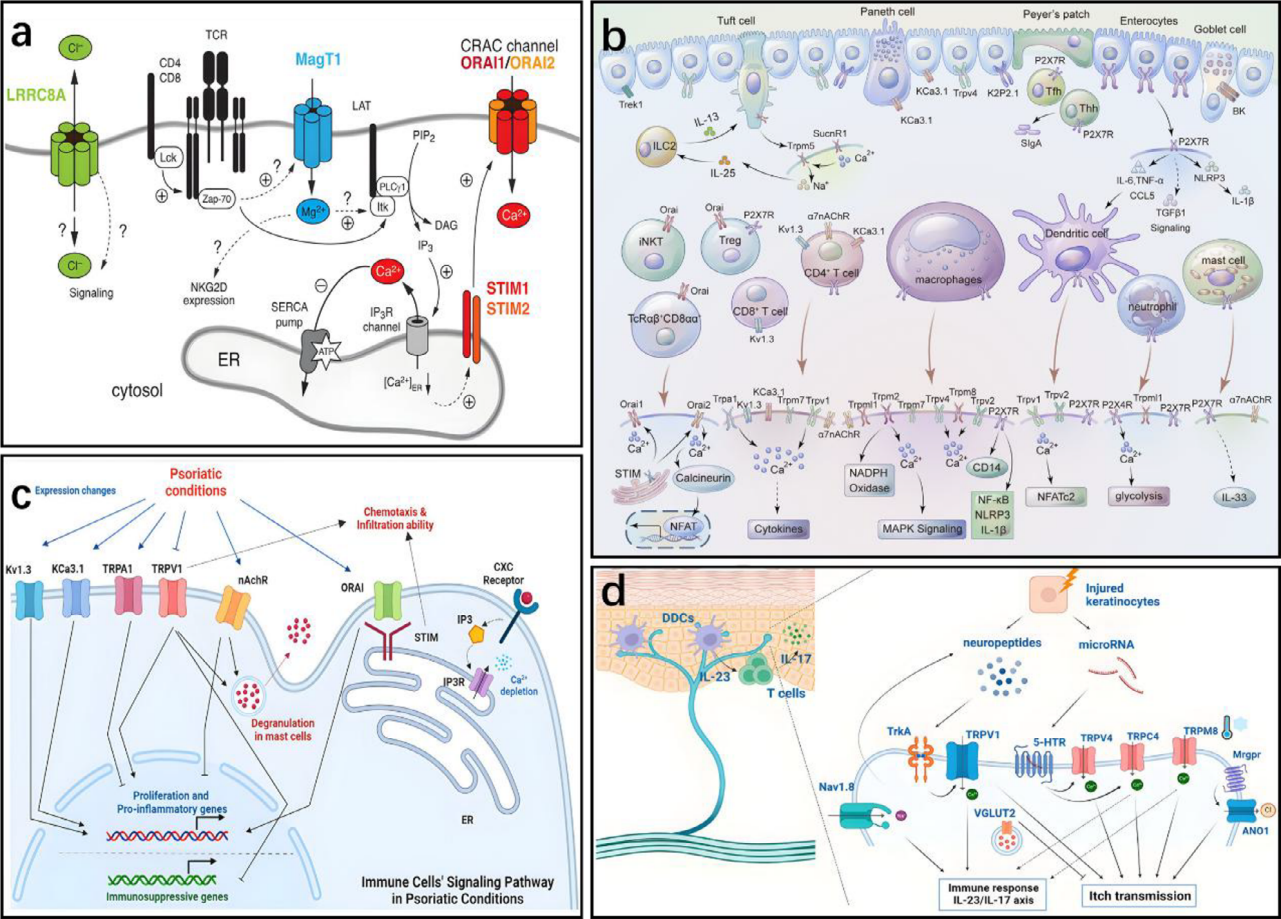
(a) Mutations interfering with the function or expression of ion channels (ORAI1, STIM1, LRRC8A) or transporters (MAGT1) in T cells cause channelopathies and immunodeficiency. Reproduced with permission [280]. Copyright 2018, Elsevier. (b) Summary of cell types and associated ion channels involved in gut immunity based on current literature. Reproduced with permission [281]. Copyright 2022, Rockefeller University Press. (c) The signaling pathways of ion channels in immune cells under psoriatic conditions. The signaling pathways involving ion channels in immune cells under psoriatic conditions are interactive and regulate the characteristics of immune cells. (d) The signaling pathways of ion channels in sensory neurons in psoriasis. The signaling pathways involving ion channels in sensory neurons contribute significantly to the sensory symptoms, such as itch and pain. Reproduced with permission [282]. Copyright 2024, MDPI.

As shown in Figure 28a, alterations or expression mutations in relevant ion channels in T cells can lead to ion channel diseases and immunodeficiency. Mutations in the ORAI1 and STIM1 genes encoding the Ca^2^^+^ release‐activated Ca^2^^+^ (CRAC) channel in immune cells, the Mg^2^^+^ transporter MAGT1, and the Cl^−^ channel LRRC8A all result in immunodeficiency and increase susceptibility to infections [280]. Additionally, various ion channels have been detected in multiple cell types throughout the gastrointestinal tract, playing a key role in synchronizing signals among different intestinal cell components that coordinate GI immune responses (see Figure 28b) [281]. Studies on signaling pathways of ion channels in immune cells and neurons under psoriatic conditions show that acetylcholine receptors, TRP channels, Ca^2^^+^ release‐activated channels, chloride channels, and potassium channels each exert specific functions to maintain skin homeostasis [282]. Dysregulation of ion channels plays a critical role in the pathophysiology of psoriasis, affecting various aspects of epidermal cell function, immune responses, and sensory neuronal signaling (see Figure 28c,d).

Mechanotransduction is the process by which immune cells convert mechanical signals into biochemical responses. This process is crucial for immune cell activation, migration, and differentiation in response to changes in the extracellular matrix (ECM) or mechanical forces from surrounding tissues. In neuroimmune interactions, mechanotransduction pathways allow immune cells to sense and respond to mechanical cues in the brain and spinal cord, which can influence neuroinflammation. For instance, macrophages and microglia, the resident immune cells of the central nervous system, respond to mechanical signals from damaged neurons or disrupted tissue architecture. These responses play a central role in modulating the inflammatory environment, either promoting tissue repair or contributing to chronic neuroinflammation [283].

The mechanistic understanding of electrical and mechanical signaling in neuroimmune interactions holds significant therapeutic potential. Targeting these signals can offer novel treatment strategies for a range of neuroimmune diseases, such as multiple sclerosis, Alzheimer's disease, and Parkinson's disease. For example, modulating electrical signaling through the use of electrical stimulation devices or ion channel blockers could help regulate immune responses in the brain. Additionally, therapeutic approaches that manipulate mechanical signals, such as using scaffolds or biomaterials to influence immune cell behavior, could enhance tissue regeneration and repair in neuroinflammatory conditions. By harnessing the power of electrical and mechanical signals, it may be possible to develop more effective and personalized therapies for neuroimmune diseases.

#### 4.1.2 Advances in Nanogenerator‐Driven Neuroimmune Applications

Neuroimmunology is an interdisciplinary field that investigates the interactions between the nervous and immune systems, exploring how these two systems influence and regulate each other in both health and disease [284]. There exist intricate mutual regulatory mechanisms between the nervous and immune systems. Neurons interact with immune cells such as T cells, B cells, and macrophages through the release of neurotransmitters like norepinephrine and acetylcholine, which can modulate immune cell activity and function. Conversely, immune cells secrete cytokines such as tumor necrosis factor‐α (TNF‐α) and various interleukins, which can impact neuronal survival, development, and function, thereby influencing nerve conduction and neural plasticity.

During infection or injury, the activation of the immune system can lead to neuroinflammation. For example, microglia—the resident immune cells of the central nervous system (CNS)—become activated and release inflammatory mediators that affect neuronal function. However, the nervous system can also suppress excessive immune responses, thereby protecting tissues from further damage. In neurodegenerative diseases, dysregulation of neuroimmune interactions can exacerbate neuroinflammation and accelerate neuronal injury. In the brains of Alzheimer's disease patients, microglial, and astrocyte activation is commonly observed, leading to the release of inflammatory factors and persistent chronic inflammation [285]. Elevated levels of cytokines such as IL‐1β and TNF‐α are closely associated with β‐amyloid deposition and neuronal death. The disruption of immune regulation may lead to auto‐aggressive responses against neurons, thereby accelerating disease progression.

Several studies have explored the use of monoclonal antibodies targeting β‐amyloid to reduce its cerebral deposition. By regulating microglial and astrocyte activity to inhibit excessive inflammatory responses, this strategy has demonstrated potential for neuroprotection. In Parkinson's disease, neuroinflammation is similarly recognized as a crucial component of the disease pathology. Inflammatory factors released by activated microglia contribute to the damage of dopaminergic neurons [286]. Moreover, abnormal activation of T cells and B cells is implicated in the pathogenesis of Parkinson's disease, potentially causing direct neuronal damage through inflammatory pathways. Alpha‐synuclein, a key component of Lewy bodies, is a neuropathological hallmark of Parkinson's disease. Its aggregation is closely linked to neurotoxic cascades that ultimately result in neurodegeneration [287]. Current research focuses on therapies aimed at targeting the spread, production, aggregation, and degradation of alpha‐synuclein, including strategies such as blocking its cellular receptors or enhancing lysosomal/autophagic clearance to mitigate disease symptoms.

In autoimmune diseases, the immune system erroneously attacks the CNS, resulting in neurological dysfunction. Multiple sclerosis (MS) exemplifies such conditions, where autoreactive T cells and B cells penetrate the blood‐brain barrier, leading to demyelination and neuronal injury. In MS, localized inflammatory responses not only inflict direct nerve damage but also impair regenerative capacities, leading to lesion formation [101]. Similarly, in systemic lupus erythematosus (SLE), autoantibodies target the body's own tissues, including the CNS, giving rise to neuropsychiatric symptoms [288]. In the case of myasthenia gravis, effective treatments include nano‐drug delivery systems carrying anticholinesterase agents and immunosuppressants.

Additionally, therapeutic approaches such as intravenous immunoglobulin administration or plasma exchange can rapidly alleviate symptoms. Thymectomy, particularly in younger patients, can also improve symptoms by reducing damage to postsynaptic receptors at the neuromuscular junction. Treatment options for myasthenia gravis are diverse, encompassing immunosuppressive therapy, acute interventions, surgical approaches, and emerging immunotherapies [289]. Personalized treatment plans and regular monitoring are essential for optimizing patient outcomes and adjusting therapies based on disease progression.

Moreover, psychological states like stress and anxiety can influence immune function via the neuroendocrine system. In turn, immune responses can affect psychological well‐being. Studies have found elevated levels of inflammatory factors in patients with depression, suggesting that inflammation may alter mood and cognitive function by disrupting the synthesis and metabolism of neurotransmitters such as serotonin and norepinephrine [290]. Anti‐inflammatory therapies, including non‐steroidal anti‐inflammatory drugs, cytokine modulators, omega‐3 polyunsaturated fatty acids, minocycline, statins, and probiotics, are emerging strategies for treating depression [291]. Inflammatory responses may also exacerbate anxiety symptoms and impair the brain's stress‐response systems. The gut microbiota plays a key role by interacting with the nervous system via immune pathways, further influencing emotional and anxiety‐related behaviors, highlighting the relevance of neuroimmunology in understanding and treating anxiety disorders [292].

Neuroimmunology has revealed the intricate mechanisms underpinning the interaction between the nervous and immune systems. A deeper understanding of these relationships not only advances our knowledge of physiological and pathological processes but also opens new avenues for the development of innovative therapeutic strategies targeting neuroimmune‐related diseases.

#### 4.1.3 Key Innovations in Design Targeting Neuroimmune Interactions

The key innovations of nanogenerators are reflected across multiple dimensions, including materials, structural design, working principles, energy harvesting efficiency, application expansion, and system integration. These advancements not only enhance the performance of nanogenerators but also significantly broaden their application potential across various fields, offering new strategies for sustainable energy solutions.

Currently, nanogenerators employ materials with outstanding electrical conductivity and mechanical properties, such as perovskite structures and two‐dimensional (2D) materials, to improve energy conversion efficiency. Biocompatible materials like chitosan and polylactic acid (PLA) have also been introduced. For instance, a nanogenerator fabricated from a composite of PLA and polycaprolactone (PCL) can gradually degrade in vivo, aligning its degradation cycle (4–6 weeks) with the tissue repair timeline. This system has been applied to spinal cord injury repair, demonstrating high biosafety and suitability for implantable medical devices [293]. Furthermore, reports have highlighted the use of self‐healing materials: a polyvinyl alcohol‐borax hydrogel‐based nanogenerator can autonomously heal within 5 min of fracture through dynamic chemical bonds (such as hydrogen bonds and disulfide bonds), restoring over 90% of its output performance [294].

In terms of structural design, flexible substrates (e.g., polydimethylsiloxane, PDMS), multilayer heterostructures, biomimetic structures, self‐supporting structures, and nanowire/nanosheet assemblies are widely adopted. These strategies enhance contact area and friction, thereby improving energy conversion efficiency and enabling seamless integration with wearable devices. For example, a triboelectric nanogenerator (TENG) modified with TiO_2_ nanowire arrays achieved a charge density of 250 µC/m^2^—five times greater than that of a planar structure—owing to its nanoscale rough surface [295]. Similarly, a porous PDMS friction layer mimicking the microstructure of lotus leaves optimizes both hydrophobicity and contact area, resulting in an output power density of 3.5 W/m^2^ [296]. Oriented BaTiO_3_/PVDF piezoelectric fiber bundles, designed to mimic the contraction‐relaxation behavior of muscles, exhibit an impressive strain sensitivity of 0.8 V/%. Self‐supporting structures, formed via material self‐assembly or cross‐linking without external substrates, further reduce interfacial energy loss. For instance, a self‐supporting carbon nanotube sponge‐based TENG, with a porosity as high as 90%, maintains 98% output stability under an 80% compression ratio, making it ideal for energy harvesting in extreme environments [297].

Nanogenerators also exhibit significant innovation in energy harvesting mechanisms. By leveraging diverse effects—piezoelectric, triboelectric, thermoelectric, and photovoltaic—they align closely with the goals of sustainable development [298]. Modern nanogenerators are capable of not only harvesting mechanical energy but also integrating with sensors to monitor environmental parameters such as temperature, humidity, and pressure in real‐time [299]. This multifunctional integration enables simultaneous energy harvesting and environmental sensing, making them ideal for applications in smart cities and the Internet of Things (IoT).

An important feature of nanogenerators is their ability to function as independent, self‐powered systems. They can harness environmental mechanical vibrations and human body movements to operate autonomously, eliminating reliance on external power supplies. This attribute greatly extends the operational lifetime of wearable devices and wireless sensor networks.

Moreover, remarkable improvements in energy conversion efficiency have been achieved by combining multiple effects—such as the thermoelectric, triboelectric, and piezoelectric effects—to allow efficient energy harvesting under diverse mechanical stimuli [300]. The incorporation of novel nanomaterials and optimized microstructures has further enhanced energy output and charge generation capabilities under mechanical strain.

For biomedical applications, designs emphasize biocompatibility, allowing nanogenerators to convert physiological movements into electrical energy within the body [301]. Their adaptive capabilities enable real‐time adjustment of operating states according to environmental conditions, thereby optimizing energy harvesting performance. Meanwhile, the development of nanogenerators capable of operating under extreme temperature and humidity conditions has significantly expanded their application versatility.

Finally, the intelligent and networked evolution of nanogenerators has introduced a new dimension to their functional innovation. Intelligent energy management systems can dynamically allocate harvested energy, thereby optimizing energy utilization efficiency and further broadening the potential of nanogenerators in next‐generation technologies.

#### 4.1.4 Advantages of Nanogenerators (NG) Compared with Traditional Neuromodulation

Conventional neuromodulation technologies, represented by deep brain stimulation (DBS), vagus nerve stimulation (VNS), and transcranial magnetic stimulation (TMS), have been applied in the treatment of conditions such as Parkinson's disease and epilepsy, yet face limitations in terms of energy supply, adaptability to the body, and clinical accessibility. In contrast, nanogenerators (NGs), leveraging their self‑powering capabilities and micro‑/nanoscale structural advantages, demonstrate significant potential for clinical application (Table 8).

**TABLE 8:** Comparison between nanogenerators and conventional neuromodulation.

	Energy supply mode	Device form factor	Clinical application status	Refs.
Nanogenerator (NG)	Self‐powered, harvesting biomechanical energy (heartbeat, respiration, movement) for conversion to electricity, with no need for external batteries or power sources.	Miniaturized, flexible design, small in size (millimeter‐scale), highly conformable to tissue, with good biocompatibility.	Currently in the research stage, safety and efficacy have been validated in animal models, not yet in large‐scale clinical trials.	[302]
Deep brain stimulation (DBS)	Relies on an external battery, with a lifespan of 3–7 years, requiring periodic surgical replacement.	Implantable device, pulse generator implanted subcutaneously in the chest, relatively bulky (thickness ∼9‐15 mm), electrodes implanted deep in the brain.	Widely used clinically, FDA‐approved for Parkinson's disease, essential tremor, dystonia, and treatment‐resistant depression.	[303]
Vagus nerve stimulation (VNS)	Battery‐powered pulse generator, battery lifespan of 3–5 years, requiring surgical replacement.	Implantable device, pulse generator implanted subcutaneously below the left clavicle, helical electrode coiled around the vagus nerve, relatively fixed size.	FDA‐approved for drug‐resistant epilepsy and treatment‐resistant depression, with over 200,000 clinical cases treated.	[304]
Transcranial magnetic stimulation (TMS)	Traditional devices require thousands of watts, powered by an external source	Traditional equipment is large, weighing tens of kilograms	FDA‐approved for depression, migraine, obsessive‐compulsive disorder, etc., with traditional devices widely used in clinical settings.	[305]

### 4.2 Mechanistic Insights into Nanogenerator‐Mediated Neuroimmune Modulation

Nanogenerator‐mediated neuroimmune modulation operates through the generation of localized electrical and mechanical stimuli that interact with both neural and immune components. The electrical outputs produced by nanogenerators can influence neuronal excitability, synaptic transmission, and the release of neurotransmitters, thereby modulating neuroimmune communication pathways. Simultaneously, these electrical signals can regulate the activity of immune cells such as microglia, macrophages, and T cells by altering cytokine secretion profiles and inflammatory responses. Mechanistically, the application of nanogenerator‐derived stimuli has been shown to activate ion channels, modulate membrane potentials, and influence intracellular signaling cascades, including calcium influx and NF‐κB pathway activation. This dual modulation of neural and immune functions facilitates a controlled microenvironment that promotes tissue repair, suppresses chronic inflammation, and enhances neuroprotection [13, 306]. Furthermore, the mechanical cues generated by certain nanogenerators mimic endogenous biomechanical forces, providing additional regulatory input to mechanosensitive neural and immune receptors. Collectively, these mechanisms highlight the potential of nanogenerators as powerful tools for precise and dynamic neuroimmune interventions.

#### 4.2.1 Nervous Regulation of Immunity

The nervous system regulates immune cell functions through the release of various neurotransmitters. For example, norepinephrine can bind to specific receptors on immune cells, influencing their proliferation, differentiation, and cytokine secretion. Under conditions of stress, sympathetic nerve activation leads to the release of norepinephrine, which can suppress the activity of immune cells such as macrophages, reducing their phagocytic capacity and cytokine production, thereby partially inhibiting the immune response [307]. Additionally, the hypothalamic–pituitary–adrenal (HPA) axis plays a crucial role in the neural regulation of immunity. Upon stimulation, the hypothalamus secretes corticotropin‐releasing hormone (CRH), which triggers the pituitary gland to release adrenocorticotropic hormone (ACTH). ACTH then acts on the adrenal cortex, promoting the secretion of glucocorticoids. Glucocorticoids exert broad immunosuppressive effects by inhibiting the activity of various immune cells and reducing the production of inflammatory mediators and cytokines [308, 309]. Specifically, they can suppress T lymphocyte proliferation and cytokine secretion, as well as diminish the antibody‐producing capacity of B lymphocytes, helping to prevent excessive immune responses [310].

Dopaminergic neurons release dopamine, which acts on lymphoid cells such as ILC2. Dopamine binds to the DRD1 receptor on the surface of ILC2. The absence of the Drd1 gene weakens the inhibitory effect of dopamine, enhancing the ILC2 response. After binding, dopamine impairs the mitochondrial oxidative phosphorylation (OXPHOS) pathway in ILC2, affecting energy metabolism, inhibiting the proliferation of ILC2 and the secretion of type‐2 cytokines, and reducing its activity to alleviate allergic inflammation. Pyrithione can counteract this inhibitory effect. In the physiological state, the abundance of dopamine is negatively correlated with the number of circulating ILC2 and positively correlated with lung function. Ablation of dopaminergic neurons exacerbates inflammation, while dopamine administration alleviates it, indicating that dopamine is an inhibitory regulator of the ILC2 response in allergic airway inflammation, maintaining tissue homeostasis and regulating inflammation [311, 312].

**FIGURE 29:**
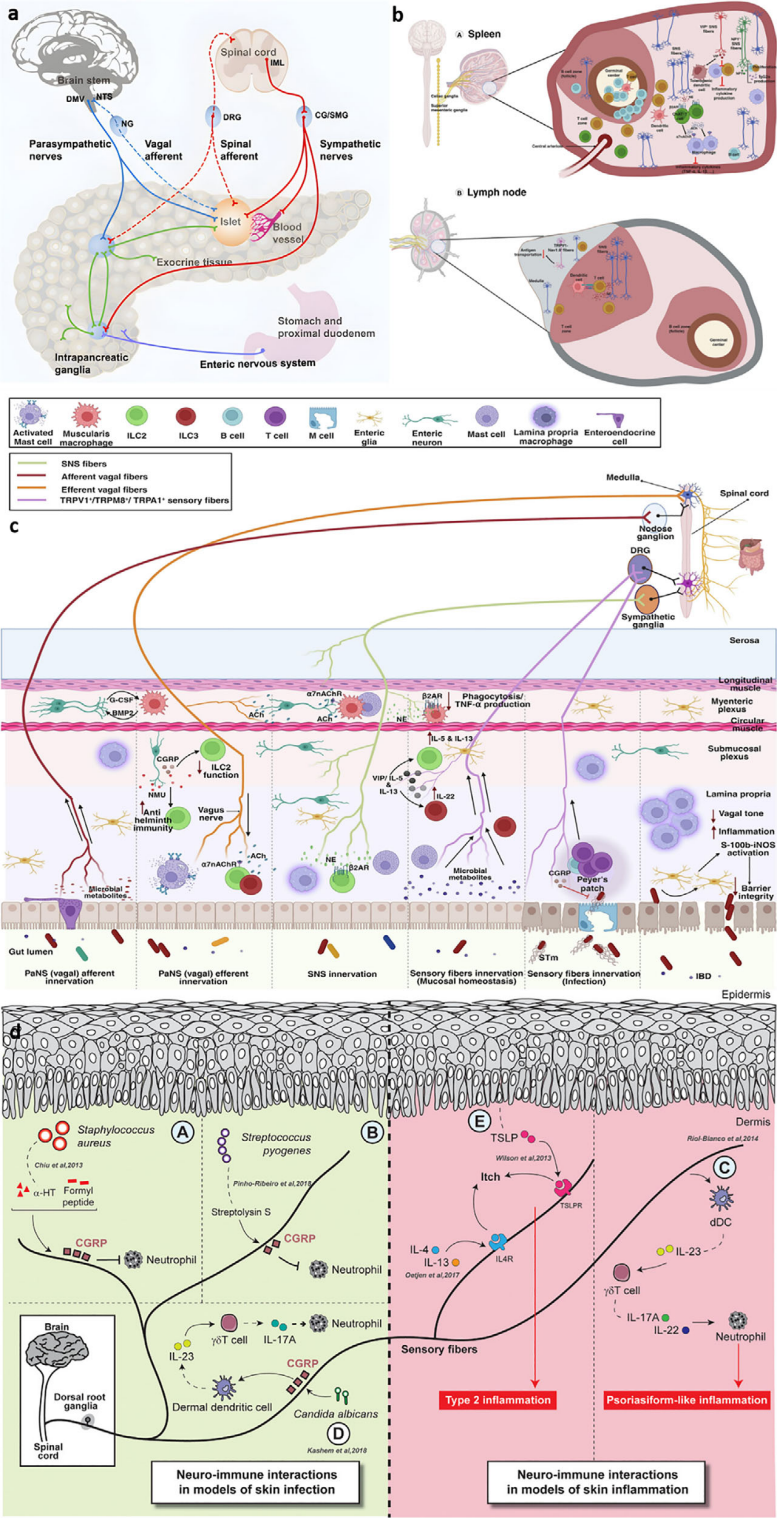
(a) Neuronal innervation of the pancreas. Reproduced with permission [315]. Copyright 2024, Elsevier. (b) Neuro‐immune interactions in the spleen and lymph nodes. (c) Neuro‐immune interactions in the GI tract (normal conditions, inflammatory bowel diseases (IBD), and infection). Reproduced with permission [314]. Copyright 2025, Elsevier. (d) Neuro‐immune interactions in models of skin infection and disease. Sensory neurons coming from DRGs modulate immune response during infection and enhance inflammation in skin diseases. Reproduced with permission [316]. Copyright 2019, Wiley.

Beyond the indirect regulation via neurotransmitters and hormones, certain nerve fibers directly innervate immune organs such as the spleen and lymph nodes (see Figure 29c). Neurotransmitters released by these fibers can act locally on immune cells, modulating their distribution, migration, and functional activities within these immune organs. The study has found that there is a direct neural pathway from corticotropin‐releasing hormone (CRH) neurons in the amygdala and paraventricular nucleus of the mouse brain to the spleen. This demonstrates the existence of a brain‐spleen neural axis that enhances adaptive immunity and reveals the dual immunomodulatory functions of CRH neurons [313].

The pancreas is under bidirectional neuroimmune regulation, where innervation and the immune environment are both critical for supporting pancreatic homeostasis (see Figure 29a). One is the classically known pituitary‐adrenal neuroendocrine immunosuppressive effect, and the other is the newly discovered immunopotentiating effect that acts directly on the spleen via neural circuits. Sensory fibers, intrinsic enteric neurons, and extrinsic autonomic nerves, including sympathetic (SNS) and parasympathetic (PaNS) fibers, innervate the intestine (see Figure 29b). Some activated enteric neurons can reduce ILC2 function by releasing calcitonin gene‐related peptide (CGRP) or enhance the anti‐helminth immunity of these cells by releasing neuromedin U (NMU). In addition to vagal efferent fibers, the gastrointestinal tract is also innervated by vagal afferent fibers, which closely connect with enteroendocrine cells and form neuropods to regulate the functions of these cells [314].

Neuronal networks connect the pancreas to the central nervous system (CNS) and enteric nervous system (ENS) to maintain metabolic activities. Studies have confirmed that various pathological conditions, including pancreatitis, diabetes, and pancreatic tumors, are caused by abnormal immune responses. Neuro‐immune interactions in skin infection and disease models reveal that sensory neurons derived from dorsal root ganglia (DRGs) modulate immune responses during infections and exacerbate inflammation in skin diseases [316]. Released bacterial products induce sensory neurons to release calcitonin gene‐related peptide (CGRP), which inhibits neutrophil recruitment to the skin and hinders pathogen clearance. In psoriasiform dermatitis models, sensory neurons activate dermal dendritic cells (dDCs) to produce IL‐23, thereby activating γδT cells to secrete IL‐17A and IL‐22 and inducing psoriasiform pathological features (see Figure 29d).

The nanogenerator based on cellulose nanofiber hydrogel generates a polydopamine coating on the surface of the anisotropic cellulose hydrogel (ACH) through in‐situ polymerization. Under ultrasonic stimulation, it efficiently produces radiofrequency signals, promotes the differentiation of neural stem cells (NSCs) into functional neurons, enhances synaptic plasticity and neural integration, facilitates the polarization of microglia toward the anti‐inflammatory M2 phenotype, scavenges reactive oxygen species (ROS), alleviates oxidative stress, and significantly regulates the NOD‐like receptor and NF‐κB signaling pathways [317].

For instance, electrical stimulation generated by indium tin oxide (ITO)‐based nanogenerators promotes the phenotypic transition of macrophages from pro‐inflammatory M1 to anti‐inflammatory M2 by modulating metabolic pathways, ion channel activity, and epigenetic modifications. Specifically, electrical stimulation (1–3 V/cm, 20–50 Hz) activates the AMPK/SIRT1 signaling pathway, inhibits nuclear translocation of NF‐κB, and downregulates the expression of M1 markers such as TNF‐α and IL‐6. Concurrently, it induces intracellular Ca^2^^+^ oscillations through voltage‐gated calcium channels (VGCCs), further activating the CaMKII/NFAT pathway and upregulating M2 markers, including arginase‐1 (Arg‐1) and CD206 [318]. Additionally, electrical stimulation stabilizes the membrane potential via modulation of K^+^ channels, suppresses NLRP3 inflammasome activation, and reduces IL‐1β release. In animal studies, this stimulation significantly increased the M2/M1 macrophage ratio in skin wounds and spinal cord injury sites, thereby accelerating tissue repair and neural regeneration.

The synaptic plasticity characteristics of molybdenum disulfide (MoS_2_) floating‐gate memory are utilized to generate bio‐inspired electrical pulses that closely resemble natural neural signals. These pulses are delivered through flexible electrodes wrapped around the sympathetic nerve chain for targeted stimulation. This low‐amplitude (average 0.175 mA) bio‐inspired pulse activates the release of norepinephrine (NE) from sympathetic nerve terminals without causing the mitochondrial swelling or neuronal structural damage commonly associated with conventional electrical stimulation [319]. The released NE subsequently acts on β_2_‑adrenergic receptors on the surface of monocytes/macrophages infiltrating the injured tendon, activating the cAMP signaling pathway to inhibit the nuclear translocation of pro‑inflammatory factors such as NF‑κB.

Building on this understanding, nanogenerator‐mediated neuroimmune modulation offers an innovative approach to actively influence neuroimmune interactions. By harvesting biomechanical energy generated from physiological activities such as body movement, breathing, or even subtle organ motions, nanogenerators can produce localized electrical stimulation that mimics or enhances natural neural signals. This capability enables precise modulation of neurotransmitter release and nerve excitation, thereby providing a non‐invasive strategy to regulate immune cell behavior. Through targeted stimulation of specific neural pathways, nanogenerators have the potential to fine‐tune immune responses, offering promising therapeutic avenues for the treatment of inflammatory diseases, autoimmune disorders, and even tissue repair and regeneration.

#### 4.2.2 Effects of Immunity on Nerves

Immune signals act as a “regulatory switch” in the nerve injury microenvironment: they not only exacerbate Schwann cell pyroptosis by triggering inflammation but also activate the mitochondrial transfer mechanism of dental pulp stem cells (DPSCs), forming a regulatory cascade of “immune signal activation–stem cell function enhancement–neural cell protection”. Ultimately, this achieves the synergy between inflammation alleviation and neural regeneration, providing a novel immune regulation‐related target for stem cell‐based therapy of peripheral nerve injury [320]. After activation, the immune system produces a wide range of cytokines, such as interleukins (ILs) and tumor necrosis factors (TNFs), which can influence neural function through multiple pathways [321, 322]. On one hand, cytokines can act directly on receptors expressed by neurons, regulating cellular excitability and synaptic transmission.

**FIGURE 30:**
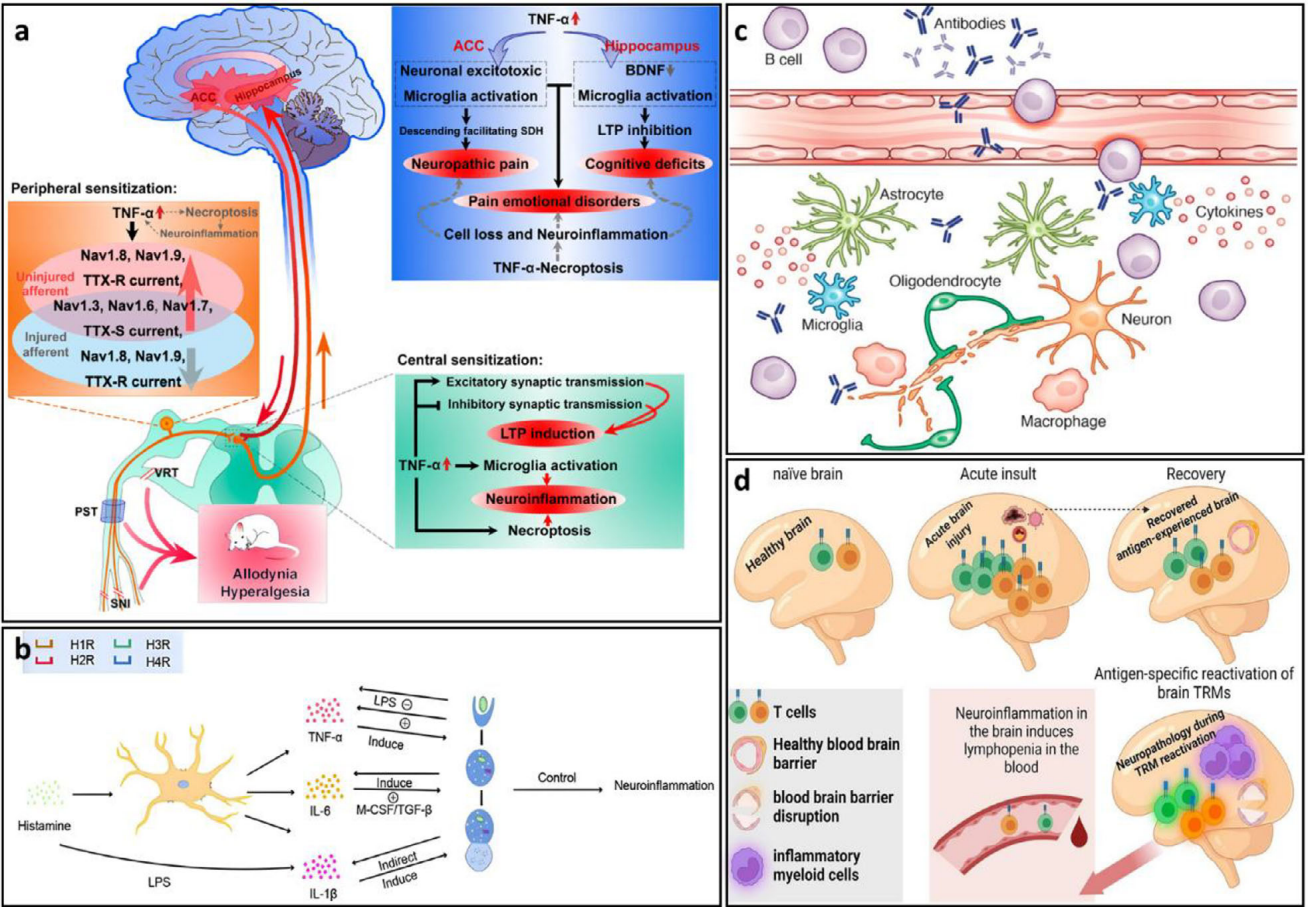
(a) Potential mechanisms underlying peripheral and central sensitization via TNF‐α or the TNF‐α–necroptosis pathway in neuropathic pain. Reproduced with permission [331]. Copyright 2022, MDPI. (b) Mechanisms by which histamine regulates neuroinflammation through different receptors. Reproduced with permission [325]. Copyright 2024, Elsevier. (c) Activated B cells can produce antibodies in an inflammatory setting. Reproduced with permission [332]. Copyright 2021, MDPI. (d) Brain resident memory T cells rapidly expand and initiate neuroinflammatory responses following CNS viral infection. Reproduced with permission [333]. Copyright 2023, Elsevier.

For example, IL‐1β has been shown to enhance neuronal excitability and alter synaptic signaling, thereby impacting nerve communication. On the other hand, cytokines can indirectly modulate neuronal function by affecting glial cells. For instance, TNF‐α can activate microglial cells, leading them to release neuroactive substances that, in turn, influence neuronal survival, plasticity, and signaling dynamics [323]. For instance, miR‐138 not only optimizes the immune microenvironment by inhibiting inflammation and exerting anti‐apoptotic effects, creating favorable conditions for the survival and differentiation of dental pulp stem cells (DPSCs), but also drives the differentiation of GABAergic neurons through regulating the GATAD2B/MTA3/WNTs axis, thereby achieving the synergistic therapy of “immune regulation – cell survival – neural regeneration”. Specifically, miR‐138 can target and degrade GATAD2B, disrupt the stability of the nucleosome remodeling and deacetylase (NuRD) complex, promote the nuclear‐to‐cytoplasmic translocation of MTA3, and activate the Wnt/β‐catenin signaling pathway. This process guides DPSCs to differentiate directionally into GABAergic neurons (expressing GAD2 and GABBR2 markers), replenishes the inhibitory neurons lost after stroke, and ultimately repairs the neural structure and function impaired by stroke [324]. As shown in Figure 30a, tumor necrosis factor‐α (TNF‐α) can drive cytokine storms and stimulate a cascade of other cytokines in pain‐related pathways, promoting the induction and modulation of neuropathic pain through peripheral (primary afferent) and central (spinal cord) sensitization.

During inflammatory responses, the immune system also releases various inflammatory mediators, such as prostaglandins and histamines. These mediators can act on peripheral nerve endings, causing sensations such as pain, and can influence the neuroendocrine system [325, 326]. For example, prostaglandins sensitize nociceptive receptors, enhancing the body's sensitivity to pain stimuli, and can disrupt the function of neuroendocrine regulatory centers like the hypothalamus, resulting in hormonal imbalances. Histamine, an autacoid and inflammatory mediator synthesized from histidine via the action of histidine decarboxylase (HDC), regulates the phenotypic conversion of microglia and astrocytes, inhibits the production of pro‐inflammatory cytokines, alleviates inflammatory responses, and modulates the pathology of neurodegenerative diseases (see Figure 30b).

Immune cells secrete a large number of pro‐inflammatory cytokines such as interleukins and tumor necrosis factors. These cytokines can alter the permeability of the blood–brain barrier, making it easier for harmful substances to enter the brain. Meanwhile, they can also activate microglia and astrocytes in the brain. After activation, microglia undergo changes in morphology and function, releasing more inflammatory mediators. This further exacerbates the inflammatory response, damages neurons and glial cells, disrupts nerve conduction and the balance of neurotransmitters, and ultimately triggers neuroinflammation [327]. Moreover, immune system disorders may lead to autoimmune reactions, where immune cells mistakenly recognize components in the nervous system as foreign antigens and launch attacks [328]. B cells or T cells can induce neurological diseases through peripheral immune mechanisms (such as the production of cytokines and antibodies) or segregated central nervous system (CNS) mechanisms (see Figure 30c,d). For example, peripherally activated T cells can cross the blood‐brain barrier to damage neural tissues by releasing pro‐inflammatory cytokines (e.g., IFN‐γ, TNF‐α) or induce autoreactive B cells to produce autoantibodies (e.g., anti‐myelin basic protein antibodies) that mediate demyelinating lesions.

Locally activated lymphocytes within the CNS may directly contribute to the formation of neuroinflammatory microenvironments, exacerbating neuronal damage. This cross‐system immune interaction plays a critical pathogenic role in neuroimmunological diseases such as multiple sclerosis, Alzheimer's disease, Parkinson's disease, and myasthenia gravis. In addition, immune system disorders may affect the proliferation, differentiation, and migration of neural stem cells, interfering with the normal development and repair of the nervous system and aggravating the damage of neuroinflammation [329].

For example, in the pathological process of Alzheimer's disease (AD), overactivated immune responses in the hippocampus are a key driver of exacerbated neural damage: IBA1+ microglia and GFAP+ astrocytes become abnormally activated, releasing pro‐inflammatory factors such as IL‐1β and TNF‐α, thereby creating a chronic neuroinflammatory microenvironment. This inflammatory microenvironment amplifies neural damage through multiple mechanisms: on the one hand, pro‐inflammatory factors can directly disrupt the stability of neuronal cell membranes, induce mitochondrial dysfunction (e.g., ROS accumulation, membrane potential imbalance), and exacerbate tau hyperphosphorylation and A_β_ plaque deposition, forming a vicious cycle of “inflammation – pathological deposition – neural damage”; on the other hand, sustained inflammation inhibits the proliferation and differentiation of endogenous neural stem cells (NSCs), hinders the transformation of immature neurons (β‐III Tubulin+) into mature neurons (NeuN+), and impairs synaptic plasticity (e.g., reducing dendritic spine density, inhibiting long‐term potentiation (LTP)), leading to the disruption of hippocampal neural network connections and atrophy of the neural cell layer.

Furthermore, although activated microglia possess a certain capacity for A_β_ phagocytosis, chronic inflammation impairs their phagocytic efficiency and instead promotes neuronal apoptosis by releasing inflammatory mediators, which further exacerbates the progressive decline in cognitive functions (e.g., spatial learning and memory storage) and ultimately drives the progression of AD from pathological damage to clinical symptoms [330].

In this context, nanogenerator‐mediated electrical stimulation offers a novel strategy for intervening in immune‐to‐neural communication. Nanogenerators enable precise modulation of neuronal excitability and neuroglial cell activation states through localized and controllable electrical signal output by harvesting mechanical energy from physiological activities (e.g., respiratory motion, vascular pulsation, muscle contraction). For example, implantable piezoelectric nanogenerators have been applied to deliver precise microcurrents, which can modulate the activation states of microglia, thereby alleviating neuroinflammatory responses. Electrical signals can activate the TREM_2_ receptor on the surface of microglia, a marker molecule of the anti‑inflammatory M2‑type microglia. Its activation promotes cellular phagocytic function via the PI3K/Akt signaling pathway, thereby accelerating the clearance of inflammatory debris and toxic proteins. Additionally, triboelectric nanogenerators have been designed to stimulate peripheral nerves in a non‐invasive manner, which can alleviate inflammatory pain by modulating the release of neuropeptides and cytokines at nerve terminals. For neurons, low‐frequency pulses (1‐5 Hz) from nanogenerators enhance the phosphorylation level of NMDA receptors, promoting normal glutamate release and uptake, thereby preventing neuronal damage caused by excitotoxicity. In contrast, high‐frequency pulses (10‐20 Hz) regulate the activity of GABAAR receptors on GABAergic neurons, strengthening the action of inhibitory neurotransmitters and alleviating pain signal transmission induced by neural hyperexcitability.

Through targeted and adaptive stimulation, nanogenerators not only offer a means to attenuate pathological neuroimmune crosstalk but also hold promise for restoring homeostasis in neuroinflammatory diseases, such as multiple sclerosis, neuropathic pain, and even neurodegenerative disorders.

### 4.3 Therapeutic Applications in Neuroimmune Disorders

**FIGURE 31:**
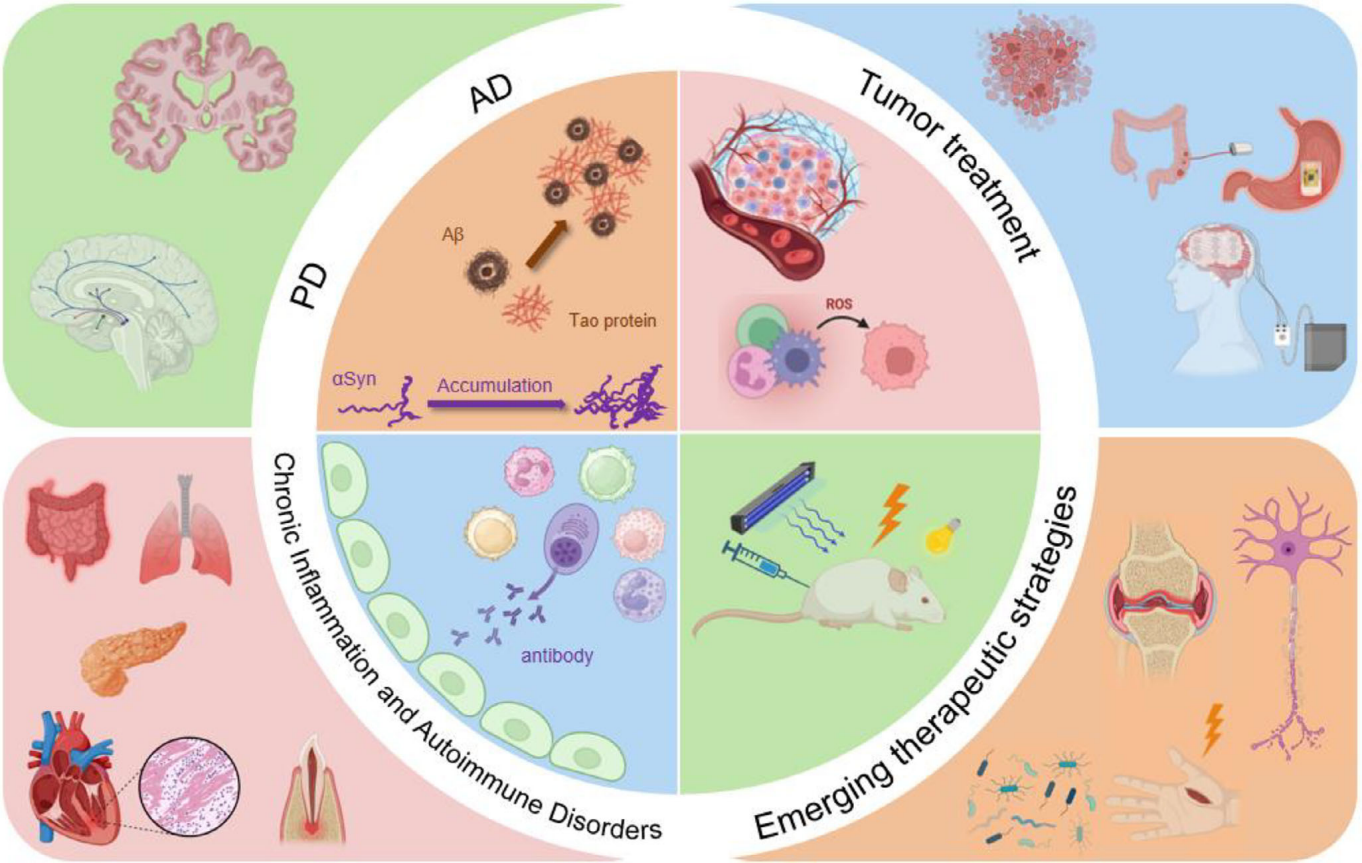
Therapeutic Applications in Neuroimmune Disorders. Created with BioRender 2026. License link: https://BioRender.com/k7tvtey.

Nanogenerators exhibit broad and multi‐dimensional therapeutic potential in the treatment of neuroimmune disorders (see Figure 31). In the context of neurodegenerative diseases, the micro‐electric fields generated via their piezoelectric and pyroelectric effects can modulate microglial polarization, suppress A_β_‐ and Tau‐induced neuroinflammation, and promote the release of neurotrophic factors, thereby helping to slow the progression of conditions such as Alzheimer's disease. For autoimmune diseases and chronic inflammatory conditions, nanogenerators can be integrated with controlled drug delivery systems to achieve targeted release of anti‐inflammatory cytokines, such as interleukin‐10 (IL‐10) and transforming growth factor‐β (TGF‐β) [334].

Furthermore, the application of localized electrical stimulation can inhibit the infiltration of hyperactivated T cells and neutrophils, contributing to the restoration of immune homeostasis. In cancer immunotherapy, nanogenerator‐generated electric fields can disrupt tumor cell membrane potentials and enhance immunogenic cell death. Simultaneously, they can promote the maturation of dendritic cells and reprogram tumor‐associated macrophages from an immunosuppressive M2 phenotype toward a pro‐inflammatory M1 phenotype, thereby synergizing with immune checkpoint inhibitors (e.g., PD‐1/PD‐L1 blockade) to amplify anti‐tumor immune responses [335]. Moreover, the intrinsic self‐powered nature of nanogenerators enables continuous modulation of the tumor microenvironment, helping to overcome immunosuppressive barriers and sustain therapeutic efficacy.

#### 4.3.1 Neurodegenerative Diseases

The central nervous system (CNS), comprising the brain within the skull and the spinal cord within the spinal canal, serves as a critical, non‐renewable component of the human nervous system. Consequently, injuries and diseases affecting the CNS—and the subsequent processes of immune repair—are of vital importance. Due to the presence of the blood–brain barrier (BBB), the CNS is considered an immune‐privileged organ, limiting the entry of peripheral immune cells and molecules. External proteins can only access the CNS through specialized carrier and receptor‐mediated transport mechanisms [336, 337].

Spinal cord injury (SCI) refers to a clinical syndrome caused by structural and functional damage to the spinal cord due to various external forces or diseases, leading to motor, sensory, and autonomic nervous dysfunction. Sustained inflammatory responses exacerbate neuronal injury and hinder nerve regeneration. Tumor necrosis factor‐α (TNF‐α) mediates immune responses in SCI, leading to neuronal apoptosis, activation of neuroglial cells, and recruitment of immune cells [338].

**FIGURE 32:**
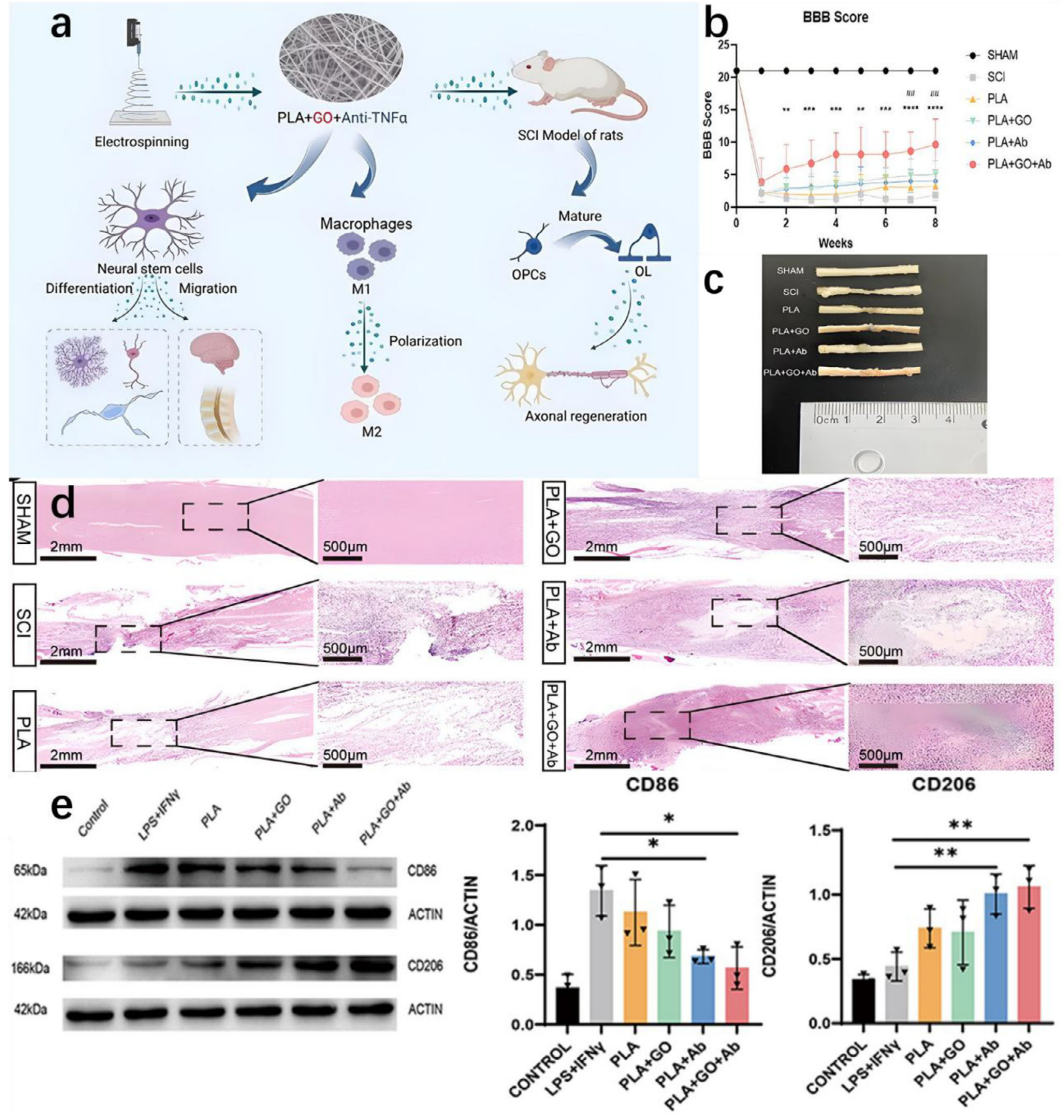
(a) An electrospun scaffold incorporating GO and anti‐TNF‐α to achieve comprehensive SCI treatment. (b) Basso–Beattie–Bresnahan (BBB) scores assessment over 8 weeks post‐injury, showing motor function recovery in different groups. The PLA+GO+Ab group demonstrated significantly improved functional recovery. (c) Macroscopic observation of the spinal cord showed that the SHAM group maintained an intact spinal cord structure, whereas the SCI group exhibited severe injury. (d) Histological analysis of H&E staining revealed severe tissue damage and cellular degeneration in the SCI group, whereas the PLA+GO+Ab group exhibited better spinal cord structure preservation and cellular repair, further demonstrating the role of the composite scaffold in promoting SCI recovery. (e) Western blot analysis of CD86 and CD206 protein expression in different groups. (n = 3). Reproduced with permission [339]. Copyright 2025, Dove Medical Press Ltd.

Its persistent overexpression exacerbates tissue damage and creates an inhibitory microenvironment for nerve repair. Therefore, researchers have designed an electrospun scaffold incorporating polylactic acid (PLA), graphene oxide (GO), and anti‐TNF‐α antibodies (Ab) (see Figure 32a). Graphene oxide (GO) can promote neural electrical stimulation and support long‐term neural differentiation and axonal growth, while anti‐TNF‐α antibodies (Ab) can inhibit early inflammatory responses, establishing a favorable immune microenvironment for nerve regeneration [339]. As shown in Figures 32b‐e, the PLA+GO+Ab group demonstrated significantly improved motor function recovery, and results from H&E staining and Western blot analysis also indicated that the PLA+GO+Ab group exhibited better preservation of spinal cord structure and cellular repair effects.

This electrospun scaffold for spinal cord injury treatment showcases unique advantages through the synergistic action of multiple components. By integrating the triple mechanisms of “structural support + electrical conduction simulation + immunomodulation”, it breaks through the functional limitations of single materials in traditional spinal cord injury repair, providing a novel strategy for clinical translation that combines biocompatibility and functional guidance. Its multi‐target synergistic effect is expected to drive the treatment of spinal cord injuries from “structural repair” to “functional reconstruction” [340].

**FIGURE 33:**
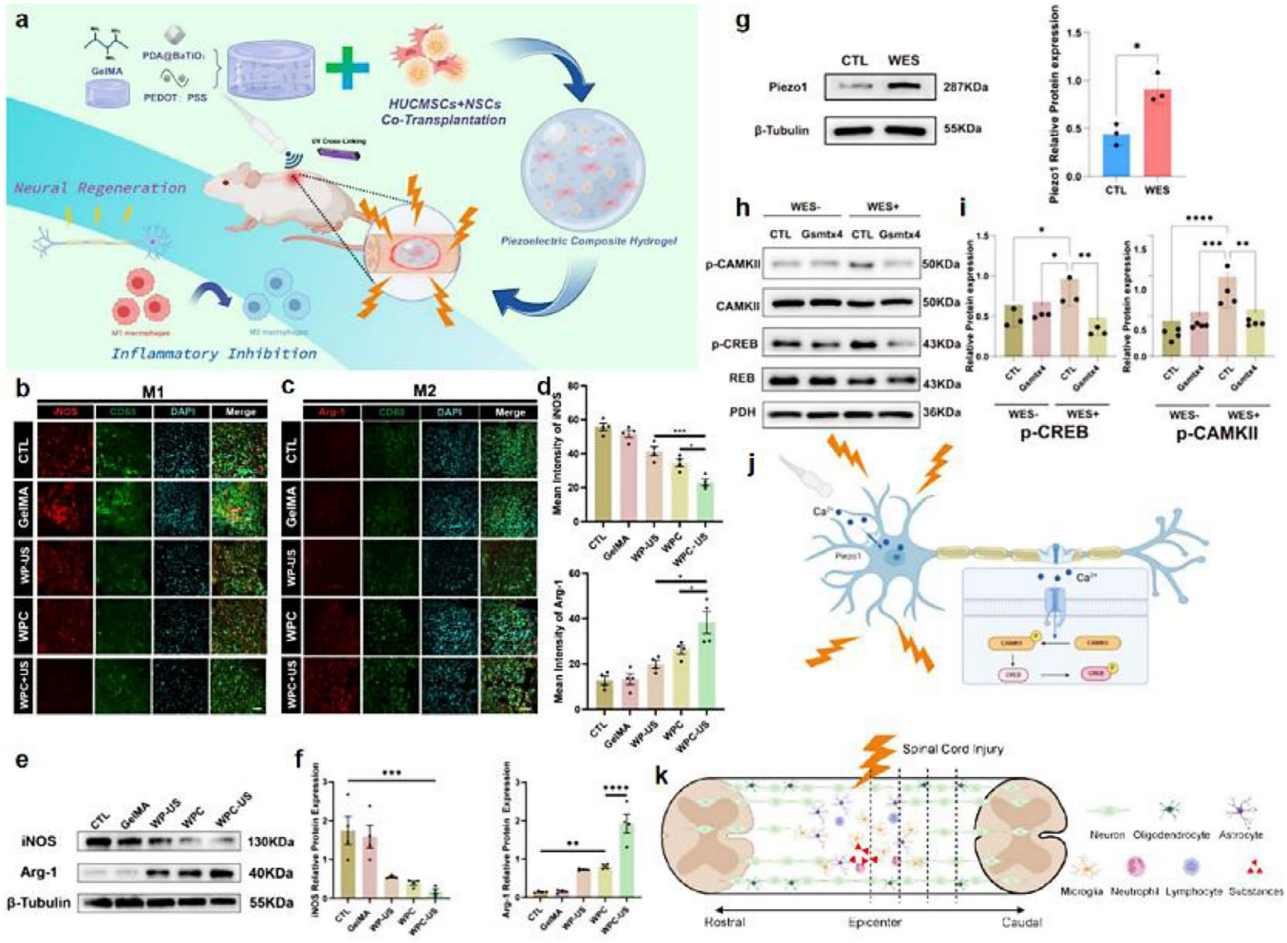
(a) Schematic of the WPC hydrogel for recovery after SCI. The WPC hydrogel is activated by an external US to generate electrical cues. (b) Immunofluorescence image of iNOS expression in macrophage/microglia. Scale bar = 100 µm. (c) Immunofluorescence images of Arg‐1 expression in macrophages/microglia. Scale bar = 100 µm. (d) Quantitative analysis of the mean fluorescence intensity of iNOS and Arg‐1 protein expression. (e, f) Representative western blot images and quantitative analysis of the expression level of iNOS and Arg‐1. (g) Expression of Piezo1 in neurons. (h, i) Representative western blot images and quantitative analysis of the expression level of p‐CAMKII/CaMKII and p‐CREB/CREB in neurons. (j) Schematic of the mechanism of WES in regulating neuronal plasticity. Created with BioRender.com. Reproduced with permission [341]. Copyright 2025, Elsevier.

Researchers have developed a piezoelectric nanogenerator composed of polydopamine (PDA)‐modified barium titanate nanoparticles (PDA@BT NPs), biocompatible conductive material PEDOT:PSS, and GelMA hydrogel (see Figure 33). Driven by ultrasound and wirelessly powered, this device promotes the transition of macrophage polarity during the subacute phase. As the injury progresses to the chronic phase, it reduces the mechanical hardness of scar tissue, reshapes and relieves cystic cavities, and improves tissue repair and motor function recovery in traumatic spinal cord injury (SCI) [341].

The PLA/GO/Anti‑TNF‑α composite nanogenerator modulates the neuroimmune microenvironment through its electroactive composite material. It neutralizes the pro‑inflammatory factor TNF‑α via sustained low‑intensity electrical stimulation (∼0.5–1.2 V, 10–50 Hz), inhibits microglial M1 polarization, and does not require additional cell therapy. This approach can reduce inflammatory cytokines by 40–60% and improve motor‑function recovery (BBB score increase of 3–4 points). The ultrasound‑driven wireless piezoelectric hydrogel generates a local electric field (∼5–20 V, <1 ms pulse width) via ultrasound‑triggered piezoelectric effects, promoting neural regeneration and synergizing with stem‑cell transplantation (NSCs and hUCMSCs), resulting in a 2‑fold increase in axonal regrowth length and an 80% enhancement in synaptic density.

Compared with conventional drugs (e.g., glucocorticoids) used for spinal cord injury (SCI) treatment, nanogenerators offer targeted regulation of the local immune microenvironment without the long‑term side effects of glucocorticoids, such as osteoporosis and immunosuppression, and additionally support neural regeneration. While epidural electrical stimulation can provide immediate motor‑function improvement, it relies on an external power supply, carries risks of electrode‑induced fibrotic encapsulation, and often suffers from poor patient compliance. In contrast, the two nanogenerator‑based approaches described above—featuring self‑powering capability, controllable material degradation, targeted local immunomodulation, wireless ultrasound‑driven operation that avoids lead‑related infections, and dynamic responsiveness to mechanical loads—hold substantial promise for clinical translation in SCI therapy.

Neuroimmune interactions, particularly the communication between neurons and resident microglia (the intrinsic macrophages of the CNS), play crucial roles in maintaining brain homeostasis, mediating inflammatory responses, and driving disease pathogenesis [342]. Disruption of this delicate balance is implicated in neurodegenerative diseases such as Alzheimer's disease (AD) and Parkinson's disease (PD). Early detection and intervention are critical for slowing disease progression. Key pathological biomarkers—β‐amyloid (A_β_) in AD and α‐synuclein in PD—are central to disease development. In AD, abnormal aggregation of β‐amyloid forms extracellular plaques that disrupt neuronal function. In PD, misfolded α‐synuclein accumulates intracellularly, leading to neuronal damage and dopaminergic neurodegeneration [5, 103].

Recent advances in biosensor technologies based on nanogenerators offer promising tools for the early diagnosis of neurodegenerative diseases. These biosensors employ specific recognition elements, such as antibodies or aptamers, that bind selectively to β‐amyloid or α‐synuclein. Upon binding to target biomarkers present in biological samples (e.g., blood or cerebrospinal fluid), these interactions alter the local microenvironment of the sensor, causing detectable changes in output electrical signals (e.g., voltage, current) [343]. The high sensitivity of nanogenerator‐based biosensors allows them to capture subtle biomarker fluctuations characteristic of early disease stages, providing a robust platform for early detection.

Parkinson's disease, characterized by the progressive loss of dopaminergic neurons in the substantia nigra, leads to motor dysfunctions such as muscular rigidity, resting tremor, and bradykinesia. Current treatment modalities—including deep brain stimulation, gene therapy, optogenetics, and chemical genetics—each present advantages and limitations. In an innovative approach, Chen et al. developed a pomegranate‐inspired piezoelectric nanogenerator capable of inducing neuronal membrane depolarization through ultrasound activation. This intervention facilitated calcium ion influx, enhanced tyrosine hydroxylase (TH) activity, promoted the clearance of α‐synuclein aggregates, and ultimately improved dopamine production in PD models [344].

**FIGURE 34:**
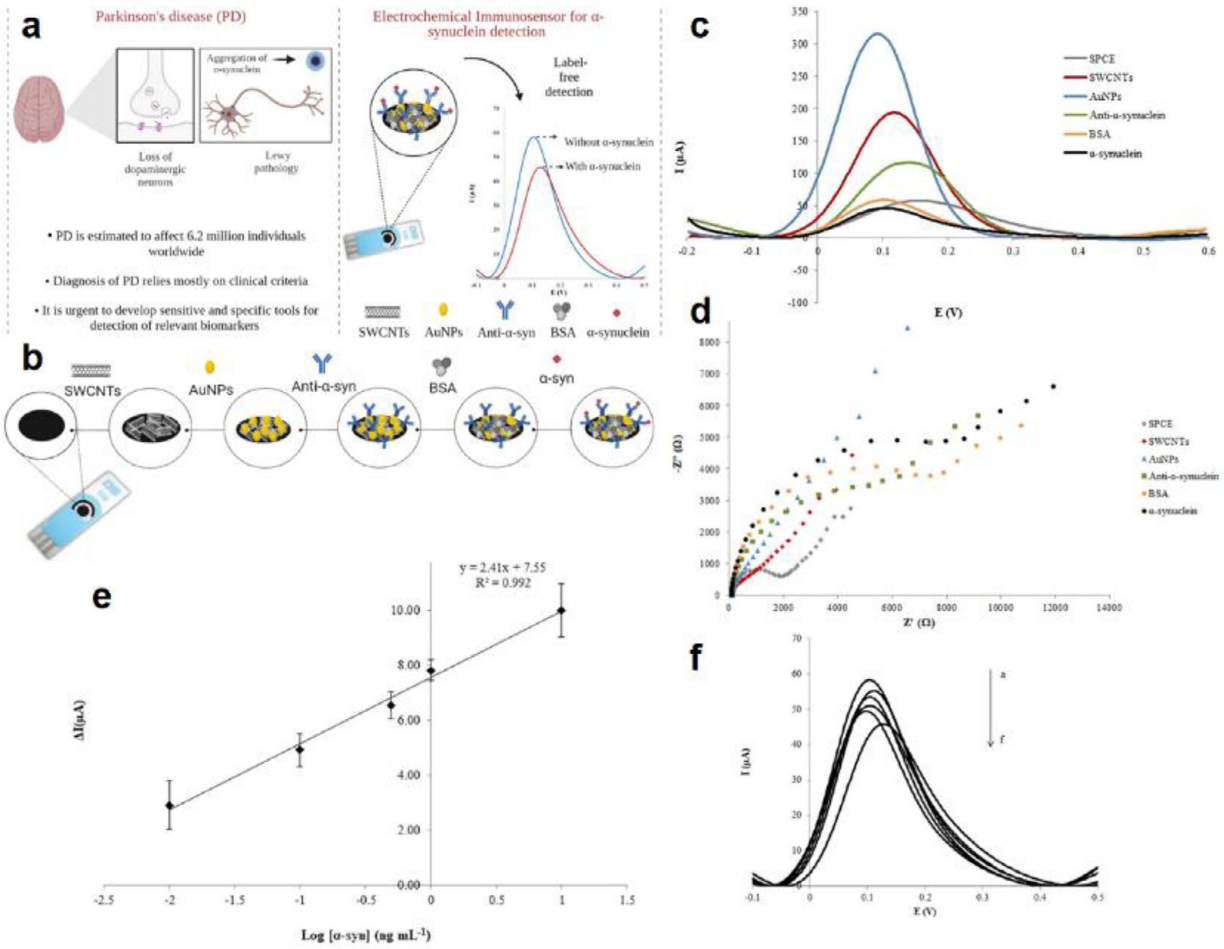
(a) A sensitive and specific electrochemical immunosensor for the detection and quantification of α‐synuclein. (b) Schematic illustration of the electrochemical immunosensor development. (c) SWV results for the optimized stages of the immunosensor development. Measurements were performed in a 2.5 mM Fe(CN)6^3^‐^/4^‐ solution in 10 mM PBS (pH 7.4). (d) EIS results for the optimized stages of the immunosensor development. Measurements were performed in a 2.5 mM Fe(CN)6^3^‐^/4−^ solution in 10 mM PBS (pH 7.4). (e) SWV response of the immunosensor to different α‐synuclein concentrations (from a to f: 0, 0.01, 0.1, 0.5, 1, and 10 ng mL^−1^) (f) Calibration curve based on the SWV signal reduction after detection of α‐synuclein concentrations (0.01, 0.1, 0.5, 1, and 10 ng mL^−1^) (n = 3). Measurements were performed in a 2.5 mM Fe(CN)6^3^‐^/4−^ solution in 10 mM PBS (pH 7.4). Reproduced with permission [345]. Copyright 2023, Elsevier.

As shown in Figure 34, researchers have developed a sensitive and specific electrochemical immunosensor for the detection and quantification of α‐synuclein based on voltammetric studies of redox indicator signals. The indicator signals decrease due to increased electron resistance upon antibody recognition of the analyte. The electrochemical immunosensor was modified via a layer‐by‐layer approach based on screen‐printed carbon electrodes (SPCEs) coated with single‐walled carbon nanotubes (SWCNTs) and gold nanoparticles (AuNPs). Specifically, the nanoscale architecture of the sensor was successfully achieved by drop‐casting an SWCNT suspension followed by electrodeposition of AuNPs at −0.2 V for 150 seconds in an HAuCl_4_ solution. Monoclonal antibodies were chemically modified and immobilized on the surface of AuNPs at an optimal concentration of 200 µg mL^−^^1^. Using the proposed immunosensor, α‐synuclein was detected in the range of 0.01–10 ng mL^−^^1^, with limits of detection and quantification of 4.1 and 12.6 pg mL^−^^1^, respectively. This method is suitable for studying and distinguishing pathological and physiological levels of α‐synuclein [345].

In Alzheimer's disease, hallmark pathologies include intracellular neurofibrillary tangles composed of hyperphosphorylated tau (p‐Tau) and extracellular amyloid plaques [346]. Non‐invasive brain stimulation techniques such as transcranial electrical stimulation have shown potential in modulating neural circuits, with anodic stimulation improving behavioral outcomes and cathodic stimulation enhancing cognitive function [347]. Improvements have been observed in memory, language fluency, emotional regulation, daily living activities, and biological rhythms, including the pupillary light reflex.

Nanogenerators have also been harnessed for direct biomarker detection. For instance, a triboelectric nanogenerator (TENG) modified with graphene quantum dots has been used to detect p‐Tau181. Specific binding of p‐Tau181 to the quantum dots induces changes in surface potential, resulting in a detectable output current shift that is linearly correlated with p‐Tau concentration, achieving an ultralow detection limit of 0.1 fM—suitable for early blood‐based screening [348]. Similarly, surfaces modified with Aβ‐specific antibodies (e.g., 6E10) enable sensitive detection of A_β_ oligomers, where binding events alter surface charge density and reduce piezoelectric output signals, allowing real‐time monitoring of A_β_ concentrations in cerebrospinal fluid (sensitivity: 1 pg/mL) [349].

**FIGURE 35:**
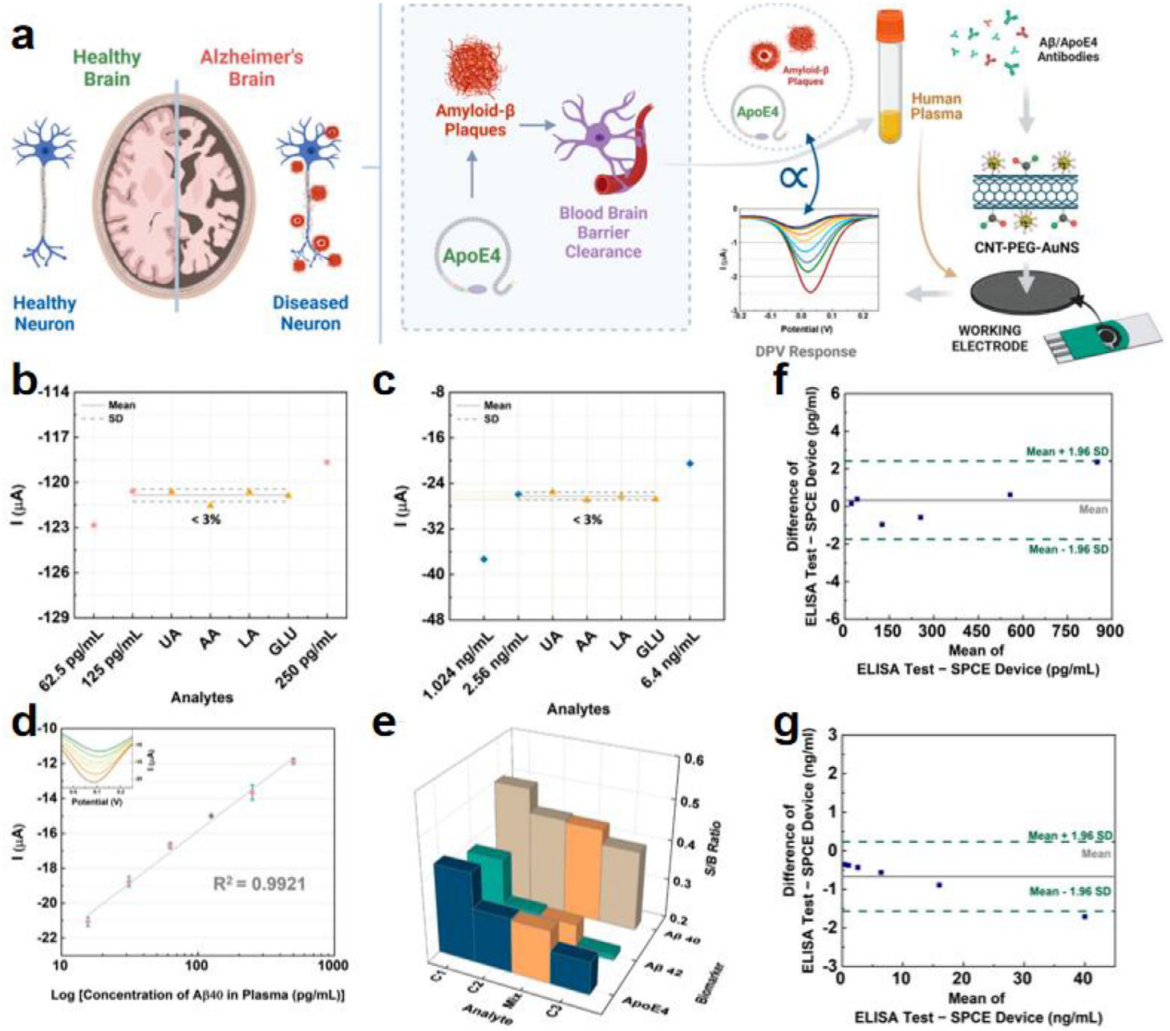
(a) Schematic representation of the proposed methodology for detecting Alzheimer's disease biomarkers. Differential pulse voltammetry (DPV) peak currents were measured for (b) amyloid‐β 42 (circle) and (c) ApoE4 (rhombus), following the addition of interferents. (d) Differential pulse voltammetry (DPV) peak currents of the CNT‐AuNS‐PEG when measuring amyloid‐β 40 as a potential Alzheimer's biomarker. (e) Evaluation of the enhanced screen‐printed carbon electrode (SPCE) for cross‐reactivity with samples containing A_β_40, A_β_42, and ApoE4. Bland–Altman plots depict the level of agreement for measurements using diluted human plasma samples containing (f) A_β_42 and (g) ApoE4. Reproduced with permission [350]. Copyright 2024, MDPI.

Amyloid plaques, apolipoprotein E4 (ApoE4), and other Alzheimer's disease (AD) biomarkers are predominantly identified in cerebrospinal fluid (CSF) due to their concentrated presence. However, the detection of these biomarkers in blood is hampered by the blood‐brain barrier (BBB), resulting in low concentrations. To address this, researchers have developed an innovative approach for electrochemical analysis of amyloid plaques and ApoE4 using screen‐printed carbon electrodes (SPCEs) enhanced with a chitosan‐coated gold nanostar immobilization matrix (see Figure 35). Gold nanoparticles exhibit excellent physicochemical, thermal, and optical properties, while the biocompatibility of chitosan facilitates precise immobilization of recognition elements and prevents interference from other substances, further enhancing the biosensor's sensitivity. Experimental studies demonstrate high consistency with the gold‐standard enzyme‐linked immunosorbent assay (ELISA), and interference tests show minimal changes in peak current. This method represents a less invasive and straightforward approach for detecting A_β_42 and ApoE4 in human plasma, enabling monitoring of Alzheimer's disease progression [350].

#### 4.3.2 Chronic Inflammation and Autoimmune Disorders

**FIGURE 36:**
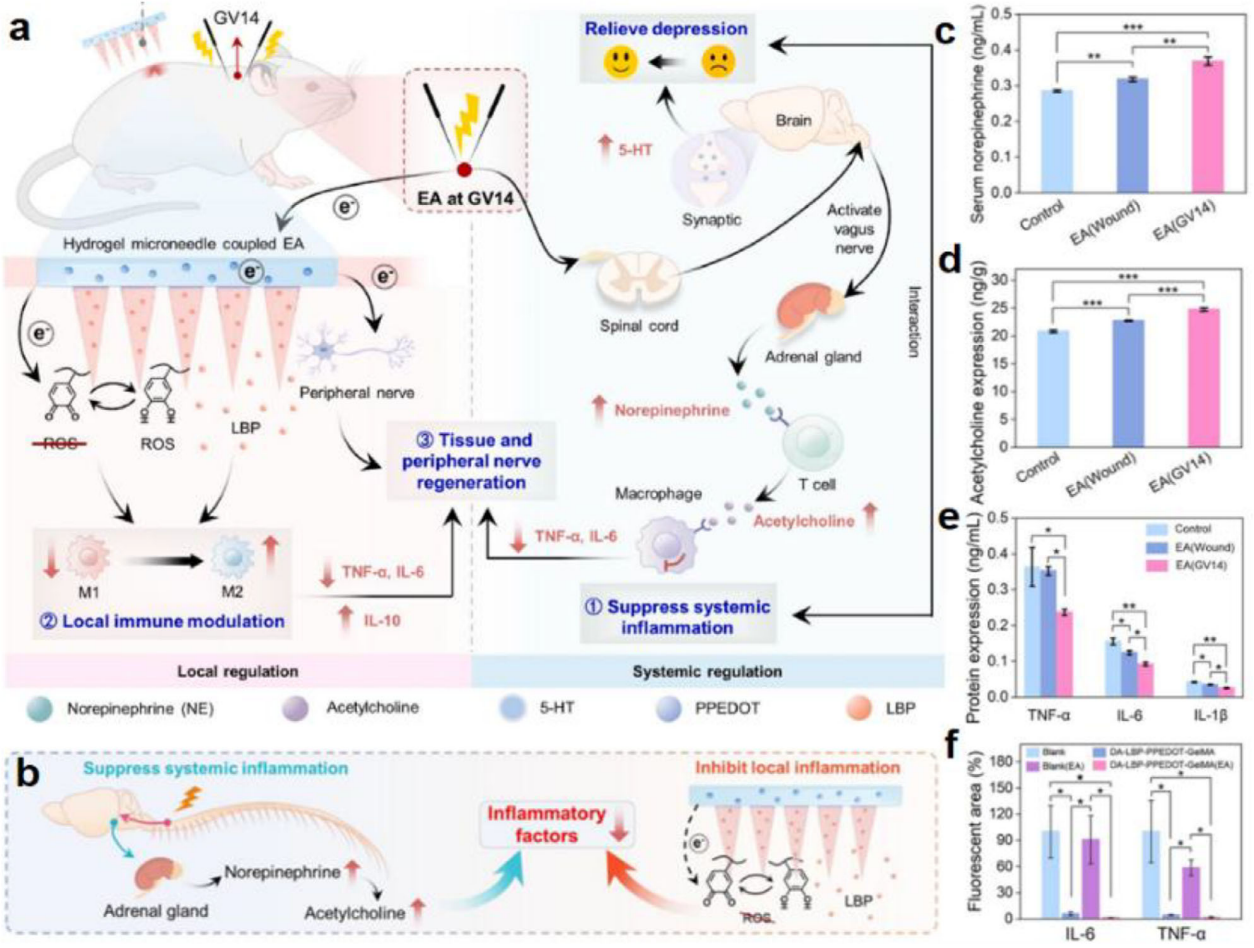
(a) Synergistic mechanism of hydrogel microneedles combined with EA at GV14 to treat depression. (b) Schematic illustration of the modulation of systemic and local inflammation. (c) Serum concentrations of norepinephrine in each group. (d) Concentrations of acetylcholine in the spleen of each group. (e) Serum concentrations of TNF‐α, IL‐6, and IL‐1β in each group. (f) Relative fluorescent area of immunofluorescence staining of IL‐6 and TNF‐α. Reproduced with permission [351]. Copyright 2025, Elsevier.

Dysregulation of the inflammatory response is linked to a wide range of diseases, including infectious, autoimmune, neurodegenerative, cardiovascular, renal, and cancerous conditions. Therefore, early detection and intervention to manage inflammation are crucial for maintaining human health. As shown in Figure 36, researchers have proposed a strategy combining electroacupuncture (EA) stimulation at the Dazhui acupoint (GV14) with polyphenol‐mediated conductive hydrogel microneedles to inhibit inflammatory responses through interactions between the inflamed site and the brain. The conductive microneedles are composed of gelatin methacrylate, dopamine (DA), DA‐modified poly(3,4‐ethylenedioxythiophene), and Lycium barbarum polysaccharides. EA at GV14 activates the vagus nerve‐adrenal axis to suppress systemic inflammation. Electroacupuncture therapy enhances central nervous system plasticity and modulates the release of acetylcholine, norepinephrine, and monoamine neurotransmitters, thereby exerting anti‐inflammatory effects [351]. This integrated approach combines the anti‐inflammatory effects of traditional acupuncture with the conductive properties of nanocomposite hydrogels, providing a new paradigm for treating inflammatory diseases through neuro‐immune regulation.

For inflammation treatment, the combination of conductive polyphenol microneedles and electroacupuncture (EA) can activate the vagus–adrenal axis. Dopamine (DA) couples with electrical signals to achieve phenol‐quinone conversion, continuously scavenges reactive oxygen species (ROS), and regulates the polarization of macrophages from the M1 to M2 phenotype, thereby downregulating TNF‐α and IL‐6 and upregulating IL‐10 [351]. In contrast, the piezoelectric scaffold Ti_3_C_2_T_x_/UiO‐66 generates electricity under ultrasound stimulation, with Ti_3_C_2_T_x_ nanosheets accelerating charge transfer. It upregulates ATP‐sensitive K^+^ (KATP) channels, downregulates voltage‐gated calcium channels (VGCCs), inhibits the Ca^2^^+^‐CaMKII‐NF‐κB signaling pathway, downregulates TNF‐α and inducible nitric oxide synthase (iNOS), and upregulates arginase 1 (Arg‐1) and IL‐10, thereby suppressing the inflammatory response and stimulating nerve regeneration [352].

Compared with traditional anti‐inflammatory drugs or antibiotics, the aforementioned two types of nano‐generators act only on the local inflammatory site, avoiding side effects such as liver and kidney damage and intestinal flora disturbance caused by the systemic distribution of drugs. In addition to anti‐inflammatory effects, they simultaneously promote tissue/nerve regeneration and improve inflammation‐related complications, while drugs mostly target only a single inflammatory target [353]. In contrast, standard neural stimulation is difficult to precisely target the local inflammatory microenvironment and relies on single‐time stimulation with transient effects. However, nano‐generators can achieve long‐term anti‐inflammation through electrical response cycles without the need for frequent stimulation.

Multiple Sclerosis (MS) is a chronic autoimmune disorder affecting the central nervous system. The condition arises when the immune system erroneously attacks the myelin sheaths that protect nerve fibers in the brain, spinal cord, and optic nerves. This results in the formation of demyelinating lesions, which disrupt the normal transmission of nerve signals, causing a variety of symptoms such as limb weakness, sensory abnormalities, visual disturbances, balance issues, and cognitive impairments [354]. An innovative solution to treating MS involves implantable nanogenerators that regulate microglial activation through a continuous electric field, helping to alleviate demyelination. Additionally, Triboelectric Nanogenerators (TENG) play a vital role in diagnosing MS by measuring electrophysiological signals from nerve and muscle tissues. These nanogenerators can also serve as power sources for electrical stimulation, promoting neuron activity and muscle function, thus offering new avenues for both diagnosis and treatment.

Rheumatoid arthritis (RA) is a chronic, systemic, autoimmune disease characterized by synovial hyperplasia and the aggregation of inflammatory cells, ultimately leading to bone destruction, joint deformity, and even disability. It is often accompanied by symptoms such as morning stiffness, joint pain, swelling, and deformity, which can cause joint dysfunction and disability and severely affect the quality of life of patients. Here, the researchers have constructed an intelligent hydrogen nanogenerator based on a metal‐organic framework (MOF) loaded with polydopamine and perovskite quantum dots. The biodegradable polydopamine with excellent photothermal conversion efficiency is applied to the photothermal therapy (PTT) of rheumatoid arthritis (RA), and the perovskite quantum dots (QDs) with unique photophysical properties are used as fluorescent signals to localize the Pt‐MOF@Au@QDs/PDA nanoparticles [355]. The Pt‐MOF@Au@QDs/PDA achieves the aggregation of rheumatoid synovial cells through the “ELVIS” effect (i.e., extravasation through leaky vasculature and subsequent isolation mediated by inflammatory cells) and extremely efficient photocatalytic hydrogen production.

**FIGURE 37:**
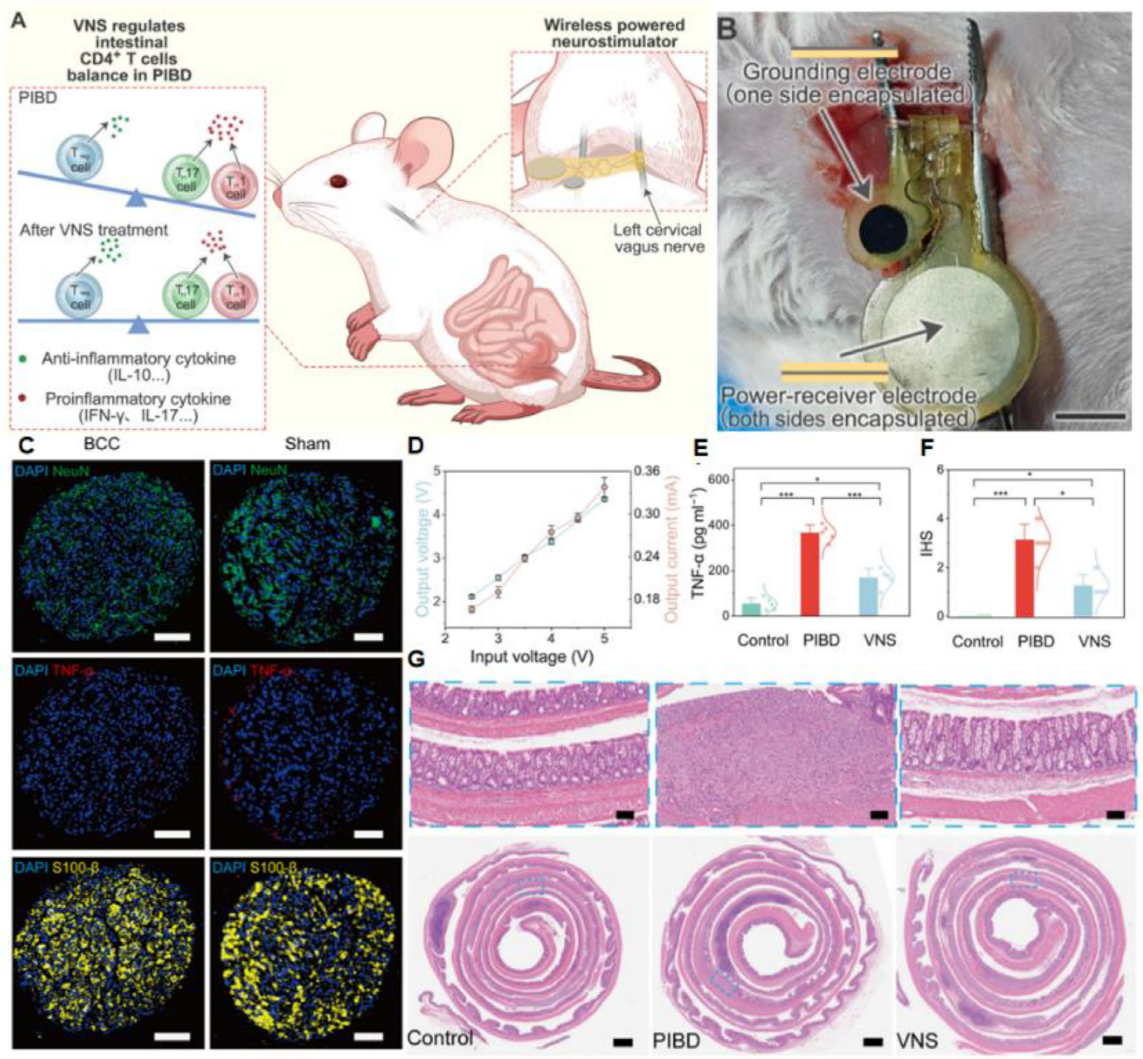
(A) Schematic of the battery‐free, wirelessly powered BCC neurostimulator for chronic vagus nerve stimulation to regulate intestinal CD4^+^ T cell balance in PIBD. (B) Surgical image showing the implantation of the BCC neurostimulator on the rat vagus nerve. Scale bar, 5 mm. (C) Representative immunofluorescence staining of the vagus nerves after 4‐week implantation. Scale bars, 50 µm. (D) Open‐circuit voltage and short‐circuit current generated by the BCC neurostimulator. (E) Intestinal concentrations of TNF‐α in the different groups (n = 5). (F) Inflammation‐related histology score (IHS) of rats’ colons in the different groups (n = 5). (G) Representative H&E staining images of rats’ colons in the different groups. Scale bars, 100 µm (top) and 1 mm (bottom). Reproduced with permission [357]. Copyright 2025, AAAS.

Inflammatory Bowel Disease (IBD), encompassing conditions such as Ulcerative Colitis (UC) and Crohn's Disease (CD), is a group of chronic inflammatory disorders of the intestine. The exact cause of IBD remains unclear, but traditional drug therapies often have limitations due to off‐target effects and systemic side effects [356]. Therefore, researchers have designed a battery‐free miniaturized neural stimulator based on biodegradable materials and capacitive coupling wireless power transmission. The biodegradable capacitive coupling (BCC) neural stimulator is composed of molybdenum (Mo) electronic components and a self‐healing biodegradable polyurethane elastomer (SBPUE) encapsulation. Programmed electrical stimulation of the vagus nerve can restore CD4+ T cell balance, enhance anti‐inflammatory effects, regulate the immune microenvironment, enhance gastrointestinal peristalsis and function, alleviate extracorporeal symptoms through gut–brain axis signal transmission, and thereby relieve IBD symptoms (see Figure 37).

#### 4.3.3 Cancer Immunotherapy and Tumor Microenvironment Modulation

**FIGURE 38:**
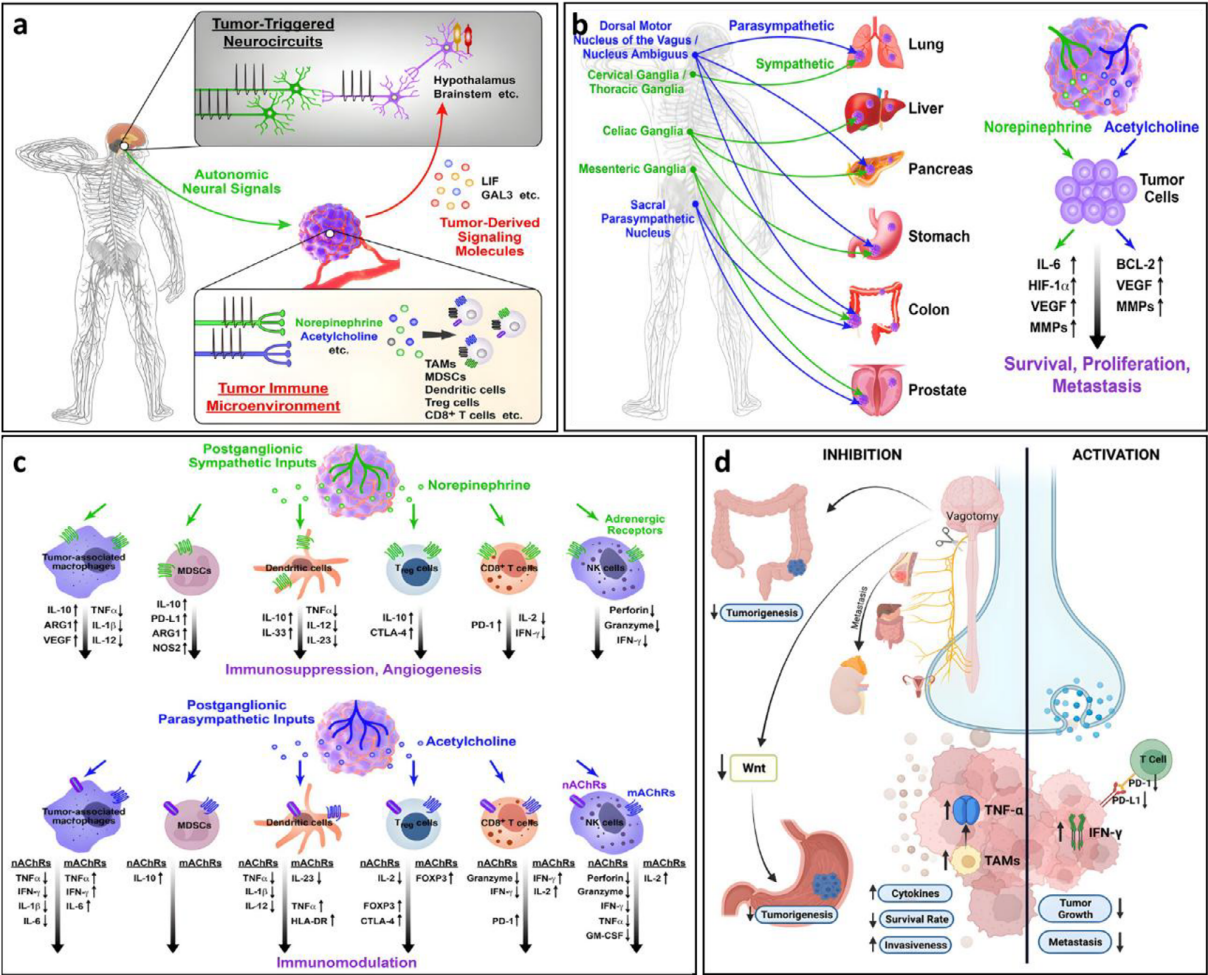
(a) Reciprocal interactions between neural signals and peripheral tumors. (b) Autonomic neural signals in common peripheral tumors. (c) Autonomic neural signals modulate the immune microenvironment of peripheral tumors. Reproduced with permission [358]. Copyright 2025, Elsevier. (d) Schematic showing how parasympathetic nervous system activation reduces tumor growth and metastasis via decreased expression of PD‐1 and PD‐L1 while increasing release of IFN‐γ from CD4^+^ and CD8^+^ tumor‐infiltrating lymphocytes. Reproduced with permission [359]. Copyright 2022, Elsevier.

Nanogenerators have emerged as a promising and versatile tool in the anti‐tumor field, with significant applications in modulating the tumor microenvironment and enhancing neuroimmune responses to improve cancer therapy. Neural signals and surrounding tumors can interact (see Figure 38a): for example, tumor cells can promote the growth of nerve fibers into tumors by releasing factors such as nerve growth factor (NGF), forming a tumor innervation network, while neurotransmitters released by nerve endings can activate receptors on tumor cell surfaces, enhancing their proliferation and invasion capabilities while inhibiting immune cell function [358].

As shown in Figure 38b, common peripheral tumors (such as lung, liver, pancreatic, gastric, colorectal, and prostate cancers) receive autonomic innervation from both sympathetic and parasympathetic nerves. Postganglionic sympathetic fibers originate from neurons in the sympathetic trunk or cervical/thoracic ganglia, while preganglionic parasympathetic fibers from the dorsal motor nucleus of the vagus nerve regulate corresponding postganglionic neurons. Autonomic nerves infiltrating the tumor microenvironment act on tumor cells via neurotransmitters like norepinephrine (sympathetic) and acetylcholine (parasympathetic), promoting tumor proliferation and metastasis through signaling pathways such as IL‐6 and HIF‐1α. As shown in Figures 38c‐d, norepinephrine released by sympathetic nerves in the tumor microenvironment acts on tumor‐associated macrophages, MDSCs, dendritic cells, Treg, CD8^+^ T cells, etc., via adrenergic receptors, promoting the expression of immunosuppressive signals (e.g., ARG1, IL‐10) and VEGF while inhibiting pro‐inflammatory factors (e.g., TNFα) to establish an immunosuppressive microenvironment [359].

Acetylcholine released by parasympathetic nerves acts on these immune cells through nAChRs or mAChRs, where nAChRs inhibit pro‐inflammatory and anti‐tumor signals while mAChRs may enhance their expression, with their complex effects influencing the outcome of anti‐tumor immunity. These nanogenerators offer multifaceted solutions, such as counteracting the hypoxic tumor microenvironment, facilitating targeted drug delivery, and enhancing the efficacy of chemotherapy through their gas‐generating capabilities.

Tumor tissues consist of regions with normal blood supply and areas of hypoxia. In hypoxic regions, tumor cells rely heavily on glycolysis to produce lactic acid. This lactic acid is transported to better‐oxygenated areas, where it participates in ATP production via the tricarboxylic acid cycle, supporting the tumor's survival and metabolic functions. Nanogenerators, such as H_2_S‐based systems, can regulate lactate production, inhibit glycolysis, and disrupt lactate transport, thus inducing acidosis. This process not only creates a hostile environment for tumor cells but also reverses the immunosuppressive tumor microenvironment, enhancing anti‐tumor immunity and triggering tumor cell death [360].

Furthermore, the presence of high levels of the antioxidant glutathione (GSH) in tumor cells contributes to their resistance against reactive oxygen species (ROS), heavy metals, and other oxidative agents [361]. By downregulating GSH levels, tumor cells are more vulnerable to oxidative damage. In this context, researchers developed a novel oxygen‐independent alkyl radical nanogenerator, copper monosulfide/2,2'‐azino‐bis(2‐imidazoline) dihydrochloride@bovine serum albumin (CuS/AIPH@BSA). This nanogenerator induces GSH depletion, thereby increasing ROS production and sensitizing tumor cells to oxidative stress. The CuS/AIPH@BSA system, triggered by near‐infrared (NIR) laser irradiation, releases alkyl radicals in hypoxic breast cancer cells, leading to effective tumor treatment without inducing drug resistance [362].

The role of ROS in cancer therapy has long been recognized, but their production is limited in tumors due to hypoxia, which results in insufficient oxygen (O_2_) and hydrogen peroxide (H_2_O_2_). To overcome this challenge, a chlorine radical nanogenerator was developed using upconversion nanoparticles (UCNPs) under NIR light to generate ‐Cl radicals. UCNPs catalyze the conversion of Ag/AgCl to produce ‐Cl independently of O_2_/H_2_O_2_ [363]. These chlorine radicals exhibit strong oxidative capabilities, making them more effective than traditional ROS therapies in targeting and destroying cancer cells.

In addition to enhancing therapeutic efficacy, nanogenerators are also instrumental in overcoming the biological barriers that impede drug delivery to solid tumors. A DDS developed by Chen et al. incorporated cisplatin and sodium nitroprusside into poly(d,l‐lactide‐co‐glycolide) (PLGA) vesicles, equipped with peroxynitrite (ONOO^−^) tumor‐specific nanogenerators [364]. The ONOO^−^ generated by these nanogenerators improves vascular permeability and facilitates drug penetration into tumors by activating matrix metalloproteinases, leading to more effective cisplatin release and apoptosis of tumor cells. Moreover, the cascade effects triggered by ONOO^−^ also enhance the chemotherapy efficacy by promoting the uptake of cisplatin by neighboring tumor cells.

Zhao et al. explored a red blood cell (RBC)‐based DDS for anti‐tumor treatment, loading doxorubicin (DOX) into RBCs. The controlled release of DOX is enhanced by electrical stimulation using a Micro‐TENG (MTENG), which perforates the RBC membrane, enabling the slow and controlled release of the drug [140]. This MTENG‐controllable DDS platform demonstrated superior effectiveness in killing cancer cells in vivo and ex vivo, even at low DOX doses, offering a promising approach for targeted cancer therapy with minimal side effects.

Gas therapies, such as hydrogen (H_2_) and carbon monoxide (CO), are also gaining attention as “green” cancer treatment strategies. H_2_ has been shown to inhibit mitochondrial function, disrupt ATP synthesis, and reduce the transport capacity of P‐glycoprotein (P‐gp) efflux pumps, thereby enhancing chemotherapy efficacy. A photoactivated H_2_ nanogenerator has shown significant potential in improving bladder cancer treatment in both in vitro and in vivo studies [365]. Similarly, CO‐assisted photothermal therapy (PTT) has been demonstrated to inhibit cancer progression by modulating cellular ATP levels and activating apoptotic pathways. CO nanogenerators, activated by NIR‐II light, enhance the sensitivity of cancer cells to chemotherapeutic drugs and PTT, amplifying the therapeutic response.

Hydrogen sulfide (H_2_S), a known inhibitor of tumor growth, has also been explored for use in nanogenerators. Xu et al. developed a mesoporous organosilicon‐based H_2_S nanogenerator (MON‐TPGS‐DOX) loaded with doxorubicin (DOX). Upon triggering by GSH, these nanogenerators release H_2_S, inducing oxidative stress and activating apoptotic signaling pathways in cancer cells [366]. This enhances tumor cell killing, demonstrating the potential of H_2_S‐based nanogenerators in enhancing the efficacy of cancer therapies.

**FIGURE 39:**
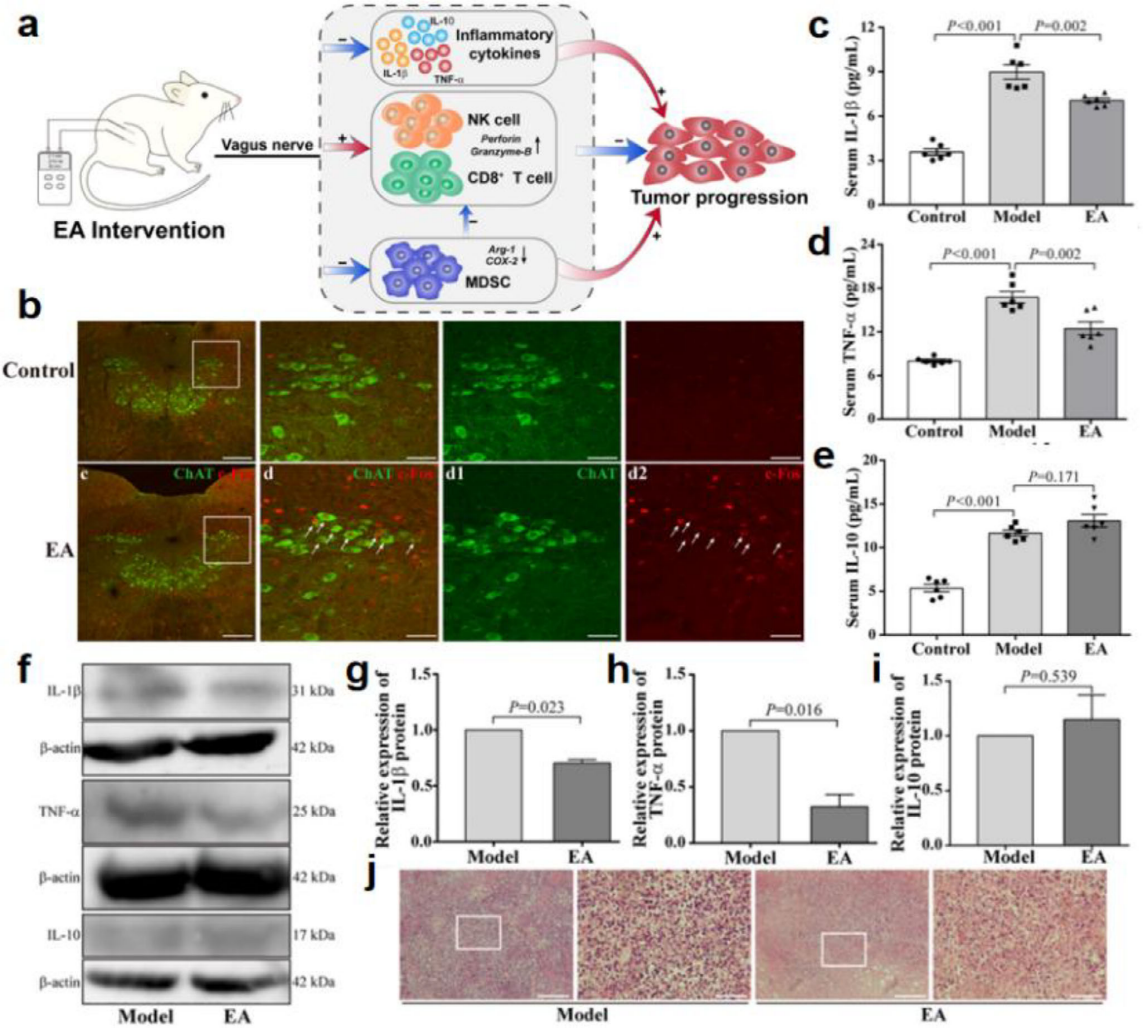
(a) Schematic diagram of electroacupuncture enhancing anti‐tumor immunity in breast tumor mice by activating the vagus nerve to regulate inflammatory cytokines. (b) Expression levels of ChAT (green) and c‐Fos (red) in the DMV neurons in the two groups. EA induced c‐Fos expression in ChAT+ DMV neurons (arrowhead). Scale bar, a, c, 200 µm, b‐b2, d‐d2, 50 µm. (c‐e) Serum levels of the cytokines IL‐1β (c), TNF‐α (d), and IL‐10 (e) in the 3 groups on the 22nd day (n = 6, one‐way ANOVA). (f–i) Representative blots (f) and expression levels of IL‐1β (g), TNF‐α (h), and IL‐10 (i) in tumor tissues between the two groups on the 22nd day (n = 5, Student's t‐test). (j) Representative HE staining of tumor tissues in the two groups. Reproduced with permission [367]. Copyright 2021, Elsevier.

Researchers developed an electroacupuncture (EA) device for insertion into the ST36 acupoint to stimulate the vagus nerve (See Figure 39a). EA intervention significantly increased the proportion and cytolytic function of CD8^+^ T cells and natural killer (NK) cells, while reducing the accumulation and immunosuppressive activity of myeloid‐derived suppressor cells (MDSCs). Figure 39b shows that electroacupuncture intervention leads to substantial c‐Fos expression in choline acetyltransferase‐positive (ChAT^+^) neurons of the dorsomedial medullary nucleus (DMV) [367]. Compared with normal mice, the levels of inflammatory cytokines in the serum of tumor‐bearing mice were significantly elevated. After EA intervention, serum levels of IL‐1β and TNF‐α significantly decreased, as did those in local tumor tissues, while anti‐inflammatory cytokines slightly increased. Additionally, HE staining of tumor tissues in the model group showed massive inflammatory cell infiltration, whereas tumor tissues in the EA group exhibited a lower degree of inflammatory infiltration. These results indicate that EA alleviated the inflammatory response in mice with breast tumors within 22 days (see Figure 39c–j).

The development of advanced nanogenerators represents a transformative approach in cancer therapy. By modulating the tumor microenvironment, enhancing targeted drug delivery, and boosting the efficacy of chemotherapy through the generation of reactive species, these nanogenerators offer new opportunities for more effective, targeted, and safer cancer treatments. Additionally, their ability to regulate neuroimmune responses presents a promising avenue for the next generation of cancer immunotherapies.

#### 4.3.4 Others

In addition to neurodegenerative diseases, chronic inflammation, autoimmune disorders, and cancer immunotherapy, emerging therapeutic strategies utilizing nanotechnology, electrical stimulation, and photothermal therapies hold significant promise in treating a broader range of neuroimmune disorders. Recent advancements have shown that these novel approaches not only address existing limitations in current treatments but also offer potential in areas previously considered challenging for conventional therapies.

**FIGURE 40:**
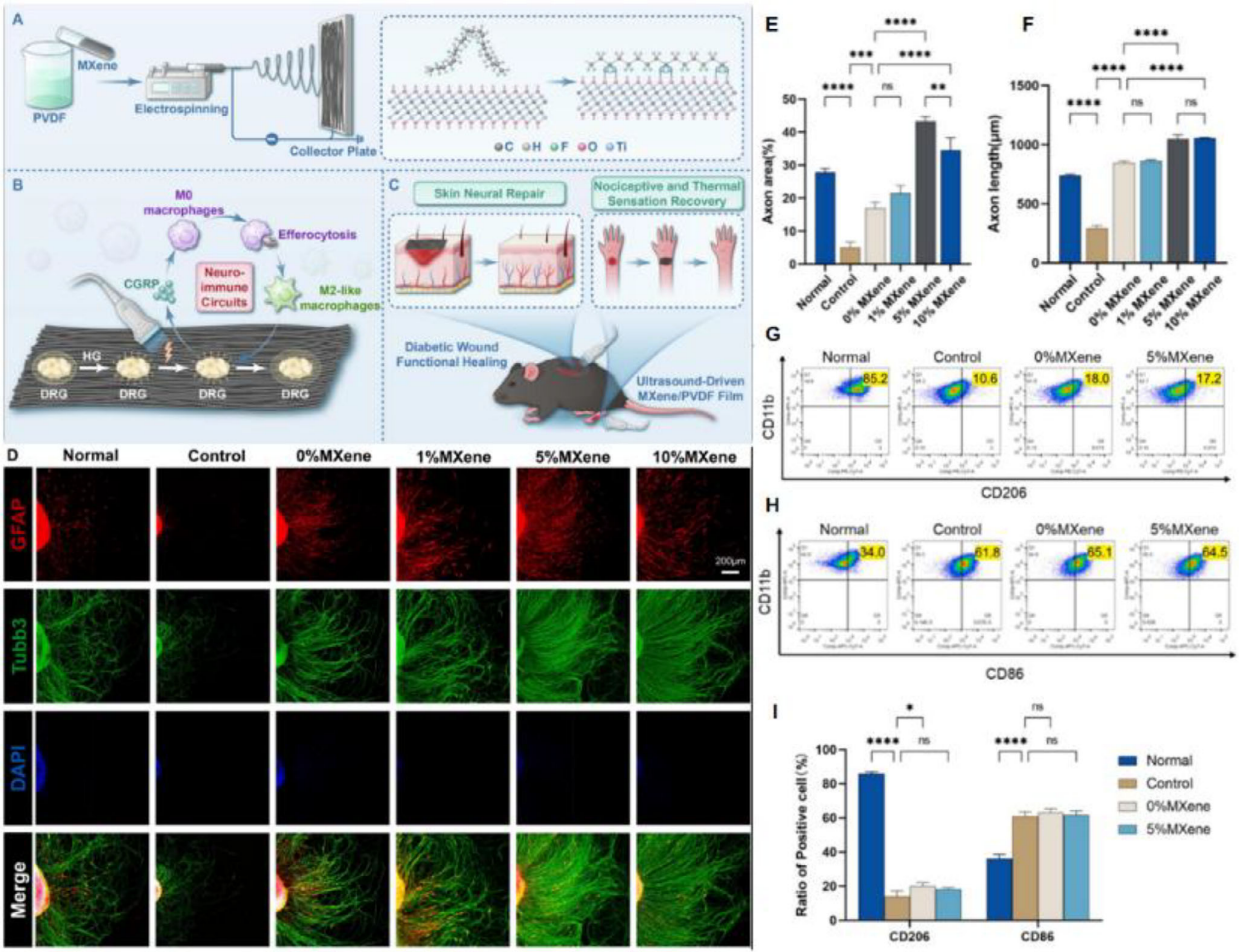
(A) Synthesis of MXene/PVDF films. (B) Mechanism of neuro‐immune regulation. (C) Functional healing of diabetic wound. (D) Immunofluorescence images showing axonal structures (green), Schwann cells(red), and nuclei (blue) in DRG explants from different treatment groups on day 3 post‐treatment. (E,F) Quantitative analysis of axonal length and area on day 3. (G,H) Representative flow cytometry plots showing CD206 and CD86 expression in BMDMs. The percentages of CD206‐positive and CD86‐positive macrophages are indicated on day 3. (I) Flow cytometry analysis of CD206 (M2 marker) and CD86 (M1 marker) expression in BMDMs. Reproduced with permission [368]. Copyright 2025, Elsevier.

Electrical stimulation (ES) therapies, particularly those integrated into wearable devices, have been explored for their ability to modulate immune responses in a variety of conditions beyond cancer and neurodegenerative diseases. These devices, including self‐powered nanogenerators, can be utilized to stimulate tissue regeneration and manage immune responses through localized electrical fields. This approach has been shown to accelerate wound healing and promote tissue repair by enhancing blood circulation, modulating fibroblast activity, and reducing inflammatory mediators [369]. Therefore, many novel multifunctional wound healing systems have been proposed. For instance, researchers have developed a low‐frequency ultrasound‐driven MXene/PVDF piezoelectric film to address the issues of neurogenesis and immune homeostasis in diabetic wound healing (see Figure 40A–C). When low‐frequency ultrasound is applied, the piezoelectric effect of the film can be converted into bioelectrical signals. This weak current can simulate neural electrophysiological signals, induce directional growth of nerve fibers, and accelerate the nerve regeneration process in diabetic wound areas. As shown in Figure 40D–I, immunofluorescence, quantitative analysis of axon length and area, and flow cytometry confirmed that the ultrasound‐driven MXene/PVDF film accelerates wound healing, supports epidermal nerve regeneration and M2 macrophage polarization, and restores sensory function in wounds. Meanwhile, the bioelectrical signals can activate ion channels and signaling pathways within immune cells, regulate the polarization state of macrophages, and promote the differentiation of anti‐inflammatory M_2_ macrophages, thereby constructing an immune microenvironment conducive to tissue repair [368]. By leveraging wearable technologies, patients can receive continuous treatment without the need for bulky equipment, offering a more accessible and cost‐effective solution.

MXene/PVDF piezoelectric films convert exogenous mechanical stimulation into electrical signals, promoting DRG axonal regeneration and Schwann cell functional activation, while increasing CGRP secretion. This neuropeptide binds to the CLR/RAMP1 receptors on the surface of macrophages, activating the cAMP‐PKA and MAPK signaling pathways, inhibiting NF‐κB‐mediated pro‐inflammatory responses, and facilitating M2 phenotype polarization. Additionally, these films promote epidermal nerve regeneration and sensory function recovery, thereby accelerating wound healing [368]. In contrast, the DAT‐pMXene@bFGF scaffold can generate electrical signals by collecting ion flow from wound exudate. By activating ion channels (e.g., Ca^2^^+^ channels) and signaling pathways (PI3K/AKT, MEK/ERK) on the cell membrane, it promotes the migration of fibroblasts (NIH‐3T3) and vascular endothelial cells (HUVECs) toward the wound center, while enhancing cell proliferation activity to accelerate granulation tissue formation and re‐epithelialization [352].

However, silver sulfadiazine cream, a conventional therapeutic agent, is prone to enzymatic degradation and requires continuous supplementation, failing to regulate neuro‐immune circuits. Standard neural stimulation demands professional operation, and high voltage may induce tissue damage, making it unable to adapt to the dynamic wound healing microenvironment.

**FIGURE 41:**
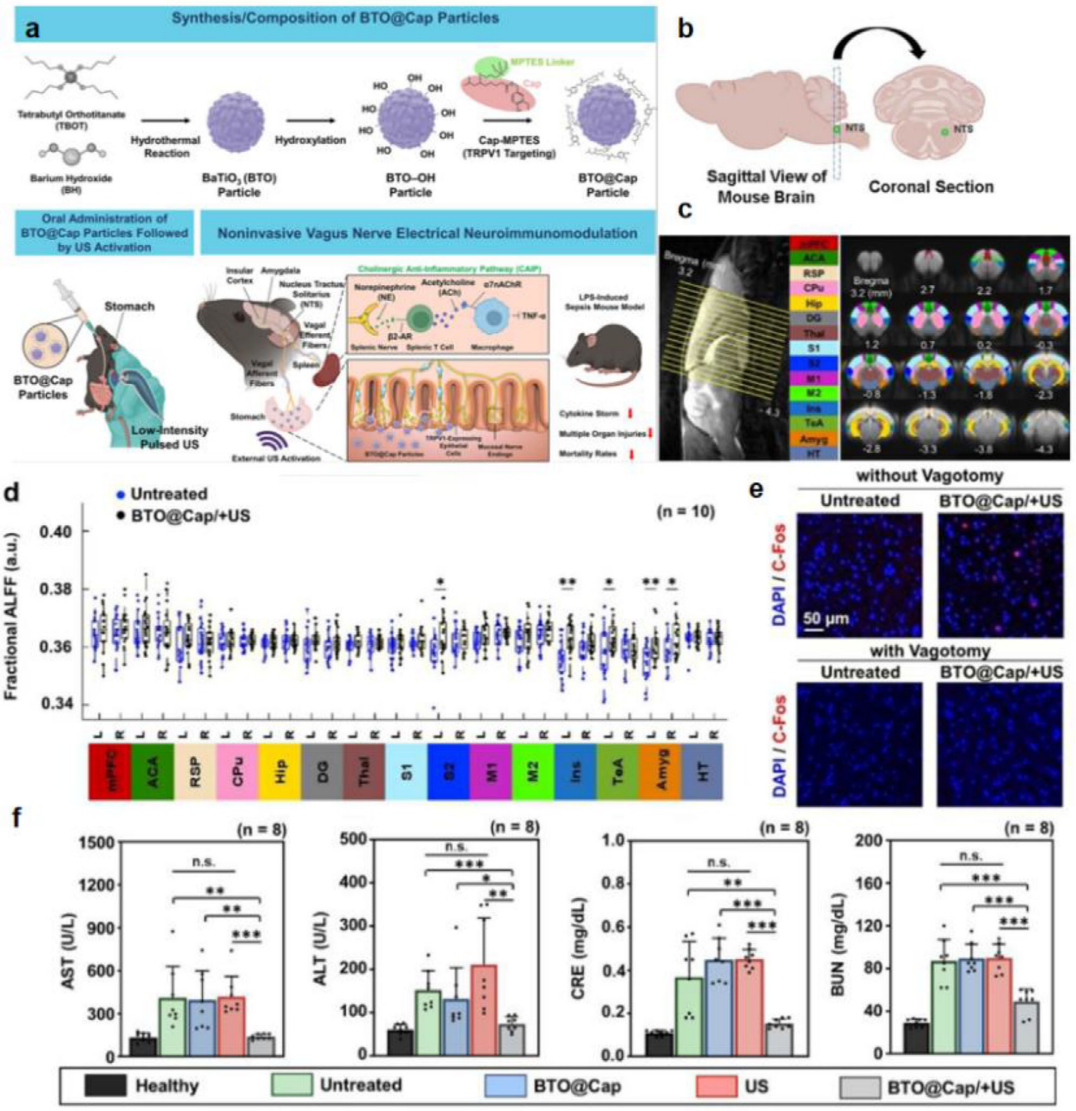
(a) A noninvasive vagus nerve electrical stimulation system for immune modulation and its operating mechanism. (b) Schematic diagrams illustrating the location of the NTS in the brainstem. (c) Illustration of the selected brain regions for analyzing rs‐fMRI data. (d) Results of fALFF analysis in the selected brain regions of the two mouse groups. (e) Immunofluorescence staining images depicting c‐Fos expression in the area defined as the NTS in septic mice, without or with vagotomy, before and after treatment with BTO@Cap/+US. (f) Serum levels of AST, ALT, CRE, and BUN collected from healthy mice and septic mice after different treatments. Reproduced with permission [276]. Copyright 2025, American Chemical Society.

Additionally, researchers have developed a piezoelectric nanogenerator (BTO@Cap) attached to the gastric mucosa to achieve non‐invasive treatment of sepsis (see Figure 41). These nanoparticles can target transient receptor potential vanilloid 1 (TRPV1) and generate electrical pulses when activated by low‐intensity pulsed ultrasound. The electrical pulses stimulate afferent fibers of the vagus nerve, regulate the neuroimmune network by controlling the cholinergic anti‐inflammatory pathway (CAIP), and promote the release of acetylcholine (ACh). The binding of ACh to the α7 nicotinic acetylcholine receptor (α7 nAchR) on macrophages reduces the production of pro‐inflammatory cytokines, thereby inhibiting inflammation [276].

BTO@Cap piezoelectric particle nanogenerators specifically bind to TRPV1 receptors on gastric epithelial cells and the terminals of vagal afferent fibers via capsaicin (Cap). When low‐intensity pulsed ultrasound irradiates the stomach, barium titanate (BTO) piezoelectric particles generate mild electrical pulses that stimulate vagal afferent fibers in the gastric wall. These signals are transmitted upward to the nucleus tractus solitarius (NTS) in the brainstem, activating vagal efferent fibers and initiating the cholinergic anti‐inflammatory pathway (CAIP) [276]. In contrast, polyacrylamide/graphene conductive hydrogels generate electrical signals upon ultrasound stimulation. Through their programmable structure, these hydrogels regulate signal intensity and frequency (adapting to the needs of different inflammatory stages of sepsis) and wirelessly transmit the signals to the vagus nerve. This process inhibits the release of pro‐inflammatory cytokines from immune cells via the CAIP [110].

Traditional therapeutic drugs such as the antibiotic ceftriaxone have side effects and may induce drug resistance, while conventional neural electrical stimulation is associated with nerve‐related side effects, including pain and paralysis. In contrast, the aforementioned nanogenerators adapt to the dynamic inflammatory microenvironment of sepsis and exhibit high stimulation precision.

Additionally, non‐pharmacological treatment strategies, such as photodynamic therapy (PDT), photothermal therapy (PTT), and acoustic therapies, are being developed to address a wide range of conditions linked to infections, inflammation, and immune system dysfunction [370]. These therapies utilize light, sound, or electrical stimulation to target microbial growth, tissue regeneration, and inflammation, without the risk of damage to surrounding healthy tissues. For instance, acoustic power therapy and the generation of reactive oxygen species (ROS) through piezoelectric effects have been successfully employed to combat microbial infections in deeper tissues, presenting a viable alternative to traditional antibiotics and reducing the risk of antimicrobial resistance (AMR). Researchers have developed a single‐electrode TENG skin patch using molybdenum disulfide (MoS_2_) and methacryloyl (GelMA) hydrogel. MoS_2_ exhibits electrical conductivity and excellent photothermal conversion performance, while GelMA shows good biocompatibility. The TENG has achieved a peak‐to‐peak voltage output of 48.80 V and a current output of 0.57 µA [371].

In addition, researchers have developed a self‐powered polylactic acid (PLA) triboelectric nanogenerator (TENG) that synergistically utilizes photothermal therapy and electrical stimulation to promote wound healing. The multiple dynamic bonds in the resulting ionomer dressing endow it with high stretchability (>2000%), self‐healing properties, easy recoverability, and electrical conductivity. Through the combination of photothermal therapy and real‐time electrical stimulation, the PLA ionomer/TENG system can effectively promote angiogenesis, collagen deposition, epithelial reformation, and tissue regeneration, thus accelerating wound healing within a relatively short period (11 days) [370]. These studies provide a new approach for self‐powered wearable electronic devices in the field of wound healing, and also highlight their potential as advanced sensing systems, allowing patients to receive continuous treatment without the need for bulky equipment.

Furthermore, innovations in nanogenerator‐based systems have also paved the way for new therapeutic interventions in areas like tissue engineering and regenerative medicine. Devices like friction nanogenerators, capable of converting mechanical energy into electrical energy, can be used to promote cellular repair processes in tissues affected by neuroimmune disorders. These devices can be integrated into therapeutic platforms designed for the treatment of chronic inflammatory diseases, such as rheumatoid arthritis and multiple sclerosis, where immune dysregulation plays a significant role in disease progression. By stimulating tissue and immune responses at a localized level, these devices can help control inflammation and enhance the healing of affected tissues.

**FIGURE 42:**
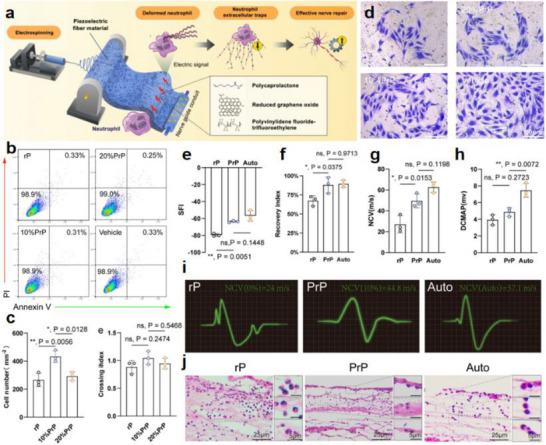
(a) Schematic illustration of electrospun fibers made from piezoelectric materials that produce tiny electrical stimuli and regulate the immune state of neutrophils. (b) Schwann cell apoptosis measured by flow cytometry. (c) Quantitative analysis of the number of cells that passed through the Transwell pores. (d) Assessment of cell migration ability. Scale bars  =  50 µm. (e) Quantitative analysis of the sciatic nerve index. (f) Quantitative analysis of the pressure recovery index. (g) Quantitative analysis of the nerve conduction velocity (NCV) and (h) distal compound motor action potential (DCMAP). (i) Action potential waveform of the sciatic nerve. (j) Neutrophils in the epineurium. Scale bars  =  25 µm. Reproduced with permission [372]. Copyright 2025, Springer Nature.

In the context of peripheral nerve injury (PNI), various cells within neural tissues attempt to migrate toward nerve stumps. However, disruption of the immune microenvironment following PNI often becomes a critical obstacle to nerve regeneration. To address this, researchers developed a self‐powered neural bridging scaffold by incorporating poly (vinylidene fluoride‐trifluoroethylene) (P(VDF‐TrFE)) and reduced graphene oxide (rGO) nanoparticles into a polycaprolactone (PCL) substrate (see Figure 42). The complex cellular migration activities induce minute deformations on the scaffold surface, generating electrical signals. The resulting microenvironment created by the scaffold restricts the excessive inflammatory response of neutrophils after PNI, thereby promoting the regeneration of damaged peripheral nerve tissues [372].

**FIGURE 43:**
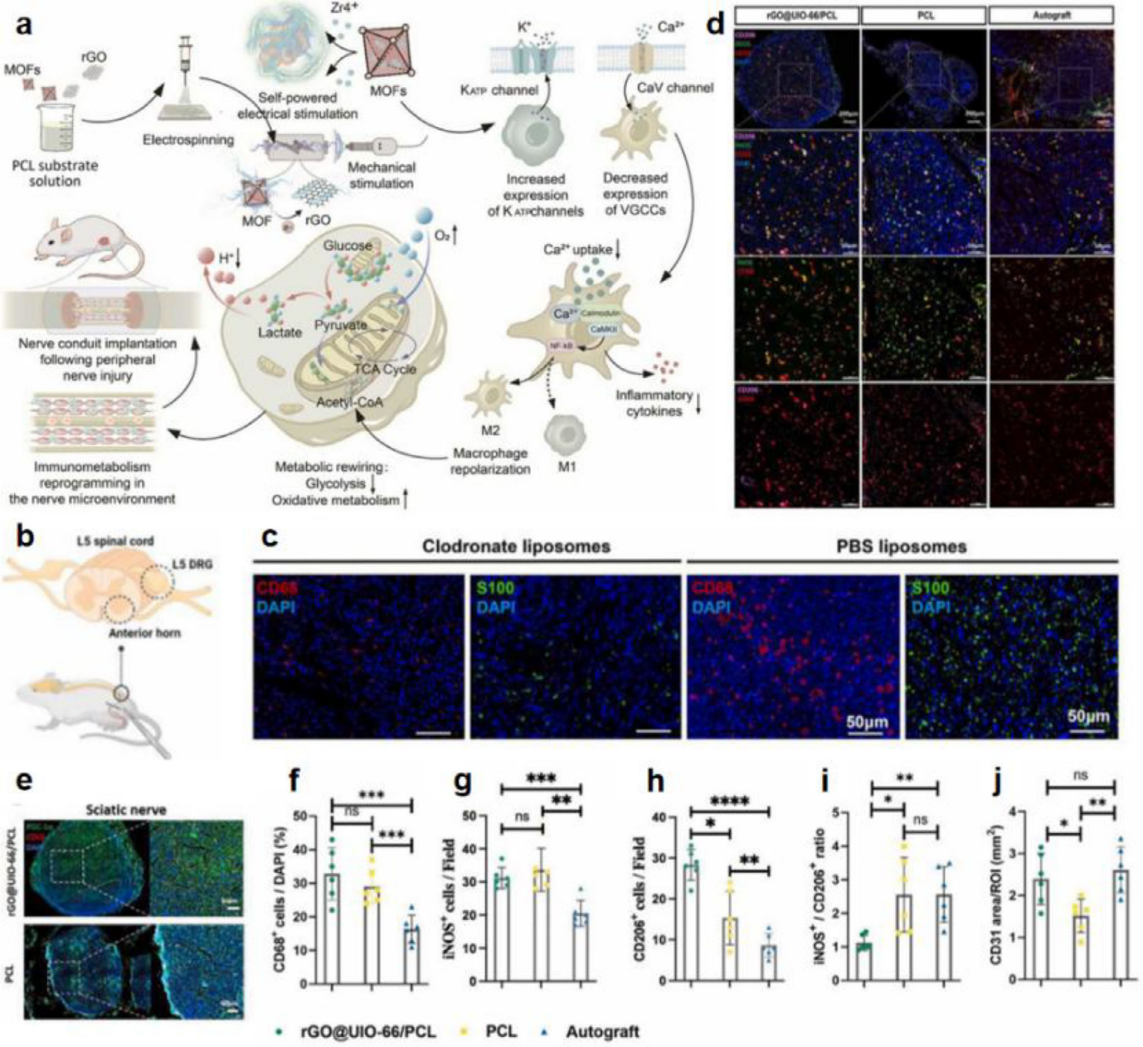
(a) A scheme illustrating the fabrication of rGO@UIO‐66/PCL scaffolds and the mechanism of immunometabolism reprogramming. (b) Schematic illustration of the secondary responses in L4‐L5 DRGs and spinal cord anterior horn following sciatic nerve injuries. (c) Macrophage depletion damaged the outcome of nerve repair as demonstrated by the immunostaining of CD68 (red) and S100 (green) for nerve bridge within rGO@UIO‐66/PCL scaffolds. Scale bar = 50 µm. (d) Macrophage polarization and distribution within conduits as characterized by immunofluorescent staining for CD68 (red), iNOS (green), and CD206 (pink). Cell nuclei were stained with DAPI (blue). Scale bar = 50 µm. (e) Double immunostaining of CD68 (red) and PGC‐1α (green) in nerve transverse sections. Scale bars = 50 µm. (f‐j) Quantitative analysis of CD68‐positive cells infiltration, iNOS‐positive cells infiltration, CD206‐positive cells infiltration, the ratio of M1/M2 macrophages, and neo‐vessel formation, as determined from transverse nerve sections. *p < 0.05, **p < 0.01, ***p < 0.001 and ns, no significant difference. Reproduced with permission [373]. Copyright 2023, Elsevier.

Peripheral nerve injury (PNI) is a common condition in limb trauma. Based on this, researchers have designed a bio‐adaptive neural interface for nerve repair based on UiO‐66‐NH_2_(Zr) and rGO nanoparticles, which can generate and convert electric charges under ultrasonic stimulation to achieve electro‐mechanical conversion (see Figure 43). Treatment with rGO@UiO‐66/polycaprolactone (PCL) scaffolds induces a phenotypic shift of macrophages from the pro‐inflammatory M1 type to the anti‐inflammatory M2 type, and reprograms nerve tissue metabolism from glycolysis‐dominant to oxidative phosphorylation‐dominant. After 8 weeks of in vivo implantation, significant improvement in nerve regeneration was observed [373].

The piezoelectric signals of polyvinylidene fluoride (PVDF)‐based nanogenerators (PVDF‐TrFE/rGO/PCL) can downregulate the expression of CD66b and myeloperoxidase (MPO) on the surface of neutrophils, reducing the release of DNA‐histone complexes from neutrophil extracellular traps (NETs). Inhibition of NETs not only avoids their physical barrier effect on nerve regeneration but also decreases the secretion of NETs‐associated inflammatory factors (e.g., interleukin‐6 [IL‐6], CXCL8), synergizing with macrophage polarization to reshape the immune microenvironment [372]. Under ultrasound stimulation, metal‐organic framework (MOF)‐based nanogenerators (rGO@UIO‐66/PCL) form a built‐in electric field, which enhances the opening of ATP‐sensitive potassium (KATP) channels and promotes K^+^ efflux, leading to hyperpolarization of the macrophage membrane. Membrane hyperpolarization further inhibits the opening of voltage‐gated calcium channels (VGCCs) and reduces Ca^2^^+^ influx, resulting in insufficient activation of calmodulin (CaM) and subsequent inhibition of the phosphorylation of calmodulin‐dependent protein kinase II (CaMKII) [373]. Compared with methylcobalamin (a drug therapy), nanogenerators do not induce side effects such as pain hypersensitivity, nor do they exhibit hepatotoxicity or nephrotoxicity, and can actively reshape the immunometabolic microenvironment. In contrast, standard electrical stimulation relies on external power sources, is invasive, and is prone to causing infections and tissue adhesion.

Beyond the more commonly recognized applications in neurodegenerative diseases, cancer, and autoimmune disorders, the integration of nanotechnology, electrical stimulation, and photothermal therapies holds the potential to revolutionize the treatment of a broad range of neuroimmune and chronic inflammatory conditions. These therapies offer a combination of precision, safety, and versatility, which could significantly improve clinical outcomes and reduce side effects in patients suffering from these complex disorders.

### 4.4 Technological Synergies in Neuroimmune Engineering

Neuroimmune engineering represents a groundbreaking field that enables the precise modulation of the neuro‐immune system through the synergistic use of multidisciplinary technologies. This approach integrates cutting‐edge advancements in bioelectronic medicine, artificial intelligence (AI), and nanotechnology, offering a more comprehensive and effective way to monitor, control, and enhance both neural and immune responses in real‐time [374]. The fusion of these technologies allows for unprecedented levels of precision in treating a wide range of neuroimmune‐related conditions, from chronic pain to neurodegenerative diseases and autoimmune disorders.

The integration of bioelectronic medicine into neuroimmune engineering is a key factor in achieving real‐time monitoring and regulation of neural signals and immune responses. Through the use of implantable or wearable devices, this technology can continuously collect and analyze data from both the neural and immune systems [152]. For example, electrical stimulation and optogenetic techniques can be employed to precisely modulate the activity of neural circuits and immune cells. By enabling such targeted interventions, this system allows for dynamic closed‐loop regulation, where the system can adjust itself in response to ongoing changes in neural or immune activity. Moreover, these techniques facilitate biomimetic simulation of neuro‐immune interfaces, mimicking natural interactions between the nervous and immune systems, thereby enhancing therapeutic efficacy.

AI‐driven adaptive control systems further augment the power of neuroimmune engineering by creating dynamic feedback loops that analyze complex neuroimmune signals. These systems enable intelligent and personalized regulation of both the nervous and immune systems, ensuring that treatments are tailored to the specific needs of individual patients. AI algorithms play a pivotal role by integrating a diverse array of data sources, including neural signals (e.g., EEG, functional near‐infrared spectroscopy or fNIRS), immunomics (such as single‐cell RNA sequencing), and metabolomics. This integrated data forms the basis for building highly accurate, disease‐specific regulatory models [375]. These models are used to predict how neural and immune systems will respond to various interventions, providing a foundation for precision treatment strategies.

For instance, deep learning models have been developed to optimize high‐frequency electrical stimulation parameters for patients with Parkinson's disease. These models analyze the relationship between electroencephalographic (EEG) activities and cutaneous sympathetic nerve responses to enhance therapeutic outcomes, such as improving motor symptoms. Additionally, reinforcement learning algorithms are capable of adjusting therapeutic strategies in real time, based on continuous feedback from the patient [376]. This dynamic, adaptive system ensures that treatments are continually refined to match the evolving needs of the patient, leading to more effective management of symptoms and improved patient outcomes.

The potential applications of neuroimmune engineering extend to a wide array of neuroimmune disorders, such as chronic inflammation, neurodegenerative diseases (e.g., Alzheimer's and Parkinson's diseases), and autoimmune diseases. In these cases, the combination of bioelectronic medicine, AI, and advanced nanomaterials offers powerful new treatment options. For example, the ability to use real‐time feedback loops for adjusting therapeutic strategies in response to the body's dynamic conditions could revolutionize the way chronic conditions are managed. Similarly, integrating AI with electroceuticals and nanosystems can lead to highly specific treatments that not only reduce inflammation or protect neuronal function but also promote healing and tissue regeneration [377, 378].

The synergy of these advanced technologies is leading to a new era in precision medicine. By providing real‐time, data‐driven interventions, neuroimmune engineering can improve patient outcomes, reduce side effects, and enhance the quality of life for individuals suffering from complex conditions. These innovations are not only contributing to more personalized therapies but also driving the development of new, minimally invasive treatment options that can be adapted on demand to suit each patient's unique needs. As the field continues to evolve, we can expect even more refined and effective treatments that will transform the management of neuroimmune disorders, offering hope for patients where traditional therapies have often fallen short.

#### 4.4.1 Convergence with Bioelectronic Medicine for Precision Modulation

Bioelectromedicine is an emerging interdisciplinary medical field that integrates electronic technologies with biological systems to regulate neural signals and treat various diseases. This field plays a pivotal role in the evolving domain of neuroimmunological engineering, offering sophisticated means to precisely regulate neuroimmune processes. By leveraging bioelectromedicine, researchers can develop miniature, implantable, or wearable electronic devices capable of real‐time monitoring and regulation of neural signals. These technologies provide dynamic interventions tailored to the unique needs of patients, promoting highly effective, personalized treatments [379].

A key application of bioelectromedicine in neuroimmunological engineering is its use in Deep Brain Stimulation (DBS). DBS electrodes, implanted in the brain, continuously monitor neural signals such as β waves (13‐30 Hz) and can adjust the frequency of electrical stimulation dynamically. This approach has been shown to significantly improve motor symptoms in Parkinson's disease patients. Similarly, the closed‐loop DBS system, like the NeuroPace RNS, uses electrocorticogram (ECoG) signals to detect epileptic seizures' precursors. The system then triggers electrical impulses to suppress abnormal discharges, reducing seizure frequency by up to 70% [379].

Another prominent application of bioelectromedicine is Vagus Nerve Stimulation (VNS), which has demonstrated therapeutic benefits for various conditions. In animal models, VNS has been shown to enhance vagal nerve activity, thereby reducing pro‐inflammatory cytokines such as TNF‐α and IL‐6, which are key drivers of inflammation in conditions like colitis [141]. Moreover, transcranial alternating current stimulation (tACS), with a frequency of 40 Hz, has been found to induce γ oscillations, reducing β‐amyloid deposition and improving cognitive function in mice. For attention deficit hyperactivity disorder (ADHD) in children, wearable flexible electroencephalogram (EEG) sensors can enhance θ waves (4‐8 Hz) through real‐time feedback training, improving attention span.

Bioelectromedicine's versatility extends beyond electrical stimulation to include photostimulation techniques like optogenetics and near‐infrared light. These technologies regulate neural pathways involved in immune responses, including the vagus nerve, hypothalamic‐pituitary‐adrenal axis, and gut–brain axis [380, 381, 382]. For example, optogenetic activation of Channelrhodopsin‐2 (ChR2) in murine T cells, using blue light, potently suppresses IL‐17 secretion and mitigates neuroinflammatory responses [383]. On the other hand, near‐infrared light activation of Opsin 3 (OPN3) in tumor‐infiltrating lymphocytes (TILs) significantly enhances the cytotoxic activity of CD8^+^ T cells, boosting anti‐tumor immune responses [384]. This ability to precisely control immune responses through electrical and light‐based technologies facilitates both the inhibition of excessive immune activity and the enhancement of immune defense mechanisms, depending on the clinical need.

The ability to precisely regulate the neuroimmune system opens the door to personalized treatments. For autoimmune diseases, bioelectronic devices can continuously monitor a patient's neuroimmune status, adjusting stimulation parameters based on individual differences. This allows for real‐time, personalized immune modulation that improves treatment efficacy while minimizing side effects. The integration of bioelectromedicine into clinical settings offers the potential for highly tailored, dynamic therapeutic approaches that address the complexities of individual patient needs.

Moreover, the collaboration of multiple advanced technologies further enhances the capabilities of bioelectromedicine in neuroimmunological engineering. Combining gene editing tools like CRISPR/Cas9, optogenetics, and nanotechnology enables multi‐level regulation of the neuroimmune system [385, 386, 387]. Gene editing technologies allow for precise modification of immune cell functionality or neuronal excitability at the genetic level. Optogenetics, using light‐sensitive proteins like ChR2 and Halorhodopsin (NpHR), enables the spatiotemporal activation or inhibition of specific neural pathways, allowing for real‐time manipulation of neuroimmune circuits with high precision [388, 389, 390]. Nanotechnology plays a dual role: as a delivery vehicle for gene‐editing tools and optogenetic constructs to enhance tissue specificity, and as a photothermal/photoacoustic transducer to amplify regulatory effects via energy conversion at the nanoscale.

This integration of advanced technologies enables a more comprehensive intervention in the neuroimmune process. For example, in the treatment of neurodegenerative diseases, bioelectronic devices can monitor neuroinflammatory responses and modulate immune cell activity through electrical stimulation, potentially slowing disease progression. Similarly, for patients with traumatic brain injury (TBI) or spinal cord injury (SCI), bioelectronic devices can promote synergy between nerve regeneration and immune response, accelerating the rehabilitation process.

In TBI, the disruption of the blood‐brain barrier, glial scar formation, and chronic neuroinflammation create a pathological environment that impedes neuronal regeneration [391]. To address this, flexible graphene‐based sensors implanted into the brain can dynamically monitor neurotransmitter and cytokine levels. When high levels of inflammatory markers are detected, the sensor can trigger electrical pulses via a microelectrode array, which regulates astrocyte calcium signaling. This promotes the transformation of astrocytes from the A1 scar‐forming phenotype to the A2 repair‐promoting phenotype, reducing glial scar proliferation and improving the microenvironment for neuronal repair [392]. This real‐time monitoring and closed‐loop electrical regulation offer a novel strategy for managing neuroinflammatory cascades post‐TBI.

In SCI, bioelectronic devices can overcome the limitations imposed by glial scars, axonal disruption, and immunosuppressive environments [393]. The use of a nanofibrous membrane as an implantable carrier provides a biocompatible, electrically conductive environment. The hydrogel's porous structure supports axonal growth, while the application of pulsed current (100 µA, 10 Hz) activates a local electric field that promotes Schwann cell proliferation and myelination at the injury site [394]. Additionally, electrical stimulation recruits endogenous neural stem cells through chemokine gradient modulation (e.g., the SDF‐1α/CXCR4 axis) and inhibits excessive glial cell activation, which reduces inhibitory matrix deposition. These combined actions create a conducive immune‐neural microenvironment that supports axonal regeneration, offering a multidimensional approach for SCI recovery.

Ultimately, the integration of bioelectromedicine with neuroimmunological engineering offers promising new avenues for treating neurodegenerative diseases, traumatic injuries, autoimmune disorders, and other complex conditions. By harnessing the power of real‐time monitoring, personalized regulation, and advanced technologies like gene editing and optogenetics, this field is set to revolutionize precision medicine, providing tailored solutions for diverse and challenging medical conditions.

#### 4.4.2 AI‐Driven Control Systems for Adaptive Neuromodulation

The application of AI in neuroimmunological engineering offers powerful technical support for developing adaptive neural regulation and control systems. By utilizing AI‐driven control systems, complex neuroimmune data can be analyzed in real time, enabling the dynamic adjustment of regulatory strategies to achieve more effective treatment outcomes. AI technology excels at processing and analyzing vast amounts of neuroimmune data, including neural signals, immune cell activity, and inflammatory factor levels [395]. Through machine learning algorithms, key regulatory nodes within the neuroimmune system can be identified, and the potential impacts of different stimulation methods on the immune system can be predicted. This data‐driven, precise regulation significantly enhances the specificity and effectiveness of treatment.

The AI‐based adaptive neural regulation and control system continuously adjusts regulation parameters based on the real‐time status of patients. For example, in treating autoimmune diseases, the AI system can analyze patients' neural signals and immune response data to optimize the frequency, intensity, and duration of electrical or photostimulation in real time, ensuring optimal immune regulation. This adaptive strategy increases the flexibility and responsiveness of treatments, offering a more personalized approach [396].

Moreover, the AI‐driven control system integrates data from multiple modalities, including neural signals, immune cell activity, gene expression, and metabolic status. This integration allows for the construction of a comprehensive model of the neuroimmune system. Using deep learning algorithms, the complex interaction mechanisms within the neuroimmune system can be uncovered, enabling the design of more effective and targeted regulation strategies. The system can then automatically optimize treatment plans according to individual patient characteristics [375]. For instance, the AI system can recommend personalized stimulation parameters tailored to the specific neuroimmune status of each patient, ensuring precise immune regulation. This approach not only improves treatment efficacy but also minimizes unnecessary side effects.

The potential for AI‐based adaptive neural regulation and control systems in clinical applications is vast. For example, in treating chronic inflammatory diseases such as rheumatoid arthritis, the AI system can provide long‐term, stable disease control by continuously monitoring the patient's neuroimmune status and dynamically adjusting the immune regulation strategy. In neurological diseases like Parkinson's disease or cancer, the AI‐driven control system can slow disease progression and enhance the patient's quality of life by precisely modulating the neuroimmune response [397].

Ultimately, the integration of AI into neuroimmunological engineering opens new avenues for personalized, adaptive treatments that address the complexities of individual patient needs [374]. By continuously analyzing neuroimmune data and adjusting therapeutic strategies in real time, AI has the potential to revolutionize the treatment of a wide range of diseases, offering more effective and tailored therapeutic solutions.

## 5 Addressing Challenges in Clinical Translation

The clinical translation of innovative therapies, particularly in the fields of neuroimmunological engineering and bioelectromedicine, faces several significant challenges. One of the primary obstacles is the complexity of translating laboratory findings into scalable, reproducible clinical applications. The intricate nature of the neuroimmune system requires a comprehensive understanding of its dynamic interactions, which complicates the development of reliable, standardized therapies. Additionally, ensuring the safety and efficacy of new treatments in diverse patient populations poses challenges, as individual variations can significantly influence treatment outcomes [398].

Another key challenge lies in the integration of advanced technologies, such as AI‐driven systems and implantable devices, into existing healthcare infrastructures. While these technologies show promise in preclinical and early‐phase clinical studies, their widespread adoption is hindered by regulatory hurdles, high development costs, and the need for extensive validation. Furthermore, the long‐term effects of novel therapies, especially those involving bioelectronic devices or gene‐editing technologies, are not yet fully understood, raising concerns about their sustainability and potential side effects.

To address these challenges, a multidisciplinary approach is required, involving collaboration between researchers, clinicians, engineers, and regulatory bodies. Rigorous clinical trials, designed to evaluate the safety, effectiveness, and long‐term impacts of these therapies, are essential [399]. Additionally, innovations in manufacturing processes, such as the development of scalable, cost‐effective production methods, will be crucial to ensuring the successful translation of new therapies into clinical practice. By overcoming these obstacles, we can pave the way for the successful implementation of cutting‐edge treatments that have the potential to revolutionize the management of complex neuroimmune disorders.

### 5.1 Biocompatibility and Long‐Term Stability

The application of nanogenerators, particularly in the biomedical field, encounters a range of significant challenges, with biocompatibility and long‐term stability being two of the most critical factors. Biocompatibility refers to the safety and adaptability of materials when they interact with living organisms, which is paramount for the successful integration of nanogenerators in medical applications [400]. These devices are typically composed of a variety of materials, including metals, polymers, and ceramics, which can, unfortunately, trigger immune responses or exhibit cytotoxicity. For example, the implantation of graphene‐based triboelectric nanogenerators (TENGs) subcutaneously in mice has been shown to result in macrophage infiltration, a threefold increase in interleukin‐6 (IL‐6) levels, and the development of chronic inflammation. This inflammatory response led to the formation of a fibrotic encapsulation that inhibited the energy output of the device.

**FIGURE 44:**
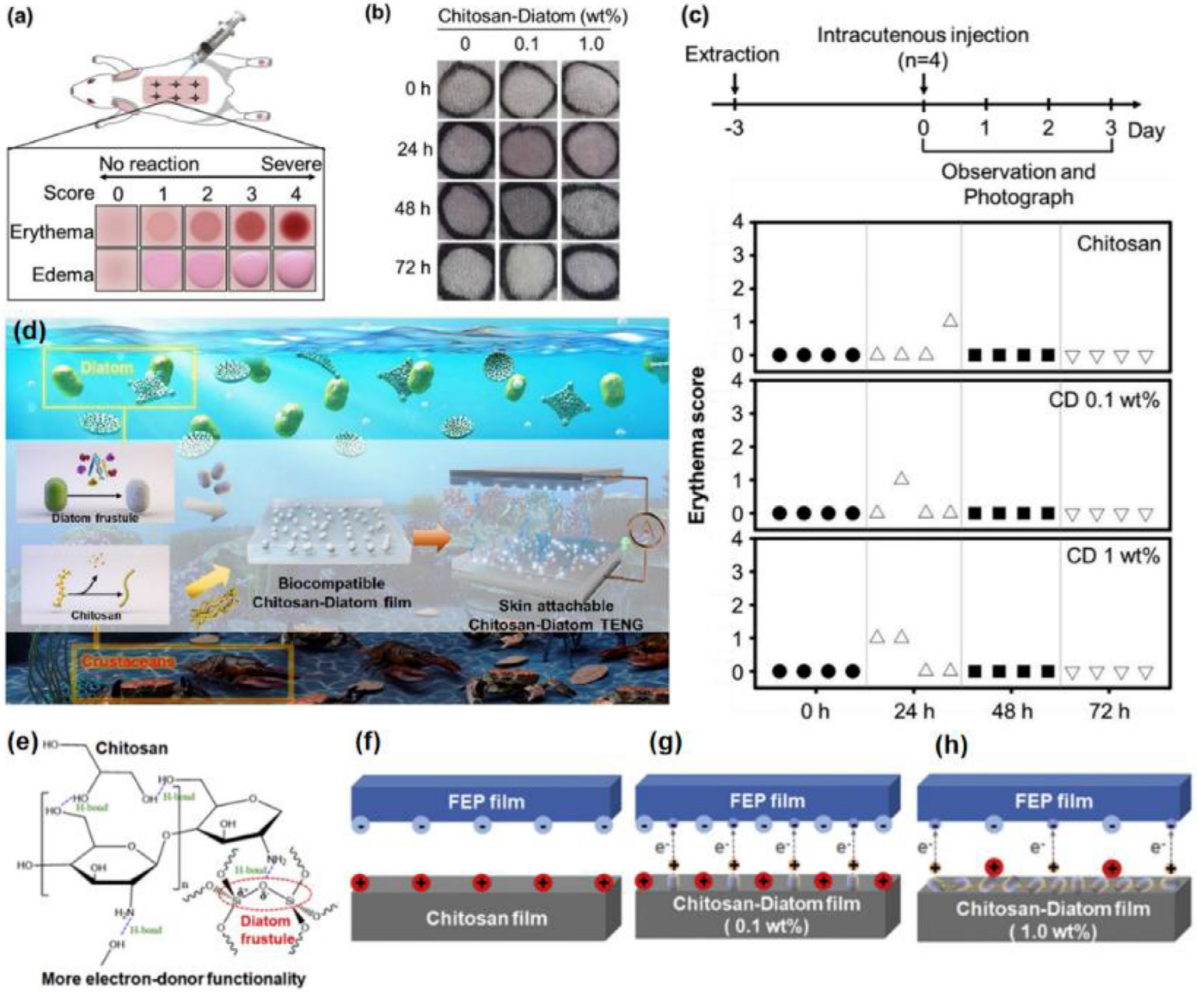
(a) Score index for intracutaneous reactivity, (b) Erytherma reactivity results for 3 days with chitosan and chitosan‐diatom injection, and (c) Ertherma score with 4 samples for 3 days. (d) A chitosan‐diatom TENG for biofriendly and skin‐attachable wearable devices. (e) Chemical structures and electronic interaction between chitosan and diatom frustule. Schematic model of electrical charges in (f) chitosan and chitosan‐diatom film of (g) 0.1 wt% and (h) 1.0 wt% after contact and separation. Reproduced with permission [402]. Copyright 2020, Elsevier.

To improve the biocompatibility of nanogenerators, Researchers used silk as the raw material and exfoliated single‐layer nanoribbons via the sodium hypochlorite/sodium bromide/TEMPO system. These nanoribbons were then fabricated into a nanoribbon film as the triboelectric layer, which was combined with a magnesium conductive layer and a post‐treated regenerated silk fibroin membrane encapsulation layer to construct an all‐silk‐based nanogenerator. Silk itself exhibits excellent biocompatibility, a property that has been verified for the device through Schwann cell culture. This avoids the inflammation caused by conductive layers such as ITO and aluminum foil in traditional devices, making it suitable for implantation in the human body to power devices like cardiac pacemakers [401]. Moreover, researchers have developed a chitosan‐diatom triboelectric nanogenerator (TENG) based on diatom shells and chitosan, which is designed for bio‐friendly and skin‐attachable wearable devices [402]. The naturally porous diatom shells possess a high degree of porosity and can be mass‐produced from the large‐scale marine environment. They can be used as biocompatible additives to significantly alter the electropositivity and surface properties of the chitosan membrane, exhibiting biological affinity and higher output power. For example, the measurement of the time‐averaged power density of the chitosan‐diatom triboelectric nanogenerator is 15.7 mW/m^2^, which is 3.7 times that of the pure chitosan TENG (see Figure 44).

Long‐term implantation of nanogenerators in the body can also result in chronic inflammatory reactions, leading to fibrosis or tissue necrosis. For example, polylactic acid (PLA) and polycaprolactone (PCL) composites, while suitable for short‐term implants, may trigger inflammatory responses when used for prolonged periods. Studies have demonstrated that polyvinyl alcohol (PVA)‐based hydrogels, which exhibit excellent biocompatibility, can swell and undergo structural degradation in vivo, potentially affecting the stable output of nanogenerators [403]. Consequently, evaluating the safety of long‐term interactions between nanogenerators and biological tissues is crucial for determining whether these devices can be safely used for extended periods.

Long‐term stability is another vital consideration for the successful application of nanogenerators in the biomedical field. These devices must maintain their performance over prolonged periods of use to ensure continuous, efficient monitoring and therapeutic function in the human body. The degradation of materials in the in vivo environment can negatively impact the performance and functionality of nanogenerators. For example, the PVDF‐based triboelectric nanogenerator (TENG) experiences a 30% reduction in its β‐phase content at low temperatures (−20°C), which results in a decrease in surface charge density from 45 to 28 µC/m^2^. Similarly, when the temperature exceeds 50°C, the mismatch in the coefficient of thermal expansion between piezoelectric ceramics like PZT and polymer substrates such as PDMS leads to interface delamination, causing a 40% decrease in output charge [404].

To address the drawbacks of self‐healing superhydrophobic triboelectric nanogenerators (TENGs), namely poor mechanical stability and high healing temperature, researchers fabricated two types of TENGs modified with high‐performance coatings. The first type involves spraying imine‐crosslinked polydimethylsiloxane (PDMS)‐based supramolecular polymers and silica nanoparticles to form an I‐PDMS/SiO_2_ coating. This coating retains superhydrophobicity even after 154.1 m of sandpaper abrasion under 3.1 kPa pressure or 10 000 cycles of foot stamping impact, and can spontaneously heal at −30°C. The second type, inspired by the coral structure, loads hydrophobic self‐healing fluorinated polyurethane and polyvinylidene fluoride nanoparticles onto iron foam, resulting in the USSS‐TENG. This device can withstand harsh tests such as 391 meters of sandpaper abrasion and 50 cycles of rolling by a 1.6‐ton automobile, while also possessing low‐temperature self‐healing capability [405]. Both devices ensure the long‐term stable power generation of nanogenerators in complex environments.

**FIGURE 45:**
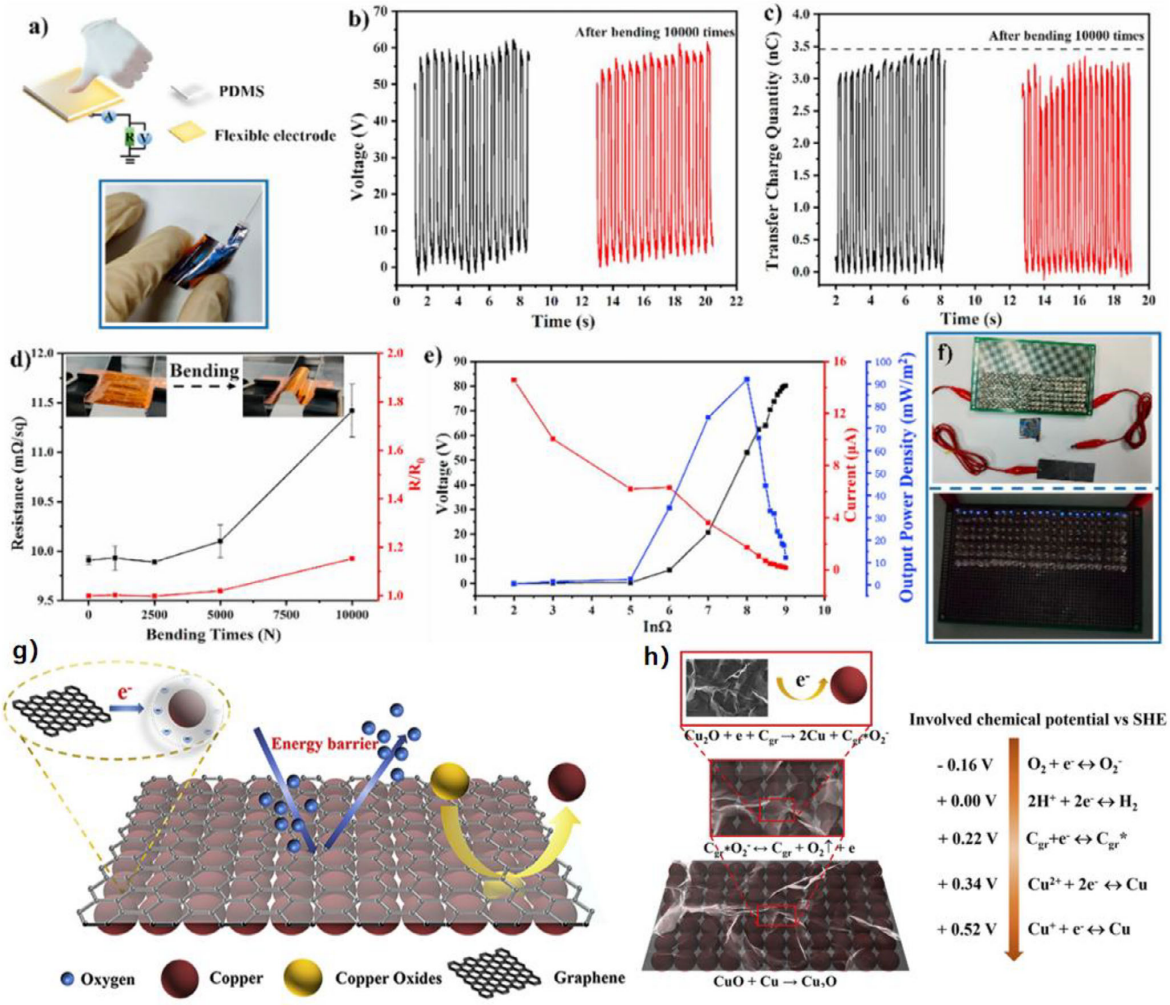
(a) The schematic diagram and optical picture of skin‐based flexible single‐electrode triboelectric nanogenerator. The output characteristics of TENG based on graphene/Cu/PDMS: (b) output voltage; (c) transfer charge quantity. (d) The bending performance of a flexible electrode based on graphene/Cu/PDMS. (e) The variation of output voltage, current, and power density with the external load resistance. (f) LED application: blue LED array powered by TENG. (g) The reasonable mechanism proposed in this paper about the oxidation resistance and deoxidation for the graphene/Cu heterostructure. (h) The involved electron transfer and chemical reactions in this mechanism. Reproduced with permission [406]. Copyright 2020, Elsevier.

To enhance the long‐term stability of nanogenerators, researchers have developed a flexible single‐electrode triboelectric nanogenerator (TENG) device composed of a graphene/copper heterostructure and a PDMS membrane. This electrode is fabricated through electrodeposition and spin‐coating processes. Based on PDMS, a graphene dispersion prepared by physical exfoliation is spin‐coated onto the copper nanostructure. This device is also utilized for energy harvesting in triboelectric nanogenerators (TENGs). The peak value of the output voltage based on the graphene/Cu/polydimethylsiloxane (PDMS) structure is approximately 60 V, and the peak value of the transferred charge quantity is about 2.5 nC, which is equivalent to or better than that of the previously reported triboelectric nanogenerators (TENGs) based on pure PDMS [406]. The bending test shows that the sheet resistance of the flexible electrode and the electrical output characteristics of the triboelectric nanogenerator (TENG) have not significantly deteriorated compared with those of the unbent device, indicating the flexibility and reliability of this TENG based on graphene/Cu/polydimethylsiloxane (PDMS). The experimental data demonstrate that the electron transfer mechanism of the graphene/Cu heterostructure can enhance the stability of the triboelectric nanogenerator (see Figure 45).

Thus, improving the biocompatibility, reducing toxicity, and enhancing the long‐term stability of nanogenerators are essential steps toward making these devices viable for sustained biomedical applications.

### 5.2 Precision Targeting of Neuroimmune Sites

In the field of neuroimmunology, precisely targeting specific neuroimmune sites is critical for achieving effective therapeutic outcomes. The application of nanogenerators (NGs) in this context demands that these devices can accurately locate and act on target cells or tissues, minimizing potential damage to surrounding healthy tissues [407]. However, one of the primary challenges faced by nanogenerators in biomedical applications is insufficient targeting capability. To achieve precise targeting, nanogenerators need to bind to specific cell‐surface markers, such as receptors or antigens. Currently, most nanogenerators lack the ability to selectively recognize these molecules, resulting in low targeting efficiency and diminished therapeutic effectiveness.

Within the central nervous system (CNS), the complexity is further heightened by the heterogeneity among different types of neurons and immune cells, such as microglia and astrocytes. This diversity makes it even more challenging to identify specific targeting markers that would allow nanogenerators to act on their intended cells. Without such markers, nanogenerators are often unable to selectively reach their targets, leading to reduced therapeutic precision. Moreover, the blood‐brain barrier (BBB) presents an additional formidable obstacle for nanogenerators. The BBB, composed of endothelial cells bound by tight junctions, severely restricts the passage of substances, even those with a molecular weight under 500 Daltons [408]. Given that nanogenerators generally exceed 100 nm in size, their ability to penetrate the BBB and reach neuroimmune sites within the brain is greatly hindered, resulting in an exceedingly low concentration of nanogenerators in the brain.

The lack of targeting ligands that can bind to specific transport proteins on the BBB, such as glucose transporters, means that nanogenerators cannot exploit active transport mechanisms to gain entry into the brain. Consequently, the therapeutic potential of nanogenerators for treating neuroimmune disorders remains limited. Additionally, nanomaterials face significant challenges due to biological interactions that hinder their precise localization at target neuroimmune sites. Once inside the body, biomaterials may trigger immune responses or be taken up by non‐target cells, which prevents the nanogenerators from achieving accurate localization. For example, monocytes and macrophages recognize specific structures, such as hydrophobic regions on the surface of nanoparticles, through TLR4 receptors, which activate a phagocytic clearance mechanism. This leads to high phagocytosis rates (over 80%), diminishing the targeting efficiency of nanogenerators and potentially leading to unwanted immune responses [409].

In addition, when nanogenerators enter the bloodstream, serum proteins such as albumin and fibrinogen often adsorb to their surface, forming a “protein corona”. This corona can be recognized by receptors (e.g., scavenger receptors) on non‐target cells, such as macrophages, which can lead to the non‐specific phagocytosis of the nanogenerator. This further complicates efforts to achieve precise targeting at neuroimmune sites [410]. The microenvironment at neuroimmune sites can also change dynamically due to pathological conditions such as inflammatory responses or tumor microenvironments. These changes can affect the behavior of nanogenerators, making it difficult for them to maintain their targeting capabilities. Most nanogenerators are not designed to adapt to such fluctuations, leading to a loss of effective targeting and a reduction in their overall therapeutic efficacy.

To overcome these challenges, research is ongoing into the development of nanogenerators with advanced targeting strategies. These may include surface modifications that enable the nanogenerators to bind more effectively to specific neuroimmune markers or the use of nano‐engineering techniques to enhance their ability to cross the BBB. Additionally, the design of biocompatible materials that can avoid immune clearance and maintain stability in dynamic microenvironments is crucial for improving the targeting accuracy and long‐term performance of nanogenerators in neuroimmunological treatments. Optimizing these aspects will pave the way for more precise and effective treatments for neuroimmune disorders, such as autoimmune diseases or neuroinflammatory conditions, where accurate delivery to the brain or specific immune cells is essential.

**FIGURE 46:**
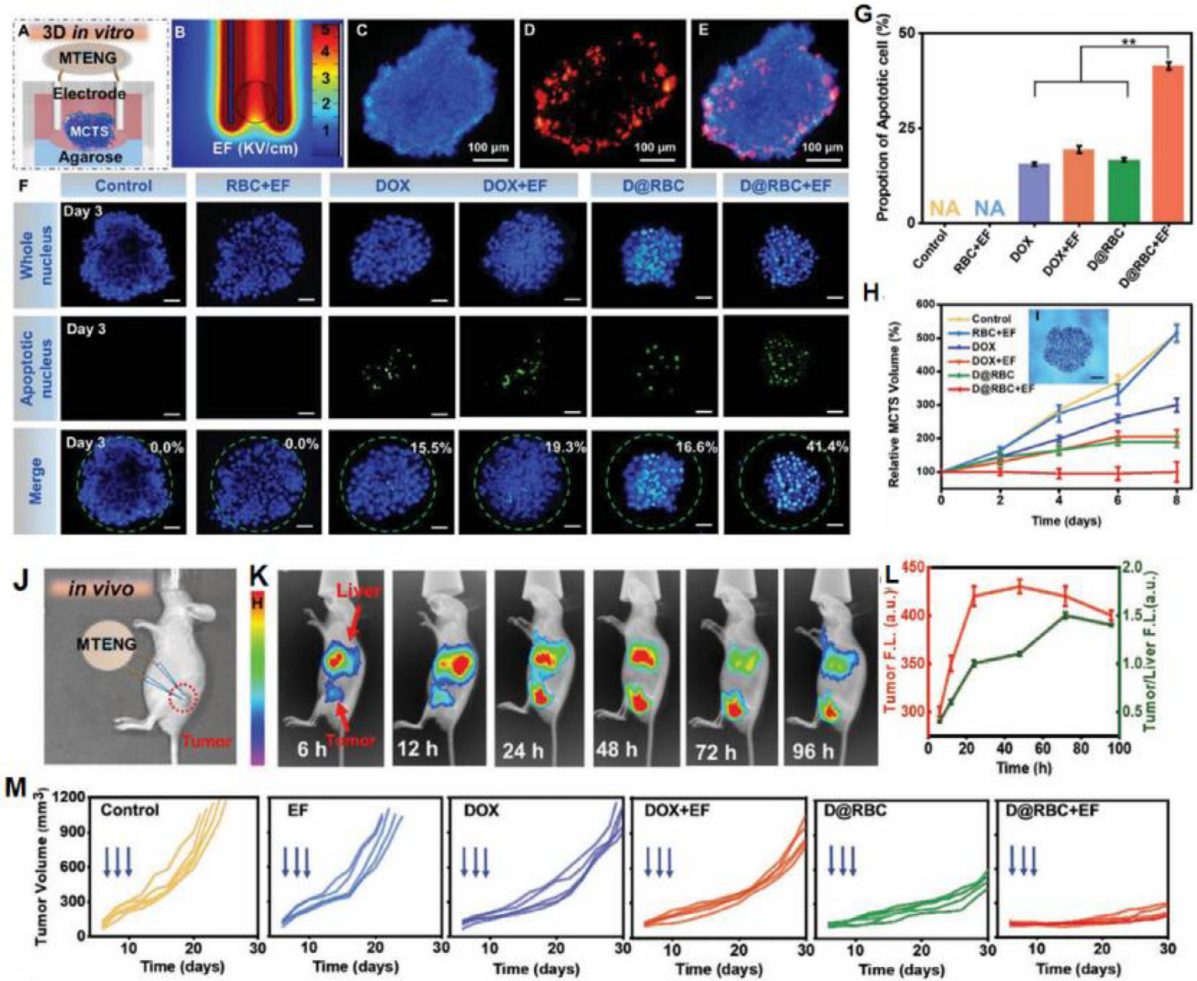
(A) Schematic diagram of 3D electroporation device. (B) Finite element analysis of 3D stimulation device by COMSOL. (C–E) Colocation of (C) HeLa cells and (D) RBCs. (F) Representative fluorescent microscope images of TUNEL assay in cryosections of HeLa MCTS after different treatments for 2 days. Scale bar: 50 µm. (G) Proportion of apoptotic cells in various MCTS groups after 2 days of respective treatments. The data are shown as mean ± SD (*n* = 3). (H) The inhibitory effect of different treatments on the size growth of HeLa MCTS in 8 days (n = 4). *p* values: **p < 0.01 or *p < 0.05. (I) A representative MCTS for size measurement; scale bar: 50 µm. (J) The sketch map of MTENG‐controlled RBC DDS in the tumor‐bearing nude mice. (K) Blood circulation and accumulation to tumors of the D@RBCs obtained by in vivo imaging system at 6, 12, 24, 48, 72, and 96 h, respectively. (L) The tumor fluorescence intensity and the ratio of tumor‐to‐liver fluorescence intensity. (M) The in vivo tumor growth curve; blue arrows indicate the treatment time point: Day 6, Day 8, and Day 10. Reproduced with permission [140]. Copyright 2019, Wiley.

To achieve the precise targeting of the nanogenerator, the researchers developed a novel magnetized triboelectric nanogenerator (MTENG) and used doxorubicin (DOX)‐loaded red blood cells (RBCs) as an anti‐tumor drug delivery system (DDS). The red blood cells have excellent biocompatibility, membrane flexibility, and membrane stability, which can avoid the immune clearance of the body, improve the penetration and localization efficiency of the drug delivery system, and thus enhance the targeting ability [140]. After multicellular tumor spheroids (MCTS) were incubated with red blood cells (RBCs) for 12 hours, the fluorescence microscopy images showed that the red blood cells (in red) had successfully penetrated into the interior of the multicellular tumor spheroids (in blue). This situation is consistent with the enhanced permeability and retention (EPR) characteristics of nanodrug carriers for tumors in vivo. The tumor volume in the D@RBC+EF group controlled by MTENG was the smallest, and it exhibited the best inhibitory effect on tumor growth (see Figure 46).

To enhance the permeation ability of the nanogenerator through the multiple biological barriers of solid tumors, the researchers designed a tumor‐specific nanogenerator PMCS for peroxynitrite (ONOO^−^). It was prepared by loading cisplatin and sodium nitroprusside into poly(D,L‐lactide‐co‐glycolide) polymer vesicles, aiming to improve the drug‐targeted delivery ability and enhance the tumor chemotherapy effect. The body weights of the mice in each group showed no significant changes during the treatment process, indicating that the systemic toxicity of PMCS NPs was low. After the mice were sacrificed on the 15th day, the main organs (heart, liver, spleen, lung, and kidney) and tumors were collected. The tumor weight in the PMCS group was the smallest. Hematoxylin and eosin (H&E) staining showed that the necrotic area of the tumor tissue in the PMCS group was the largest. In the TUNEL assay, the apoptosis level of the cells in the PMCS group was the highest, which was superior to that in the PMS and PMC groups [364]. With the assistance of ONOO^−^, PMCS can not only target the tumor site but also penetrate into the interior of the tumor, overcoming the difficulty of the nanogenerator in breaking through the biological barrier and achieving the precise positioning of the neuroimmune site.

### 5.3 Fine‐Tuning Output Signals for Safe and Effective Modulation

To obtain approval from the US Food and Drug Administration (FDA) or the European Medicines Agency (EMA), two core regulatory principles must be satisfied: the verifiability of therapeutic effects and the controllability of risks. In accordance with the “safety and effectiveness” review criteria, the FDA requires that medical devices produce consistent and predictable therapeutic outcomes under intended use conditions, while controlling patient risks within an acceptable range. In contrast, the EMA places greater emphasis on a “benefit‐risk assessment” framework, mandating that the clinical benefits offered by medical devices significantly outweigh their potential risks [411]. These regulatory requirements are not abstract concepts but rather directly establish clear boundary conditions and technical thresholds for the output signal modulation of our devices. Any optimized design of signal parameters must be fundamentally aimed at ultimately meeting these regulatory standards; otherwise, the device will fail to pass the successive hurdles of preclinical validation and clinical trials.

However, in practical applications, particularly in intelligent systems and the biomedical field, nanogenerators face significant challenges precisely in the control and modulation of output signals—an area focused on by these regulatory requirements. Effective signal control is critical to ensuring the proper operation of nanogenerators, as it directly impacts energy conversion efficiency, signal stability, and system response speed—all of which are core prerequisites for meeting the FDA's requirement of “consistent and predictable effects” and the EMA's criterion of “benefits outweighing risks.” Precise signal modulation enables the accurate operation of devices and real‐time feedback, which is not only crucial in numerous application scenarios such as energy harvesting and biosensing but also directly related to the ultimate success of regulatory review and validation by authorities.

Environmental factors, such as temperature, humidity, and vibration, can significantly affect the performance of nanogenerators. These factors result in instabilities in output voltage and current, which make signal control difficult, reduce the signal‐to‐noise ratio (SNR), and ultimately impair system performance. Research has shown that implantable nanogenerators (NGs), when operating near the heart, experience interference from electromagnetic noise within electrocardiogram (ECG) signals. The frequency range of these ECG signals (0.5–100 Hz) significantly overlaps with the output frequencies of triboelectric nanogenerators (TENGs), which range from 1–10 Hz. This overlap causes signal aliasing errors, exceeding 30%, and hampers the accuracy of the signal [412].

Moreover, the modulation bandwidth of existing piezoelectric energy harvesting circuits is generally limited to less than 1 kHz. This constraint makes these circuits ill‐suited to handle high‐frequency dynamic signals, such as neuronal action potentials, which require real‐time acquisition. The limitations of current modulation techniques restrict their applicability to certain types of nanogenerators, narrowing the scope of their use [413, 414]. Therefore, it is essential to choose appropriate modulation techniques, such as pulse‐width modulation (PWM) and frequency modulation (FM), to optimize the output signal and ensure better performance.

In addition to signal modulation, energy management plays a crucial role in ensuring the effective operation of nanogenerators. The output signals of nanogenerators must be integrated with energy storage systems, such as batteries or supercapacitors, to enable efficient energy storage and release [415]. For instance, when using a DC‐DC boost converter with an efficiency of approximately 85% to convert the pulsed voltage (peaking at 10 V) from a triboelectric nanogenerator (TENG) into a stable 5 V output, the overall system efficiency drops to 35%. The pulsed current output from nanogenerators, with peak values exceeding 10 mA, leads to a significant lag in the ion rearrangement process within supercapacitors, especially those made from activated carbon materials. This results in a 30% reduction in the supercapacitors' cycle life.

Nanogenerators face multiple challenges in the areas of output signal control and modulation, including issues related to signal stability, modulation complexity, energy management, and integration compatibility. By optimizing design strategies, adopting advanced modulation techniques, implementing intelligent energy management systems, and promoting standardization, these challenges can be addressed effectively. Such improvements will enhance the performance and reliability of nanogenerators, making them more suitable for a wide range of applications, particularly in biomedical and intelligent system contexts.

To achieve the modulation of the output signal, the researchers developed a cost‐effective triboelectric nanogenerator in the vertical contact‐separation mode, which uses polyethylene (PE) and polycarbonate (PC) in a conventional digital versatile disc. This low‐cost nanogenerator with a simplified structure is capable of generating an open‐circuit voltage of 215.3 V and a short‐circuit current of 80 µA. The effects of the collision distance and the air gap between the triboelectric layers were also tested within the ranges of 3 to 9 cm and 0.25 to 1 cm, respectively, and it was determined that 0.5 cm is the optimal air gap [416].

Starting from the endpoint evidence required for regulatory approval (e.g., consistency reports, long‐term animal study data, and clinical endpoint indicators), we inversely define the Key Performance Parameters (KPPs) that need to be characterized and optimized, as well as their acceptable ranges. Specifically, we need to establish the concept of a design space, incorporating parameters such as output signal variability, long‐term stability, and device‐tissue interaction into design considerations. These parameters must be demonstrated to lie within acceptable ranges through methods including design verification testing, process validation, and accelerated aging testing [417]. Meanwhile, long‐term in vivo studies should be conducted to establish the dose‐time‐effect relationship between signal attenuation and therapeutic efficacy, and preclinical studies are required to quantify device‐tissue interaction—validating that the therapeutic window (between efficacy and safety) remains sufficiently wide within the expected range of individual variability. Ultimately, through this progressive logic of “regulatory requirements → scientific challenges → technical solutions,” we can construct a complete and actionable clinical translation pathway, ensuring the smooth transition of the device from laboratory research to clinical application.

### 5.4 Human Safety and Biocompatibility Considerations

The safe application of nanogenerators in neuroimmune regulation necessitates a comprehensive safety assessment framework. This framework must rigorously evaluate not only the potential for neurotoxicity and unintended immunomodulation within the central nervous system but also the systemic biocompatibility and long‐term fate of the devices post‐degradation, as these factors are intrinsically linked to overall biological safety. Furthermore, the environmental impact of nanomaterials used in these generators cannot be overlooked, as it represents an extension of the safety paradigm from the individual patient to the broader ecosystem.

Nanomaterials may cross the blood‑brain barrier, peripheral nerve terminals, or placental barrier to enter the central nervous system, where they interact with neurons and glial cells, leading to neurotoxic effects such as oxidative stress, neuroinflammation, DNA damage, and cell death [418]. At the cellular level, nanomaterials can be taken up by neurons and glial cells, triggering apoptosis via physical damage to lysosomes or induction of endoplasmic reticulum stress. At the molecular level, nanomaterials can activate inflammatory signaling pathways such as MAPK/NF‑κB, promoting the release of pro‑inflammatory cytokines (e.g., TNF‑α, IL‑1β, IL‑6), while also generating excessive reactive oxygen species (ROS) through redox cycling, resulting in mitochondrial dysfunction and DNA damage. Nanogenerators can elicit immune responses through multiple mechanisms: first, the surface properties of nanomaterials (e.g., charge, hydrophobicity) can be recognized by pattern recognition receptors (PRRs) on immune cells (such as macrophages and dendritic cells), activating Toll‑like receptor (TLR) signaling pathways and inducing the release of inflammatory factors; second, the “nanoparticle protein corona” formed in vivo (through association with proteins, lipids, and other biomolecules) can alter the immunorecognition properties of nanomaterials, affecting phagocytosis and antigen presentation by immune cells; third, nanomaterials may activate the NLRP3 inflammasome, promoting the maturation and release of IL‑1β and IL‑18 and inducing pyroptosis; furthermore, certain nanomaterials can induce oxidative stress and inflammatory responses via the TLR4/NF‑κB pathway, driving microglial polarization toward the pro‑inflammatory M1 phenotype, ultimately leading to synaptic dysfunction in neurons [419].

Safety is a critical consideration in the application of nanogenerators, particularly in fields involving human health and the environment. For example, after the in vivo degradation of implantable silver (Ag) nanowire generators, Ag^+^ ions are transported via the bloodstream to the liver. These ions inhibit the activity of mitochondrial respiratory chain complexes in hepatocytes, with inhibition rates consistently exceeding 40%. This disruption triggers oxidative stress and results in significant hepatotoxicity. Moreover, unmodified nanogenerators, lacking appropriate surface modifications, can trigger the release of complement components C3a and C5a, leading to immunological responses and allergic reactions, with clinical incidence rates as high as 15% [420]. Therefore, it is crucial for nanogenerators to exhibit favorable biocompatibility to reduce cytotoxicity and minimize adverse immune reactions.

The materials released by nanogenerators into the environment also pose potential hazards to aquatic ecosystems, soil, and organisms. Graphene quantum dots (GQDs) also pose a substantial threat to aquatic ecosystems. They are prone to bioaccumulation in the aquatic food chain, with a bioconcentration factor (BCF) exceeding 1000. Once incorporated into fish, GQDs interfere with the activity of key liver enzymes, such as cytochrome P450 (CYP450), which reduces detoxification capacity by 60%, potentially triggering adverse ecological effects [421]. Research has shown that discarded TiO_2_‐based nanogenerators release nanoparticles into the soil at concentrations above 100 mg/kg, which adsorb onto the surface of *Rhizobium* bacteria, disrupting nitrogen fixation and reducing soil fertility by 30% [422].

The regulatory framework for nanogenerators (NGs) faces significant challenges due to the absence of unified international standards, which creates barriers to market access and raises concerns regarding both human safety​ and environmental protection. Current testing protocols fail to accurately assess key risks. For example, the EU's REACH regulation requires cytotoxicity testing (e.g., MTT assay), but does not account for the slow‐release effects of immobilized nanoparticles in NGs, leading to toxicity underestimations of over 50%. Similarly, the U.S. EPA's ecological risk assessments focus on free nanoparticles, overlooking the potential environmental impact of nanoparticles released during the degradation of encapsulation materials like PDMS—a gap that undermines the accuracy of environmental half‐life predictions.

Regulatory disparities across regions further complicate compliance. The EU's General Product Safety Regulation limits nanoparticle concentration in NGs to <0.1 wt%, whereas China's guidelines impose no such limit, forcing exporters to pursue dual certifications at a 30% cost increase. Divergent medical device classifications also pose challenges: the U.S. FDA classifies implantable NG sensors as Class III devices requiring clinical trial data, while Japan's PMDA may allow conditional approval based on in vitro data. These inconsistencies not only increase economic and administrative burdens but also delay the translation of NG technologies into clinically and environmentally safe applications [413].

The international nature of nanogenerator production and use also presents a challenge in establishing effective global regulatory frameworks to ensure product safety and compliance. Additionally, public awareness and acceptance of nanogenerators pose significant challenges. Ethical concerns related to privacy and health monitoring in nanotechnology applications further necessitate the consideration of comprehensive regulatory policies. By establishing robust safety assessment standards, strengthening regulatory frameworks, and enhancing public education, these challenges can be effectively addressed to promote the safe and responsible application of nanogenerators.

## 6 Future and Outlooks

This review has provided an in‐depth exploration of the role of nanogenerators in modulating neuroimmune responses, a critical aspect of neurological health and disease. It highlighted the intricate nature of neuroimmunity and its significant influence on disease progression, emphasizing the potential of nanogenerators to offer innovative therapeutic strategies. We examined the mechanisms through which nanogenerators interact with the neural immune system, focusing on their ability to stimulate neural circuits, activate immune responses, and ultimately contribute to the treatment of a range of neurological disorders, such as epilepsy, Parkinson's disease, Alzheimer's disease, and stroke. Additionally, the review discussed the synthesis strategies, classification frameworks, and milestones in the development of nanogenerators, along with the challenges that still need to be addressed.

Looking forward, the future of nanogenerator‐based neuroimmunomodulation will likely be shaped by advancements in both nanotechnology and neuroscience. One promising direction involves the continued refinement of nanogenerator materials to improve their biocompatibility, stability, and specific targeting capabilities within the neural environment. Optimizing these aspects will enhance the precision with which nanogenerators can modulate neuroimmune processes, thus minimizing unwanted side effects. Another key development will be the integration of nanogenerators with advanced AI systems, allowing for real‐time, personalized adjustments in the modulation of neuroimmune responses. This could pave the way for more effective and adaptive treatments for neurological conditions.

Moreover, the application of nanogenerators to stimulate the vagus nerve and activate the brain's immune system represents an exciting frontier in the therapeutic landscape. Future research could focus on further exploring the mechanisms through which this stimulation occurs and its effects on neuroimmune balance. This would enable clinicians to leverage nanogenerators for a wider range of neurological diseases, not only to treat existing conditions but also to prevent or slow their progression. In parallel, the development of non‐invasive, wireless nanogenerators could offer enhanced patient comfort, improving accessibility and long‐term usability of these devices in clinical settings.

Despite the promise of nanogenerators, several challenges remain that could hinder their broader clinical application. One major issue is the limited understanding of the long‐term effects of nanogenerators on neural and immune system interactions. While short‐term studies show promise, the long‐term safety and efficacy of these devices need further investigation. Additionally, regulatory frameworks for the approval of nanogenerator‐based therapies are still in the early stages. The complexity of nanogenerator materials and their interactions with biological systems calls for robust, standardized testing and safety protocols.

Addressing these challenges will require a multi‐disciplinary approach, combining nanotechnology, neuroscience, immunology, and regulatory science. Future research should focus on expanding the knowledge of the long‐term biocompatibility and safety profiles of nanogenerators, particularly in terms of their potential effects on neural circuits and immune cells. Collaborative efforts among academic researchers, clinicians, and regulatory bodies will be essential in developing clear guidelines for the clinical translation of nanogenerators. Furthermore, advancing materials science to create more effective, targeted, and adaptable nanogenerators will be crucial for the success of this technology in clinical settings.

In conclusion, nanogenerators hold significant promise as transformative tools for neuroimmunomodulation, offering a novel approach to treating neurological diseases. However, unlocking their full potential requires overcoming challenges related to biocompatibility, refining targeting strategies, and establishing ethical frameworks for AI‐driven systems. As interdisciplinary collaboration accelerates—uniting materials science, immunology, and clinical medicine—the vision of a self‐regulating neuroimmune interface, powered by sustainable nanotechnology, moves closer to becoming a reality. This paradigm shift not only promises to treat diseases but also aims to redefine our understanding of health, where equilibrium is maintained through dynamic, technology‐augmented communication between mind and body. As research progresses, it is likely that nanogenerators will play an increasingly pivotal role in the precision treatment of neurological conditions, ultimately bridging the gap between basic scientific discoveries and clinical applications. The future of nanogenerators in neuroimmunomodulation holds immense potential, and with ongoing research and innovation, these technologies are poised to become an integral part of therapeutic strategies in neurology.

## Conflicts of Interest

The authors declare no conflicts of interest.

## Data Availability

The authors have nothing to report.
